# Effects of structured exercise training on miRNA expression in previously sedentary individuals

**DOI:** 10.1371/journal.pone.0314281

**Published:** 2024-12-18

**Authors:** Barbara Mayr, Michael Neudorfer, Daniela Wurhofer, Carolin Kilian, Eva-Maria Strumegger, Mahdi Sareban, Josef Niebauer

**Affiliations:** 1 University Institute of Sports Medicine, Prevention and Rehabilitation and Research Institute of Molecular Sports Medicine and Rehabilitation, Paracelsus Medical University, Salzburg, Austria; 2 Ludwig Boltzmann Institute for Digital Health and Prevention, Salzburg, Austria; Hong Kong Metropolitan University, HONG KONG

## Abstract

**Introduction:**

Micro ribonucleic acids (miRNA) respond to acute bouts of vigorous exercise, such as maximal cardiopulmonary exercise tests (CPET), by expressing an anti-atherogenic, anti-inflammatory and hence probably ergogenic profile. However, the impact of long-term engagement in physical exercise on CPET-induced miRNA response in sedentary individuals, with subsequent increased risk for cardiovascular diseases, remains unclear.

**Methods:**

Thirty-four sedentary participants underwent CPET before and after a four-month app-assisted exercise intervention, during which the moderate to vigorous physical activity (MVPA) was increased to over 150 min/week. Capillary blood samples were collected before and after CPET at baseline and after the exercise intervention. Twenty target miRNAs previously reported to be responsive to exercise and exercise adaptive pathways, or linked to atherogenic properties as inflammation, or previously identified upregulated following exercise in subjects with coronary artery disease versus healthy subjects were analyzed via real-time polymerase chain reaction.

**Results:**

Physical activity increased from 64 ± 48 to 354 ± 332 min/week of MVPA (p<0.001, +553%), accompanied by an improvement in maximal power output during CPET (ΔWatt_max_: 19 ± 13, p<0.001, +9%). Eleven of the selected twenty miRNAs showed significant responses to CPETs at either the beginning or end of the study. We found a significant increase both times for miR-103a (glycolysis, %change base: +12%, post +17%), miR-146a (inflammation, %change base: +20%, post +21%), and miR-222 (cardiac remodeling, %change base: +10%, post +21%), while miR-30a (inflammation, %change base: -27%, post: -38%) decreased significantly (all p≤0.043).

**Conclusion:**

Increased physical activity led to a significant CPET-induced change in three miRNAs from an atherogenic profile to a healthier one, indicating improved metabolic health and reduced inflammation.

## Introduction

Physical inactivity is increasing worldwide, resulting in higher numbers of cardiovascular diseases (CVD) [1, 2]. A sedentary lifestyle is defined as performing less than 150 minutes of moderate to vigorous intensity physical activity (MVPA) per week. As increasing physical activity has numerous benefits for preventing and treating CVD, guidelines recommend increasing weekly physical activity to more than 150 minutes per week [2, 3].

Despite the well-known beneficial effects of physical activity, not all underlying mechanisms have yet been fully uncovered. Circulating micro ribonucleic acids (miRNA) are known to regulate gene expression by translational inhibition and some miRNA patterns have shown to indicate certain physiological and pathological conditions [4]. MiRNAs are capable of modulating a vast number of cellular processes throughout the complete human lifespan, for example, as regulators for various pathways connected with energy provisioning, like fatty acid metabolism, pathways that are crucial for adaptation towards exercise. As previously described by our working group, circulating miRNAs known to be associated with anti-atherogenic and anti-inflammatory properties are responsive to an acute exercise stimulus like a maximal cardiopulmonary exercise test (CPET), which is a standardized and easy way to analyze acute responses to exercise [5–7]. Only a handful of other studies have been published investigating the acute reaction of circulating plasma miRNAs before and after a CPET or ergometry [8–11]. The number of publications increased when other forms of endurance interventions, like different intensities and lengths of running interventions, were used as exercise stimuli for the analysis of the acute effect on circulating plasma miRNAs [9, 12–28]. Further, only a few studies analyzed different forms of strength exercises [9, 29, 30]. However, within studies of both exercise types, the focus was very often on so called myomiRs (e.g. miR-1 or miR-133a), which are miRNAs closely related to muscle metabolism and functionality. When using cycling interventions [8–10, 31–34], the interest in miRNAs seemed to shift towards endothelial and inflammation-related miRNAs like miR-126 or miR-146a. The diversity of interventions even increases when analyzing the chronic effects of exercise over a more extended period [8, 10, 11, 31, 35–38]. Thus, a valid comparison between the studies is impossible due to the variety of exercise interventions. Circulating miRNAs are affected by exercise load, intensity, gender, and the presence of underlying diseases. As most of the present studies were conducted with healthy, trained male participants, the question arises, how miRNAs react in other collectives, like untrained or female participants. One of these collectives is previously sedentary healthy individuals, where little is known about how miRNA with anti-atherogenic and anti-inflammatory associated miRNAs are influenced by acute exercise in the form of CPET and how those miRNAs change the expression after prolonged structured exercise training. Gaining information on this topic might help to generate future molecular biomarkers. These miRNA expression profiles may help monitor the effect of exercise on metabolic health and/or cardiovascular risk and deliver, in addition, ways to gain further insights into the steering mechanism of energy allocations, hence allowing individual monitoring of exercise/training intensity. Therefore, we aimed to address this research gap with the present longitudinal cohort study.

## Materials and methods

### Ethical approval and study participants

The study was performed between the 1^st^ of July 2021 and the 31^st^ of July 2022 at the University Institute for Sports Medicine, Prevention and Rehabilitation of the Paracelsus Medical University Salzburg, Austria. Sedentary, healthy hospital employees (<150 min MVPA per week) participated in this four-month intervention study. Individuals were eligible if they were >18 years old and agreed to a four-month center-based and app-assisted exercise intervention. Written informed consent was obtained from all participants. The study complied with the declaration of Helsinki. The ethics committee of the state of Salzburg (1207/2020) approved this study, and the study was registered at ClinicalTrials.gov (NCT04791306).

### General study design

We conducted a prospective, longitudinal, and repeated measures study to examine circulating miRNA profiles in healthy, previously sedentary hospital employees (<150 min MVPA per week; evaluated via international physical activity questionnaire (IPAQ)). Participants were recruited via advertisement within the hospital intranet as well as via the corporate health program of the University Hospital Salzburg. Potential participants received the IPAQ questionnaires and in case of <150 min MVPA, an invitation for laboratory testing. Qualifying candidates were invited based on the first come, first serve principle. For the study inclusion, the IPAQ results established during the baseline examination were used. CPETs were performed before and after four months of increased physical activity (>150 min MVPA per week) consisting of once-a-week supervised center-based endurance and strength training and app-assisted individualized home-based training. An a priori sample size calculation gave out a suitable sample size of *n* = 39, including a dropout rate of 33% using the pre-CPET means, pre- and post-CPET standard deviations, and pre-post CPET correlations of relative expression values corresponding to 9 miRNAs taken from a previous unpublished study on acute changes with healthy volunteers of our working group.

### Laboratory testing

All participants underwent the following measurements at the beginning and end of the study. Measurements included anthropometrics (body mass, height, and body-mass index), spirometry, resting electrocardiogram (ECG), blood pressure, patient history, and blood testing (lipids, electrolytes, markers of kidney and liver function, glucose, and blood count). Further, body composition (BIA 101 Anniversary Sport Edition, Akern GmbH, Mainz, Germany) and a non-invasive pulse wave analysis using the portable automated oscillometric device Mobil-o-Graph^®^ (I.E.M. GmbH, Stolberg, Germany) were performed. Additionally, risk scores for cardiac events in the next ten years were evaluated using the PROCAM- [39], Framingham- [40], and ESC-Score [41]. Participants were asked to fill out standardized questionnaires on physical activity (IPAQ short form [42] and the rapid assessment of physical activity (RAPA [43]), general health (Health survey questionnaire: SF-36 [44]) as well as emotional well-being (WHO-5 [45]).

### Cardiopulmonary exercise testing

A breath-by-breath spirometer (Metalyzer 3B, Cortex Biophysik GmbH, Leipzig, Germany) assessed gas exchange and ventilation during maximal cycle CPET (Lode Excalibur sport, Lode B.V., Groningen, Netherlands). During the incremental testing, all participants were monitored using a 12-lead ECG (Amedtec ECGpro Version 5.10., AMEDTEC Medizintechnik Aue GmbH, Aue, Germany). Blood pressure and blood samples for blood lactate measurements were taken at every increment throughout the incremental tests. Lactate samples were analyzed using an enzymatic-amperometric chip sensor technology (Biosen C-line, EKF diagnostics, Cardiff, UK). Depending on sex and body weight, starting loads were chosen and subsequently increased by one-minute increments of 10–25 watts (W) to reach peak physical work capacity (PWC_peak_) after 8 to 12 min. The tests were terminated, when participants reached maximal exhaustion. All examinations were conducted in the morning after an overnight fast. Each analysis was repeated as performed during baseline testing at the end of the study.

### Exercise training

Supervised exercise training sessions were performed once a week for four months at the Institute of Sports Medicine, Prevention and Rehabilitation of the Paracelsus Medical University Salzburg, Austria. Each on-site training session consisted of cycle ergometer training followed by machine-based resistance training. All ergometer-training sessions were carried out on Ergoselect 200 (Ergoline GmbH, Bitz, Germany) and were recorded by Ergoline Reha Systems (Ergoline GmbH, Bitz, Germany). Participants performed a high-intensity interval training for 38 minutes: 4 × 4 minutes intervals at 85–95% peak heart rate, each followed by 3 minutes of active recovery at 60–70% peak heart rate, including 5 minutes warm-up and 5 minutes cool-down at 50–70% peak heart rate. Resistance training consisted of 3 sets of 12 reps at 80% of 10-repetition maximum on ten training machines, seven for the upper body (arm dips, seated rowing, chest press, cable pulls, lat pull-down, back extension, abdominal crunch) and three for the lower body (Leg press, leg extension, seated leg curl).

In addition, all participants were equipped with a smartphone application (app) called *aktivplan*, developed by the Ludwig Boltzmann Institute for Digital Health and Prevention, Salzburg [46]. This app provided individualized physical activity plans for each participant and was used to document the weekly amount of performed MVPA. In particular, participants were asked to document the duration, type, and subjectively experienced exertion (BORG scale) of each activity via the *aktivplan* app. Further, videos and messages were integrated into the app to motivate participants to perform physical activity. At the end of the study, all exercise-related information entered by participants was exported into csv files for further analysis by the research team.

The training period were successfully completed, when participants performed regular documented physical activity with a mean amount of more than 150 minutes per week for at least 16 weeks. Participants who did not reach the required documented amount were excluded from the analysis. The study was terminated early without a final examination, if participants were unreachable for more than three weeks, did not show up for trainings sessions, and did not document any other activities via the app. Further reasons for early dropouts were illness or injuries, which prevented participants to perform physical activity for a longer period of time.

For objective evaluation, if MVPA of more than 150 minutes per week was reached, each participant was equipped with a Garmin vivoactive 4 smartwatch (Garmin, Olathe, Kansas, USA) during the first and last week of study. Valid smartwatch measurements were assumed if at least 8 hours per day were recorded for at least six days a week. Heart rate data were classified as resting, light, moderate, or vigorous based on the recommendation by Visseren et al using the peak heart rate recorded during the maximal CPET as a reference [2].

### Sample collection and preparation

Blood samples for the miRNA analysis were drawn after an overnight fast immediately before and directly after maximal CPET. Two times, 200μl of EDTA blood was collected in capillary tubes out of the ear lobes (Microvette 200K3E, Sarstedt AG & Co.KG, Nümbrecht, Germany). Subsequently, collection tubes were centrifuged within one hour of blood collection for 15 min with 2650g. Blood plasma aliquots were stored at -80°C until analysis. Baseline and final examination were conducted at the same time of day.

### Selection of miRNA targets

Following miRNAs were chosen miR-150-5p, miR-101-3p, miR-141-3p, and miR-200b-3p due to their involvement with the previously described predictor model for coronary artery disease of our working group [5]. In addition, miR-29a-3p and miR-30a-5p were chosen based on their association with sudden cardiac death [47]. miR-126-3p as a marker for endothelial damage [9, 13, 48, 49], miR-21-3p due to its role in the adaption towards hypoxia and inflammation as well as its influence on muscle contractibility [8, 50–52], and miR-146a-5p because of the role in the adaption towards hypoxia and inflammation [8, 13, 31, 50, 53]. Further, we decided to analyze miR-145-5p and miR-143-3p because of their anti-atherogenic function and the influence on smooth muscle cell adaption [54], miR-223-3p due to its role in inflammatory processes [55], miR-222-3p because of exercise-induced cardiac remodeling [56] and miR-338-3p because of its previously described response to acute exercise [5, 31]. The following miRNAs were selected for normalization based on prior analysis: miR-148b-3p; miR-652-3p; miR-103a-3p; miR-107. For quality control the following targets were also analyzed in all samples: MiR-23a-3p and miR-451a (quotient used for assessment of hemolysis rate [57]), UniSp2, UniSp4 (RNA isolation control), UniSp6 (synthesis control) and UniSp3 (DNA spike-in as inhibition control). If samples showed deviant results compared to all, those samples were excluded for further analysis.

### RNA extraction

RNA extraction and analysis were conducted at Qiagen Genomic Services, Hilden, Germany. Briefly, plasma samples were thawed on ice and centrifuged at 4°C for 5 min (3000g). Total RNA, including miRNA, was isolated from 200μl plasma using miRNeasy Serum/Plasma Advanced Kit, high-throughput bead-based protocol v.1 (QIAGEN, Hilden, Germany) in an automated 96-well format. The purified total RNA was eluted in a final volume of 50μl.

### MiRNA quantification by a quantitative polymerase chain reaction

For quantitative polymerase chain reaction (qPCR), 2μl of each RNA sample was reversely transcribed using the miRCURY LNA RT Kit. cDNA was diluted 50x and assayed in 10μl PCR reactions according to the protocol for miRCURY LNA miRNA PCR; each miRNA was assayed once by qPCR on the miRNA Ready-to-Use PCR, Custom panel using miRCURY LNA SYBR Green master mix. Negative controls were performed and profiled like the samples, excluding the template from the reverse transcription reaction. The amplification was performed in a LightCycler^®^ 480 Real-Time PCR System (Roche, Basel, Switzerland) in 384-well plates. Roche LC software was used to analyze the amplification curves and determine the cycle threshold and melting curve analysis. Quantitative PCR experiments were conducted at Qiagen Genomic Services, Germany.

Concentrations for each miRNA were calculated via standard curves. Results were normalized with the global mean of the corresponding assays detected in all samples (relative miRNA expression). After evaluation, the global mean was chosen because it was superior for the normalization procedure to the average of the selected miRNA [58]. Expression levels at rest were shown in figures as the logarithm of the relative miRNA expression for better display. Relative expression values after maximal CPET were compared with baseline levels to evaluate exercise effects. Regulations are expressed as percent change versus resting levels.

### Pathway analysis

MiRNAs with a significant change of expression were uploaded to the DIANA-miRPath v4.0 web service app (https://dianalab.e-ce.uth.gr/tools last access 24.08.2024) to determine the KEGG pathways (Kyoto Encyclopedia of Genes and Genomes) which these miRNAs interact with, gaining more insight into the molecular pathways of exercise-affected miRNAs [59]. The following parameters were selected using the miRPath v4.0 tool to run the analysis. We chose to perform a miRNA-centric analysis, with selected targets from the TarBase v8.0 with direct TarBase targets for homo sapiens. As miRNA annotation miRbase-v22.1 was used, the chosen pathways were set to KEGG pathways with the selected merging method by genes union. Applying the classic analysis as testing method, with a p-value threshold of 0.05 and a false discovery rate correction.

### Statistical analysis

All data were analyzed for normal distribution using the Shapiro-Wilk test. If normally distributed, descriptive statistics are presented as means ± SD (standard deviation), respectively median and range if not normally distributed. MiRNA data are shown as a median with 95% CI (confidence interval). Paired samples were compared using a paired t-test or Wilcoxon signed-rank test, while gender comparisons were analyzed using the Student’s t-test or Mann-Whitney-U test, as well as repeated measure multi variance analysis, to evaluate possible sex differences over time. A Benjamini-Hochberg correction for multiple testing was performed to control for false-positive results during statistical analysis of the miRNA expression [60]. Correlation analyses were performed using Pearson’s respectively Spearman-Rho correlation based on distribution. SPSS version 27.0.1 (SPSS Inc., Chicago, Illinois; US) and GraphPad Prism version 8.3.0 (GraphPad Software Inc., San Diego, California, US) were used for statistical analyses. Statistical significance was assumed at p < 0.05.

## Results

### Subjects characteristics

**Fig 1:**
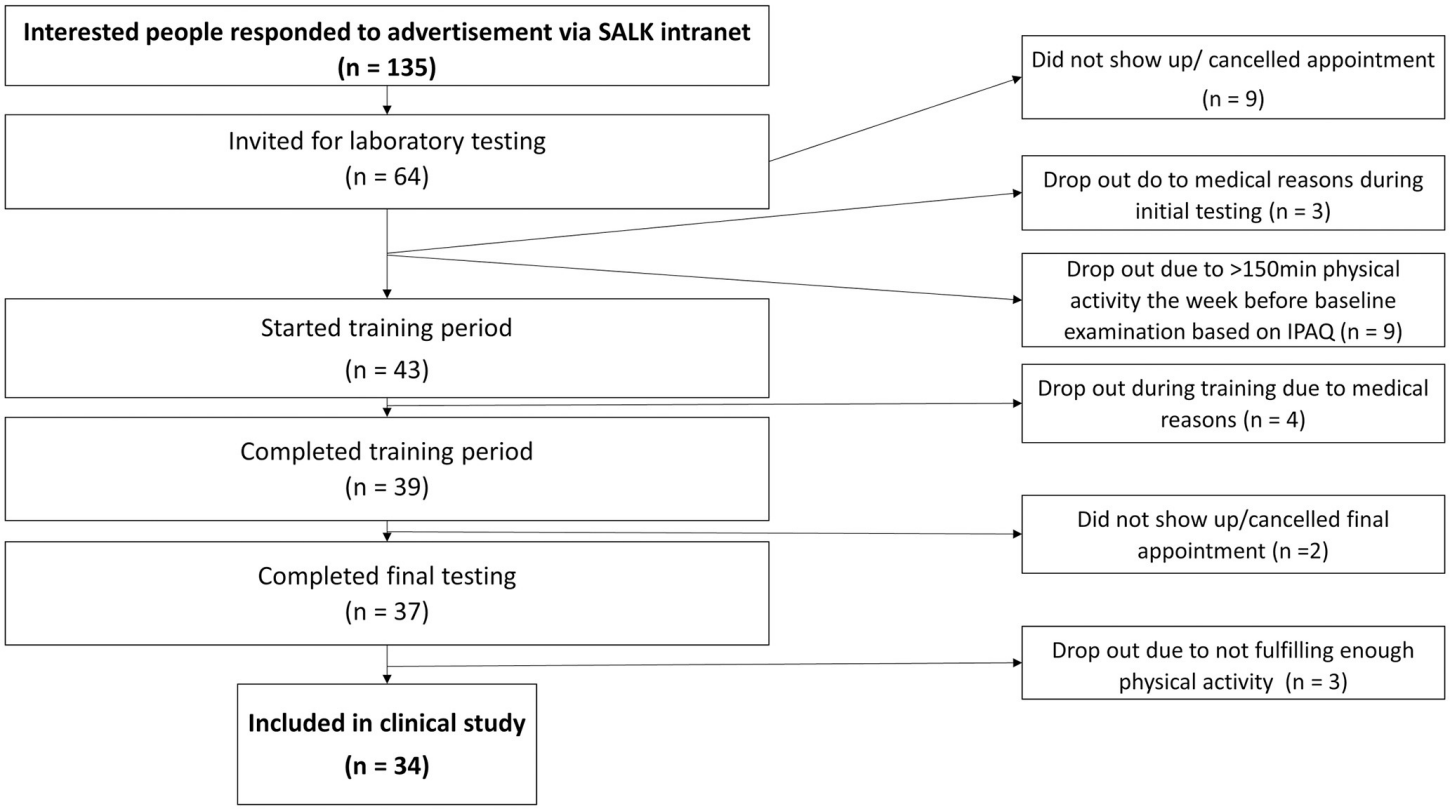
Flow diagram.

Sixty-four participants were invited, and 55 performed baseline examinations (30 female, 25 male). Forty-three participants fulfilled all inclusion criteria and started the four-month training period. Finally, 37 participants completed the training period and the final examination. Three additional participants were excluded from the final data analysis since they did not reach the minimal amount of MVPA documented via the *aktivplan* app. The final number of 34 participants (19 female and 15 male; median age 34 years (20–60 years)) and an overall dropout rate of 21% remained. The baseline characteristics of study participants included in the analysis are shown in Table 1. A flow diagram is shown in Fig 1.

**Table 1:** Subject characteristics.

parameter	All N = 34	Male N = 15	Female N = 19	p-value
Age [years]	34 (20–60)	44 (20–59)	30 (22–60)	0.082†
Weight [kg]	72.7 ± 13.1	79.0 ± 12.7	67.7 ± 11.5	0.010*
Height [cm]	172.4 ± 7.7	178.2 ± 5.9	167.8 ± 5.5	<0.001*
Body mass index [kg/m^2^]	24.4 ± 3.9	24.9 ± 3.8	24.1 ± 4.1	0.573
MVPA based on IPAQ [min]	64 ± 48	70 ± 47	59 ± 49	0.523†

Values are presented as arithmetic mean ± SD, p-values are calculated with Student’s t-test or if not normal distributed Mann-Whitney U test (marked with †) * p<0.05 Abbreviations: MVPA = moderate to vigorous physical activity; IPAQ = international physical activity questionnaire; There were no significant changes within the anthropometric data at the end of the study.

### Exercise parameter

The participants significantly increased their aerobic exercise capacity ($\dot{V}{\text{O}}_{2\text{peak}}$) (difference baseline versus the end of the study: +2.0 ± 1.8 ml/min/kg; +6.2 ± 5.4%; p<0.001) as well as their PWC_peak_ (difference baseline vs. end of the study: +19 ± 13 watts; +9.1 ± 5.7%; p<0.001). In addition, the workload at submaximal lactate thresholds improved significantly (all p≤ 0.015), while heart rates at these thresholds remained essentially unchanged (all p≥ 0.436; Table 2).

**Table 2:** Comparison of exercise parameter baseline versus end of study N = 34.

	baseline	post	p-value	%change
Aerobic exercise capacity [ml/min/kg]	33.7 ± 6.0	35.7 ± 6.4	<0.001*	+6
Aerobic exercise capacity [L/min]	2.4 ± 0.6	2.6 ± 0.7	<0.001*	+5
Maximal minute ventilation (V’E) [L/min]	93.4 ± 25.0	101.8 ± 28.2	<0.001*	+9
Respiratory exchange ratio (RER)	1.11 ± 0.06	1.14 ± 0.06	0.001*	+3
Peak work capacity [W]	211 ± 56	230 ± 61	<0.001*	+9
Power per bodyweight [W/kg]	2.9 ± 0.6	3.2 ± 0.7	<0.001*	+9
Heart rate max [1/min]	175 ± 12	175 ± 12	0.406	0
Lactate max [mmol/ml]	10.1 ± 2.5	11.1 ± 2.3	0.003*	+13
BORG (RPE)	18 ± 1	19 ± 1	<0.001*†	+6
reference performance [%]	131 ± 23	142 ± 23	<0.001*	+9
MVPA based on IPAQ [min]	64 ± 48	354 ± 332	<0.001*†	+553
Lactate thresholds				
Power at LT [W]	84 ± 24	93 ± 32	0.015*†	+11
HR at LT [1/min]	121 ± 17	119 ± 13	0.629	-0
Power at IAS [W]	121 ± 31	132 ± 39	0.004*†	+9
HR at IAS [1/min]	138 ± 16	136 ± 14	0.436	-1
Power at 2 mmol lactate [W]	105 ± 35	118 ± 35	0.001*	+19
HR at 2 mmol lactate [1/min]	130 ± 16	130 ± 14	0.918	+0
Power at 3 mmol lactate [W]	135 ± 39	147 ± 40	<0.001*	+12
HR at 3 mmol lactate [1/min]	143 ± 14	143 ± 23	0.761	0
Ventilation thresholds				
Oxygen uptake at VT1 [L/min]	1.4 ± 0.4	1.5 ± 0.5	0.125	+6
Power at VT1 [W]	98 ± 34	101 ± 43	0.265	+7
HR at VT1 [1/min]	123 ± 16	122 ± 17	0.348	-0
Oxygen uptake at VT2 [L/min]	2.2 ± 0.6	2.3 ± 0.7	0.002*	+6
Power at VT2 [W]	174 ± 53	187 ± 57	0.001*	+8
HR at VT2 [1/min]	159 ± 17	159 ± 16	0.378	+0

Values are presented as arithmetic mean ± SD, p-values are calculated with paired t-test or if not normal distributed Wilcoxen signed-rank test (marked with †) * p<0.05 Abbreviations: V’E = minute ventilation; RER = respiratory exchange ratio; W = watt; RPE = rate of perceived exertion; MVPA = moderate to vigorous physical activity; IPAQ = international physical activity questionnaire; LT = lactate threshold; IAS = individual aerobic threshold; 2mmol = lactate threshold of 2mmol per liter; 3mmol = lactate threshold of 3mmol per liter; HR = heart rate; VT1 = first ventilation threshold; VT2 = second ventilation threshold; %change = mean difference in percent compared to baseline.

During the 18-week training phase, participants performed a median amount of physical activity of 200 (152–682) minutes per week, documented via the *aktivplan* app. A comparison between the first and the last week of training is shown in Table 3.

**Table 3:** Comparison of documented exercise amount between first and last week of training (N = 34).

	First week	Last week	p-value	%change
Documented time in ***aktivplan*** app [min]	224 ± 143	228 ± 188	0.567†	+2
Recorded Smartwatch MVPA [min] (N = 23)	267 ± 215	246 ± 267	0.378†	-7
MVPA based on IPAQ [min] (week before baseline examination)	64 ± 48	354 ± 332	<0.001*†	+553
Mean workload supervised ergometer training [W]	99 ± 28	113 ± 31	<0.001*	+15
Mean heart rate supervised ergometer training [1/min]	142 ± 16	142 ± 12	0.981	+1

Values are presented as arithmetic mean ± SD, p-values are calculated with paired t-test or if not normal distributed Wilcoxen signed-rank test (marked with †) * p<0.05 Abbreviations: W = watt; IPAQ = international physical activity questionnaire; MVPA = moderate to vigorous physical activity; %change = difference compared to baseline

The self-reported amount of MVPA correlated significantly with the peak aerobic exercise capacity and the peak physical work capacity as well as with the physical work capacity at the different lactate thresholds (Table 4).

**Table 4:** Spearman-Rho correlations between self-reported MVPA and CPET results at baseline examination as well as post training examination (N = 34).

	$\dot{V}{\text{O}}_{2\text{peak}}$ base	PWC_peak_ base	LT W base	IAS W base	2mmol W base	3mmol W base
IPAQ MVPA	.530**	.516**	.412*	.478**	.573***	.563***
$\dot{V}{\text{O}}_{2\text{peak}}$	-	.687***	.484**	.507**	.568***	.585***
PWC_peak_	-	-	.709***	.811***	.690***	.696***
LT W	-	-	-	.576***	.818***	.844***
IAS W	-	-	-	-	.910***	.940***
2mmol W	-	-	-	-	-	.989***
	** $\dot{V}O_{2peak}$ ** **POST**	**PWC**_**peak**_ **POST**	**LT W** **POST**	**IAS W** **POST**	**2mmol W** **POST**	**3mmol W** **POST**
IPAQ MVPA	.393*	.445**	.501**	.455**	.431**	.429*
$\dot{V}{\text{O}}_{2\text{peak}}$	-	.753***	.627***	.626***	.560***	.618***
PWC_peak_	-	-	.846***	.886***	.851***	.904***
LT W	-	-	-	.977***	.910***	.931***
IAS W	-	-	-	-	.947***	.970***
2mmol W	-	-	-	-	-	.982***

*p<0.05;

**p<0.01;

***p<0.001.

Abbreviations: CPET = cardiopulmonary exercise test; IPAQ = international physical activity questionnaire, MVPA = moderate to vigorous activity, $\dot{V}{\text{O}}_{2\text{peak}}$ = peak oxygen uptake; PWC_peak_ = peak physical work capacity, LT = lactate threshold, IAS = Individual anaerobic threshold, 2mmol = lactate threshold of 2mmol per liter; 3mmol = lactate threshold of 3mmol per liter; W = watt

### Effects of acute exercise on miRNA levels

**Fig 2:**
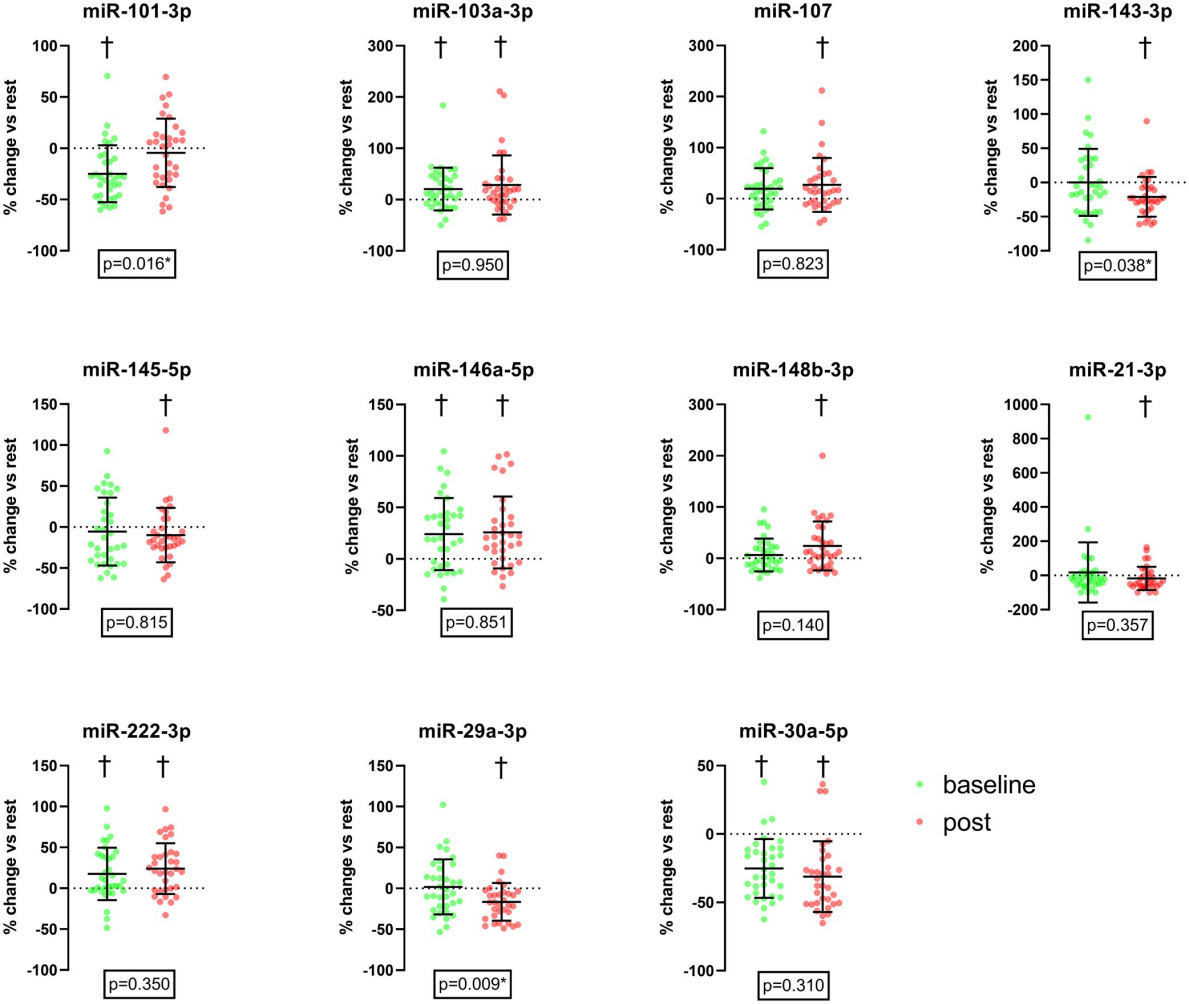
Relative changes of miRNA expression following maximal cardiopulmonary exercise tests compared to resting values at baseline and final examination. neg. values = down-regulation after exercise, pos. values = up-regulation after exercise † = significant acute change of expression level, p-values = comparison acute response between baseline and final examination; * = significant difference of acute response comparing baseline and final examination.

While analyzing the acute response of CPET on the expression level of the selected miRNAs, we found that five miRNAs were altered significantly during the baseline examination. miR-146a-5p (Δ: 20% [95%CI: 12, 36]), miR-222-3p (Δ: 10% [95%CI: 6, 29]), and miR-103a-3p (Δ: 12% [95%CI: 6, 35]) showed a significant increase after the baseline CPET compared to the resting levels. Whereas miR-101-3p (Δ: -30% [95%CI: -36, -16]) and miR-30a-5p (Δ: -27% [95%CI: 32, 18]) were significantly decreased at the same time (Fig 2).

**Fig 3:**
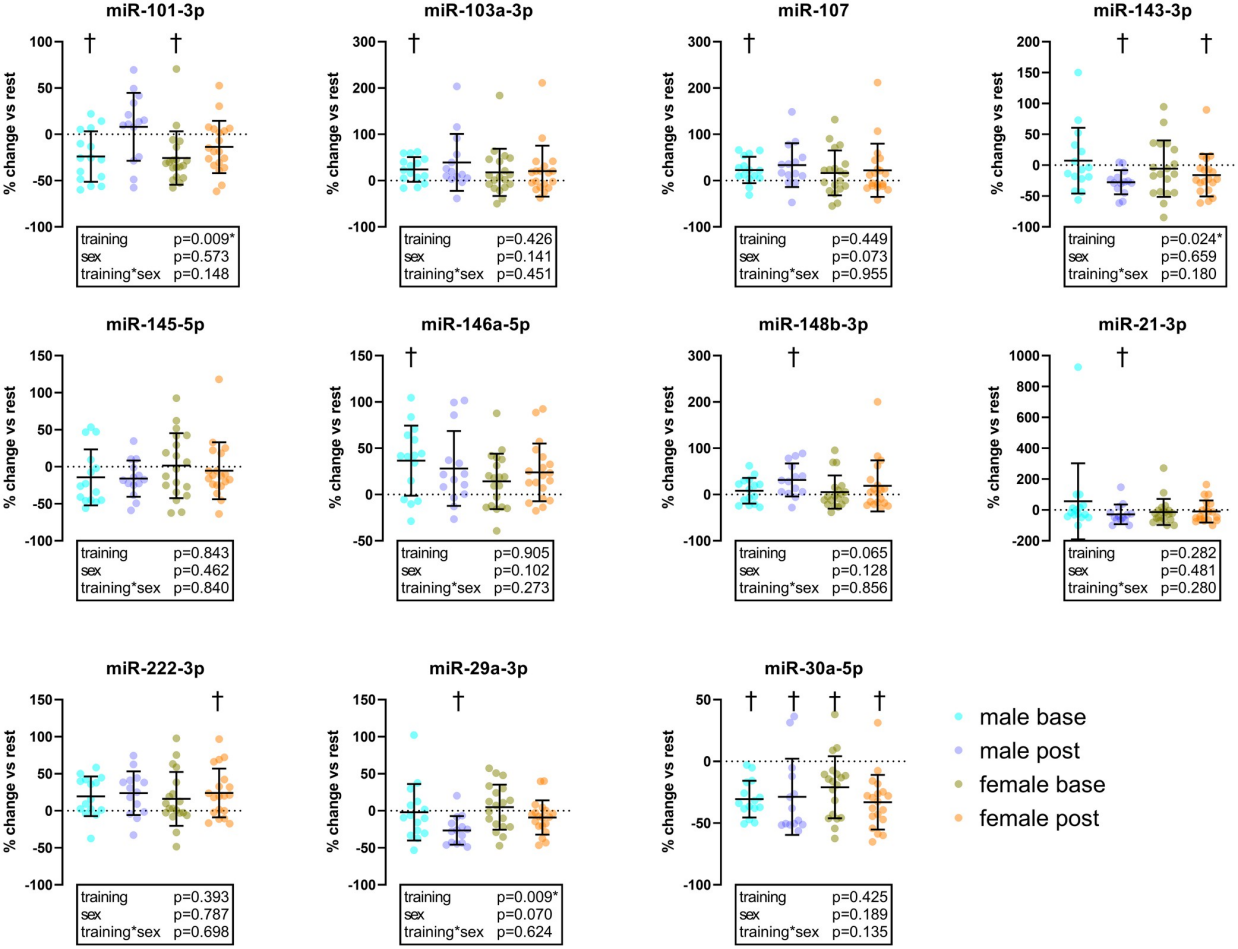
Relative changes of miRNA expression following maximal cardiopulmonary exercise tests compared to resting values at baseline and final examination separated by sex. neg. values = down-regulation after exercise, pos. values = up-regulation after exercise † = significant acute change of expression level within the group, p-values = results of ANOVA comparison of acute response between baseline and final examination; *p<0.050.

After four months of increased training, the analysis of the expression levels before and after the final examination showed a significant increase in miR-103a-3p (Δ: 17% [95%CI: 8, 49]), miR-107 (Δ: 15% [95%CI: 8, 46]), miR-148b-3p (Δ: 12% [95%CI: 7, 41]), miR-146a-5p (Δ: 21% [95%CI: 13, 38]) and miR-222-3p (Δ: 21% [95%CI: 13, 35]). As well as a significant down-regulation of the expression levels of miR-143-3p (Δ: -25% [95%CI: -31, -11]), miR-145-5p (Δ: -16% [95%CI: -22, 2]), miR-21-3p (Δ: -43% [95%CI: -42, 6]) miR-29a-3p (Δ: -17% [95%CI: -25, -8]) and miR-30a-5p (Δ: -38% [95%CI: -40, -22]) (Fig 2). The same findings separated by male and female are shown in Fig 3.

### Effects of structured exercise training on miRNA levels

No significant changes were found when comparing the resting levels at baseline with those after four months of sustained structured exercise training.

Significant alterations in the miRNA response to CPET were found when comparing baseline changes with changes after four months of structured exercise training. Significant down-regulation of the anti-atherogenic miR-101-3p seen during baseline examination was not found during the final examination (Δchange: -30% [95%CI: -36, -16] to -7% [95% CI: -16, 7]; p = 0.016). On the other hand, the anti-atherogenic miR-143-3p (Δchange: -7% [95%CI: -17, 18] to -25% [95%CI: -31, -11]; p = 0.038), as well as the sudden cardiac death related miR-29a-3p (Δchange: -1% [95%CI: -10, 14] to -17% [95%CI: -25, -8]; p = 0.009), showed significant down-regulations only during the final examination but not during baseline. A graphical overview of all responses can be found in the S1 Fig.

### Pathway analysis of significantly altered miRNAs

When analyzing the three miRNAs, which significantly changed their expression in response to exercise between baseline and end of the study, and uploading the data to DIANA-miRPath v4.0, 96 KEGG pathways were identified (false discovery rate of <0.05). Results are shown in Table 5. A graphical overlap of shared target genes is shown as Venn diagram in the S2 Fig.

**Table 5:** KEGG pathway analysis for the three miRNAs, which significant changes in the exercise response after 4 months of increased exercise.

*KEGG pathway name*	*# pathway genes*	*# target genes*
*Focal adhesion*	213	67
*Protein processing in endoplasmic reticulum*	194	61
*Proteoglycans in cancer*	220	66
*Shigellosis*	268	76
*p53 signaling pathway*	75	31
*Pathways in cancer*	555	131
*Hippo signaling pathway*	164	52
*Cell cycle*	129	44
*PI3K-Akt signaling pathway*	372	95
*Viral carcinogenesis*	265	73
*Adherens junction*	79	31
*Autophagy—animal*	146	45
*Renal cell carcinoma*	70	27
*FoxO signaling pathway*	139	43
*Colorectal cancer*	88	31
*Neurotrophin signaling pathway*	124	39
*Rap1 signaling pathway*	214	58
*Regulation of actin cytoskeleton*	224	60
*Salmonella infection*	277	70
*Pathogenic Escherichia coli infection*	222	59
*Ubiquitin mediated proteolysis*	142	42
*Bacterial invasion of epithelial cells*	80	28
*AGE-RAGE signaling pathway in diabetic complications*	115	36
*Oocyte meiosis*	134	40
*Ras signaling pathway*	241	62
*Cellular senescence*	219	57
*Apoptosis—multiple species*	32	15
*Endocytosis*	311	75
*Dopaminergic synapse*	143	41
*Small cell lung cancer*	100	31
*Pathways of neurodegeneration—multiple diseases*	539	115
*Fluid shear stress and atherosclerosis*	149	41
*Alzheimer disease*	426	94
*Hedgehog signaling pathway*	59	21
*Apoptosis*	151	41
*Tight junction*	182	47
*EGFR tyrosine kinase inhibitor resistance*	82	26
*MAPK signaling pathway*	329	75
*AMPK signaling pathway*	130	36
*Glioma*	79	25
*Platinum drug resistance*	75	24
*Thyroid hormone signaling pathway*	137	37
*Yersinia infection*	147	39
*Progesterone-mediated oocyte maturation*	104	30
*Kaposi sarcoma-associated herpesvirus infection*	245	58
*Longevity regulating pathway*	105	30
*Pancreatic cancer*	78	24
*Apelin signaling pathway*	140	37
*Chronic myeloid leukemia*	79	24
*Human papillomavirus infection*	406	87
*Signaling pathways regulating pluripotency of stem cells*	156	40
*Hepatocellular carcinoma*	177	44
*Amyotrophic lateral sclerosis*	408	87
*Hepatitis C*	173	43
*Relaxin signaling pathway*	138	36
*Hippo signaling pathway—multiple species*	30	12
*Prolactin signaling pathway*	73	22
*Oxytocin signaling pathway*	161	40
*mTOR signaling pathway*	177	43
*Sphingolipid signaling pathway*	133	34
*Endocrine and other factor-regulated calcium reabsorption*	57	18
*Gap junction*	99	27
*Long-term potentiation*	71	21
*Human immunodeficiency virus 1 infection*	277	61
*Melanoma*	76	22
*Prostate cancer*	101	27
*Alcoholism*	195	45
*cGMP-PKG signaling pathway*	175	41
*Amphetamine addiction*	74	21
*Spinocerebellar ataxia*	145	35
*Endometrial cancer*	61	18
*Measles*	161	38
*Adrenergic signaling in cardiomyocytes*	161	38
*Platelet activation*	136	33
*Mitophagy—animal*	76	21
*Phosphatidylinositol signaling system*	101	26
*Fc gamma R-mediated phagocytosis*	101	26
*Insulin signaling pathway*	153	36
*Endocrine resistance*	118	29
*Parathyroid hormone synthesis, secretion and action*	118	29
*Hepatitis B*	177	40
*Lysine degradation*	69	19
*Arrhythmogenic right ventricular cardiomyopathy*	79	21
*Huntington disease*	339	69
*N-Glycan biosynthesis*	51	15
*Breast cancer*	163	37
*TNF signaling pathway*	131	31
*Circadian entrainment*	100	25
*ErbB signaling pathway*	86	22
*Renin secretion*	72	19
*Human cytomegalovirus infection*	306	62
*Insulin resistance*	124	29
*Protein digestion and absorption*	114	27
*Leukocyte transendothelial migration*	120	28
*T cell receptor signaling pathway*	115	27
*Longevity regulating pathway—multiple species*	79	20

KEGG pathway analysis using DIANA-miRPath v4.0, with a false discovery rate of <0.05. Depicted are the numbers of genes within the pathway, as well as number of target genes within the pathway, by miR-101-3p, miR-143-3p and miR-29a-3p.

The following KEGG pathways showed the most relevant target gene interactions for possible adaption processes due to increased exercise: The KEGG pathway for fluid shear stress and atherosclerosis (endothelial adaption); mechanistic target of rapamycin (mTOR; protein turnover, cell energy balance, and oxygen concentration); mitogen-activated protein kinase (MAPK; cell proliferation, cell differentiation, cell stress); cyclic guanosine 3’, 5’-monophosphate dependent protein kinase G (cGMP-PKG; relaxation and contraction of vascular smooth muscle cell, anti-vascular injury); adenosine monophosphate-activated protein kinase (AMPK; cellular energy homeostasis); phosphoinositide-3-kinase protein kinase B (PI3K-Akt; regulation of cell cycle, metabolism, angiogenesis); and forkhead box O (FoxO; glucose metabolism, oxidative stress) [59].

### Correlation analysis of miRNAs and exercise parameters

Analyzing correlations between miRNA expression levels and exercise parameters, we found significant moderate correlations between resting levels of miR-103a and heart rate at the lactate threshold (LT) as well as the individual anaerobic threshold (IAS) (LT base: r_s_ = 0.342, p = 0.048, IAS base: r_s_ = 0.350, p = 0.043; LT post: r_s_ = 0.395, p = 0.021; IAS post: r_s_ = 0.379, p = 0.027). Further, post-CPET levels of miR-103a and $\dot{V}{\text{O}}_{2\text{peak}}$ (base: r = 0.355, p = 0.039; post: r_s_ = 0.345, p = 0.049) correlated significantly during both exercise tests. The strongest significant negative correlation was found between expression levels of miR-29a post-CPET at the end of the study with corresponding $\dot{V}{\text{O}}_{2\text{peak}}$ (r = -0.547; p = 0.001) and PWC_peak_ (r = -0.436; p = 0.011) values. The strongest significant positive correlations were found between miR-107 resting levels at the end of the study compared to PWC_peak_ (r = 0.462; p = 0.006), percent of the reference norm for working power (r = 0.570; p< 0.001) and the power at 3mmol lactate thresholds (r_s_ = 0.527, p = 0.001). All correlation results are shown in S1 Table.

## Discussion

To the best of our knowledge, this is the first study that examines expression changes of circulating miRNA following maximal CPET at baseline and after increasing physical activity over 4 months in previously sedentary individuals.

### Exercise parameter

All study participants increased their self-reported weekly amount of MVPA from 64 ± 48 min at the baseline examination to 354 ± 332 min (p<0.001) at the final examination, with a median weekly amount of 200 (152–682) min per week. Both times, the self-reported amounts of MVPA correlated significantly with the results of the corresponding CPET tests (Table 4). The structured exercise training led to an increase in maximal and submaximal exercise capacity.

### Effects of acute exercise on miRNA levels

During baseline CPETs, 5 miRNAs, and during the end of study CPETs, 10 miRNA of the 20 selected miRNAs showed significant changes in expression. Unexpectedly, fewer miRNAs responded to baseline CPETs at first sight than in our previous studies [5, 7]. This difference might be explained by the fact that our initial studies were performed on physically active participants. Our data suggest that miRNA expression in response to a CPET differs between physically active versus physically inactive individuals. These results further suggest that regular physical activity influences the molecular response to exercise. As shown here, twice as many miRNAs reacted in the final examination.

Furthermore, 4 miRNAs (miR-103a, miR-146a, miR-222, miR-30a) significantly changed their expression levels during both CPETs at baseline and study end, apparently acute exercise is capable to trigger the release or uptake of those circulating miRNAs independently from a physically active lifestyle. As they react to the exercise stimulus both times, they might regulate essential pathways for exercise adaptions. Opening up the possibility of using those miRNAs as potential monitoring tools for exercise/training intensity.

An overexpression of miR-103a in cancer cells led to increased extracellular lactate concentration, enhancing glycolysis [61]. As glycolysis is one of the critical processes to generate energy during physical activity, we hypothesize that similar mechanisms occur during the performance of a CPET, as miR-103a increases significantly after CPET. We further found significant correlations between miR-103a expression levels and the maximal lactate concentration and other exercise parameters (Table 5). These findings suggest that miR-103a might have the potential to serve as a marker for evaluating energy allocation during acute exercise.

As exercise can trigger a systemic inflammatory response, miRNAs like miR-146a or miR-30a, which function as key regulators during such processes, adapt their expression levels accordingly [8, 13, 47]. In the present study, we found a significant increase directly after CPET for the inflammatory key regulator miR-146a, a miRNA described as a natural brake on the NF-κB (nuclear factor kappa-light-chain-enhancer of activated B cell) pathway. This pathway is of utmost importance for muscle metabolism, as it can induce inflammation or fibrosis and block myofiber regeneration after injuries when activated [62]. Previous studies showed the reactivity towards acute all-out exercise for miR-146a as well. Baggish et al. showed significant increases due to all-out exercise within this miRNA in their collective of young trained males [8]. In contrast, van Craenenbroeck et al. described significant down-regulation of miR-146a in their collective of chronic kidney disease patients after acute exercise [10]. As the NF-κB pathway is already activated in patients with kidney disease, this might explain the apparent discrepancy. Further, we found a significant decrease of miR-30a in response to both CPETs. As this miRNA is associated with protective/adaptive responses towards stressors of inflammation, it might function as a gatekeeper of the inflammatory cascade [47] triggered by exercise.

Endurance training leads to exercise-induced cardiac adaptation, and miR-222 is described to play a major part in this adaption process by influencing the angiogenesis in skeletal muscle [8, 63]. Similar to our present results, Baggish et al. described a significant increase of miR-222 both during acute exercise and after acute exercise following sustained aerobic exercise training [8].

### Effects of 4-month structured exercise training on resting miRNA levels

There were no significant changes in the resting levels of circulating miRNAs after four months of structured exercise training. These results are in line with the findings of van Craenenbroeck et al., as they also found no change in the resting levels after chronic exercise intervention [10]. In contrast, Baggish et al. described an increase in resting levels of miR-146a, miR-21, and miR-222 after 90 days of intensive rowing training [8], whereas Nielsen et al. reported a significant increase in resting levels of miR-103 and miR-107 and a decrease for miR-148b after 12 weeks of endurance cycling training five times per week in healthy trained men [31]. The discrepancy between those results might be due to the age difference but also because participants of Baggish et al. and Nielsen et al. were trained men, and therefore, the training intensity and volume were higher. In contrast, our study population, and the one of van Craenenbroeck et al., consisted of less active individuals. On the other hand, Witvrouwen et al. described a significant decrease in resting levels of miR-146a in heart failure patients after 15 weeks of endurance and strength training [11]. Further, Parr et al. showed that the resting levels of miR-223 increased after a 16 week diet and exercise intervention [36]. As these studies showed, underlying diseases like heart failure or obesity may influence the resting levels of miRNAs. A further hint that the fitness level, as well as the type of training, might play a crucial part in the amount of resting expression levels can be seen with miR-222, as this miRNA is further described to be expressed in higher resting levels in endurance-trained athletes compared to strength trained athletes and untrained controls [50]. However, even the type of strength training influences the expression levels of miR-222 differently over time, as shown by Horak et al, who described a decrease due to explosive strength training, whereas hypertrophic strength training led to an increase after five weeks [35]. Interestingly, we found significantly lower resting expression levels among participants who were classified as having a fitness status of fair or better based on the definition by the American College of Sports Medicine, compared to those with poor or very poor fitness [64].

### miRNA response following maximal CPET after 4-months structured exercise training

Three miRNAs (miR-101, miR-143, miR-29a) significantly changed the acute reaction when comparing baseline and final examination responses. When comparing these findings with previously conducted examinations of our working group, we saw the following due to 4 months of structured exercise training. The expression levels of two of the three miRNA shifted from a profile seen in coronary artery disease patients after CPET (decrease in miR-101, increase in miR-29a) to those seen in healthy individuals [5], underlining once more the preventive properties of regular physical exercise.

Gaining further insights into what might have changed on a molecular level due to increased weekly physical activity; we took a closer look at those miRNAs, which only changed significantly in response to the CPET after four months (107, 148b, 143, 145, 21, 29a) but not at the beginning. Therefore, we used the miR-Path tool to perform a KEGG pathway analysis using just those six miRNAs. By doing this, we found pathways, which might have increased their performance due to increased MVPA, subsequently resulting in a higher demand of the target miRNAs for regulation of these pathways. For example, the KEGG pathway analysis revealed that those miRNAs (107, 148b, 143, 145, 21, 29a) can interact with 14 out of 31 mRNA targets of the citrate cycle and 50 out of the 122 mRNA targets in the carbon metabolism. Both pathways are crucial in energy provisioning, especially during exercise, and hence might describe the ergogenic properties of epigenetic adaptions [59]. A full list of KEGG pathways is shown within the S2 Table.

Correlating the expression levels post-exercise of those miRNAs with the reached exercise parameters at the end of the study, we found amongst others that miR-107 showed a significant positive correlation with PWC_peak_ (r_s_ = 0.453; p = 0.008), whereas miR-29a showed significant negative correlation for exercise capacity (r = -0.547; p = 0.001) and PWC_peak_ (r = -0.436; p = 0.011).

Previous research showed that a decrease in miR-148b plays a role in the progression of atherosclerosis [65], which leads to the assumption that the increase after maximal CPET after 4-month of structured exercise training might have regulatory functions on the proliferation of vascular smooth muscle cells and thus might help to prevent progression of atherosclerosis. Smooth muscle cells are required to switch the phenotype to adapt to physiological and pathological process changes. Literature showed that miR-143 and miR-145 are important molecular factors for this phenotype switch [66]. By decreasing in circulation, as seen in this present study, we assume that an uptake of those miRNAs by smooth muscle cells might have occurred, possibly resulting in regulatory processes in connection with these phenotype switches.

Taken together, we showed that an increase in regular physical activity increases the number of exercise-responsive miRNAs. We further reported that independent of the presence or absence of a physically active lifestyle, specific miRNAs are triggered by acute exercise, influencing glycolysis, inflammation, and skeletal muscle adaption pathways. An increase in regular physical activity further affected miRNAs, which have more known targets within a pathway, essential for exercise adaptation processes. Further studies must investigate how these exercise-induced changes in miRNA profiles predict laboratory surrogates for metabolic health (like e.g., lipid profile, insulin, HbA1c) and, thus, if they might help personalizing lifestyle recommendations for cardiovascular risk reduction and monitoring of exercise/training load.

### Limitations

As the IPAQ asks for the activity of the previous seven days, it is susceptible for special occasions like, for example, vacations, which might increase the weekly activity, even though in day-to-day life, 150 min of physical activity was not reached. Therefore, several potential participants had to be excluded from the analysis due to such occurrences prior to the baseline examination. Replacement for individuals who did not pass the IPAQ criteria was not possible, as nationwide lockdowns due to the COVID-19 pandemic were initiated during the runtime of the study, leading to an underpowered sample size overall. A further limitation of this present study is that it was conducted as a single-arm study, and we lack a control group without lifestyle changes.

## Supporting information

S1 Fig Overview of all miRNA expression comparisons. Upward arrow = increased expression; downward arrow = decreased expression; dark arrows = significant alteration.
(TIF)

S2 Fig VENN diagram of overlapping number of protein targets by miR-101-3p, miR-143-3p and miR-29a-3p. (TIF)

S3 Fig Relative changes of miRNA expression following maximal cardiopulmonary exercise tests compared to resting values at baseline and final examination separated by fitness level. neg. values = down-regulation after exercise, pos. values = up-regulation after exercise † = significant acute change of expression level within the group, p-values = results of ANOVA comparison of acute response between baseline and final examination; *p<0.050; ACSM = American college of sports medicine.
(TIF)

S4 Fig Relative changes of miRNA expression following maximal cardiopulmonary exercise tests compared to resting values at baseline and final examination separated by median age. neg. values = down-regulation after exercise, pos. values = up-regulation after exercise † = significant acute change of expression level within the group, p-values = results of ANOVA comparison of acute response between baseline and final examination; *p<0.050; < = under median age; > = over median age.
(TIF)

S5 Fig Relative changes of miRNA expression following maximal cardiopulmonary exercise tests compared to resting values at baseline and final examination separated by sex and fitness level. neg. values = down-regulation after exercise, pos. values = up-regulation after exercise.
(TIF)

S6 Fig Distribution of age, sex and fitness level during baseline examination. (TIF)

S1 TableCorrelation analysis between miRNA expression levels and exercise parameters at baseline examination as well as post training examination (N = 34).miRNA expression at baseline were correlated only with baseline measurements and miRNA expression at the end of the study (end) were correlated only with end of study values. *p<0.05; **p<0.01; ***p<0.001. Abbreviations: resting = pre-exercise measurements, post CPET = post exercise measurements; Δ = differences between pre and post exercise measurements; base = baseline examination; end = end of study examination; CPET = cardiopulmonary exercise test; r = Pearson’s Correlation coefficient, r_s_ = Spearman-Rho Correlation coefficient, $\dot{V}{\text{O}}_{2\text{peak}}$ = peak oxygen uptake; PWC = physical work capacity in watt, LT = lactate threshold, IAS = Individual anaerobic threshold, 2mmol = lactate threshold of 2mmol per liter; 3mmol = lactate threshold of 3mmol per liter; HR = heart rate; %Ref.Norm = percent based on reference performance norm.(DOCX)

S2 Table KEGG pathway analysis for the five miRNAs, which significant changes in the exercise response only after 4 months of increased exercise. KEGG pathway analysis using DIANA-miRPath v4.0, with a false discovery rate of <0.05. Depicted are the numbers of genes within the pathway, as well as numbers of target genes within the pathways, by miR-107, miR-148b-3p; miR-143-3p; miR-145-5p; miR-21-3p and miR-29a-3p.
(DOCX)

S1 File Original study protocol (German). (PDF)

S2 File Translated study protocol. (PDF)

S3 File Raw data. (XLSX)

S4 File STROBE checklist cohort. (DOCX)

S5 File TREND checklist. (PDF)
