# Supplementary figures and images for: Effects of structured exercise training on miRNA expression in previously sedentary individuals
Source: PLoS One. 2024 Dec 18;19(12):e0314281. doi: 10.1371/journal.pone.0314281 (PMC11654927; doi:10.1371/journal.pone.0314281)

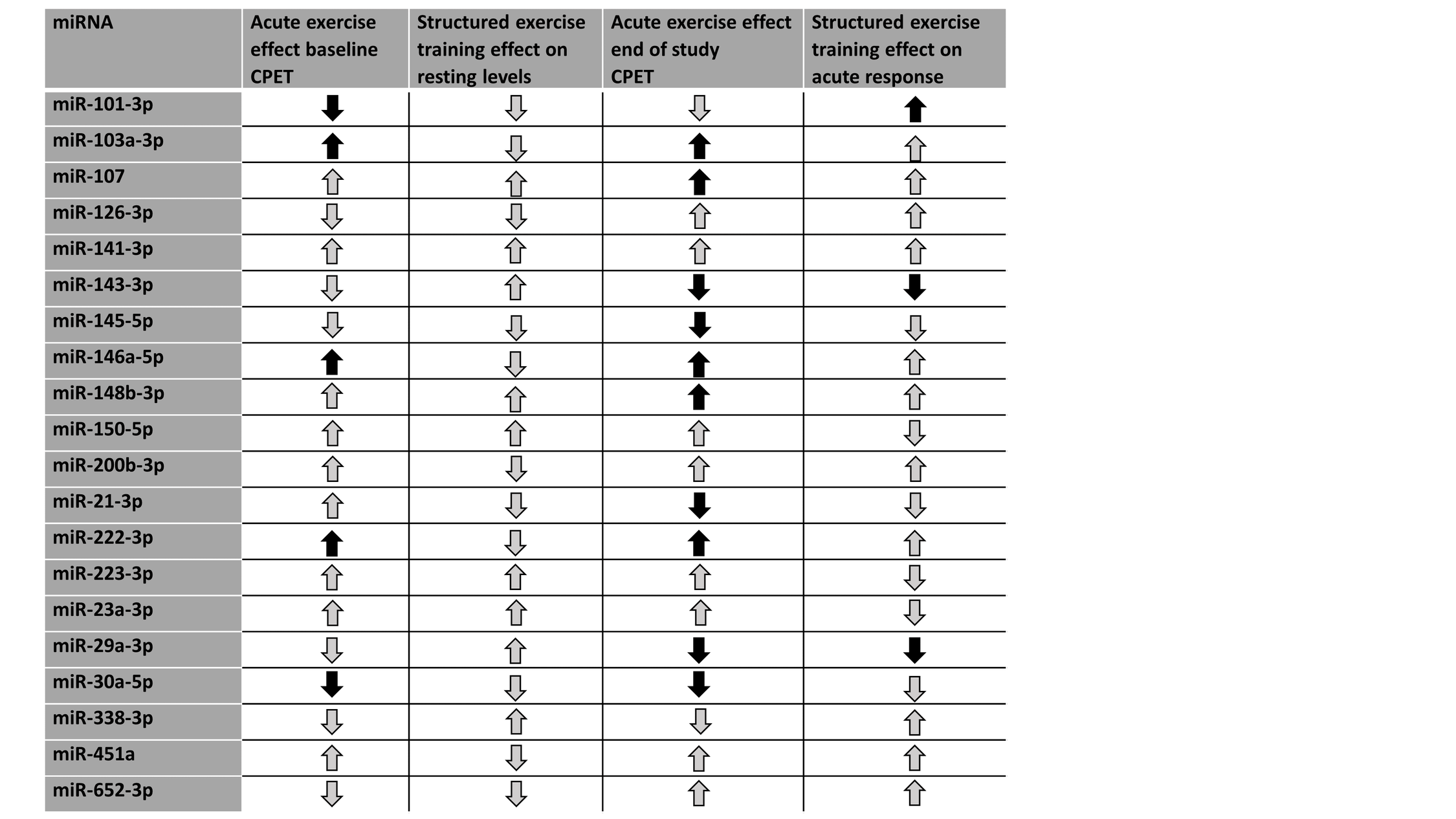

Supplement: S1 Fig — Upward arrow = increased expression; downward arrow = decreased expression; dark arrows = significant alteration. (TIF) [file pone.0314281.s001.tif]

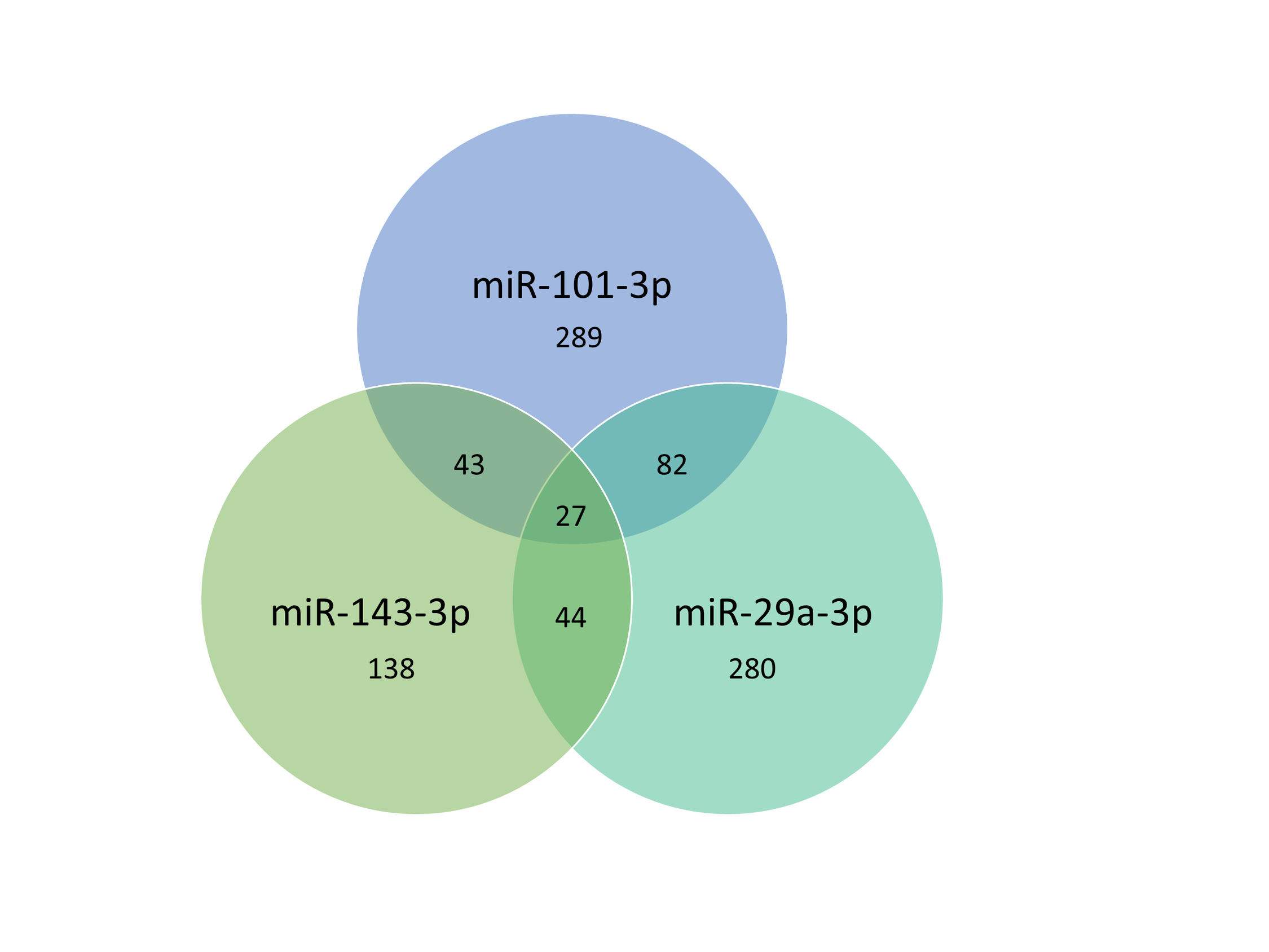

Supplement: S2 Fig — (TIF) [file pone.0314281.s002.tif]

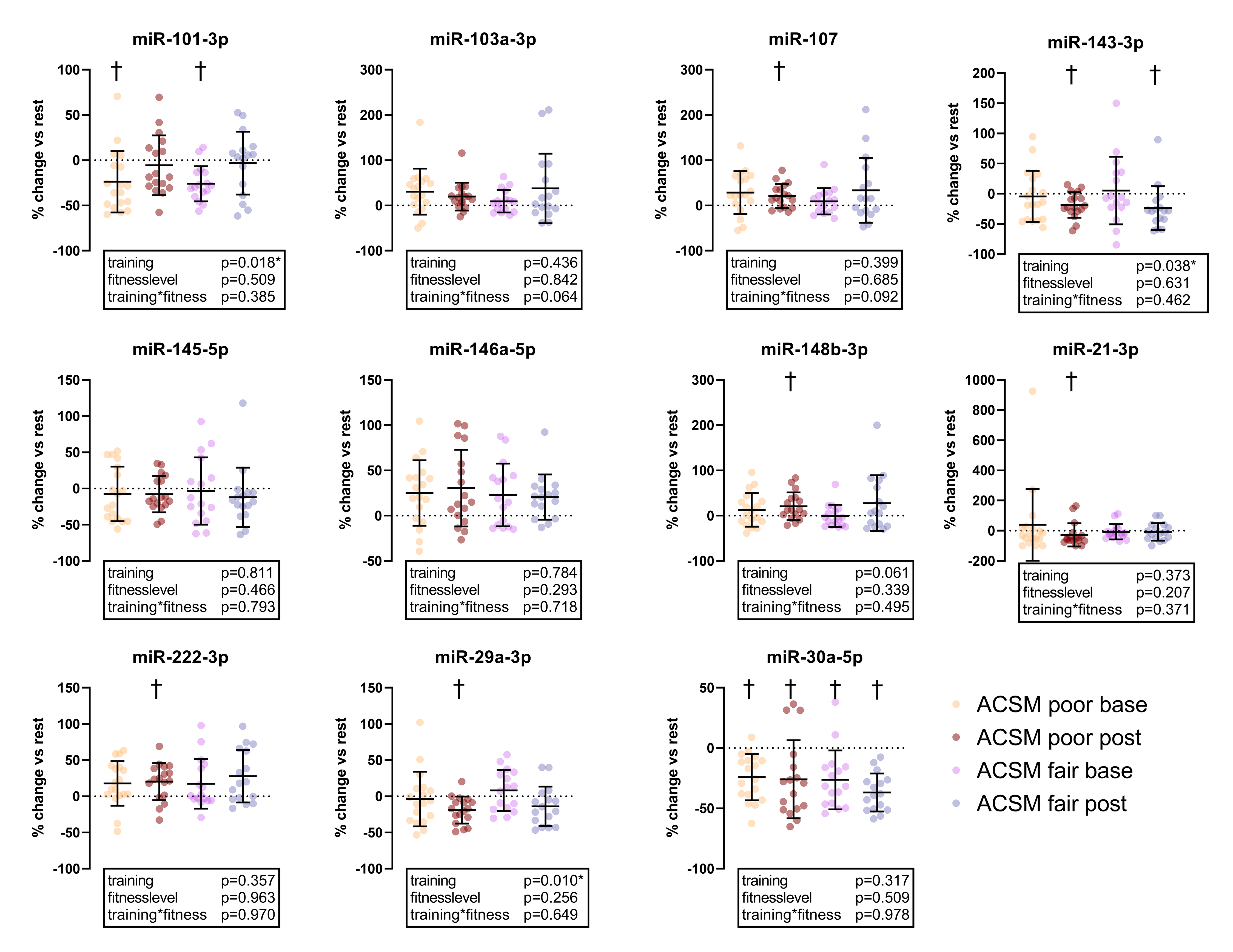

Supplement: S3 Fig — neg. values = down-regulation after exercise, pos. values = up-regulation after exercise † = significant acute change of expression level within the group, p-values = results of ANOVA comparison of acute response between baseline and final examination; *p<0.050; ACSM = American college of sports medicine. (TIF) [file pone.0314281.s003.tif]

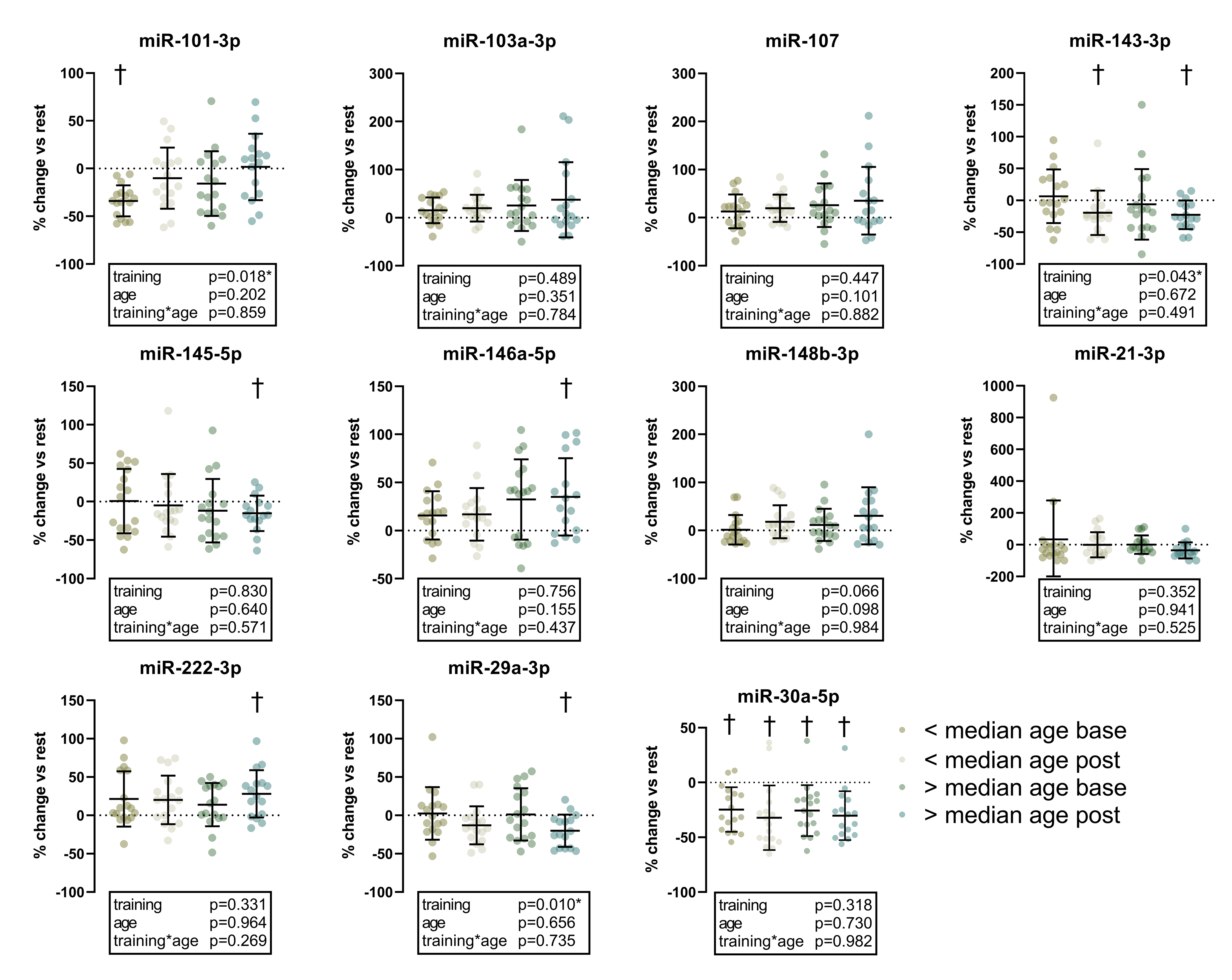

Supplement: S4 Fig — neg. values = down-regulation after exercise, pos. values = up-regulation after exercise † = significant acute change of expression level within the group, p-values = results of ANOVA comparison of acute response between baseline and final examination; *p<0.050; < = under median age; > = over median age. (TIF) [file pone.0314281.s004.tif]

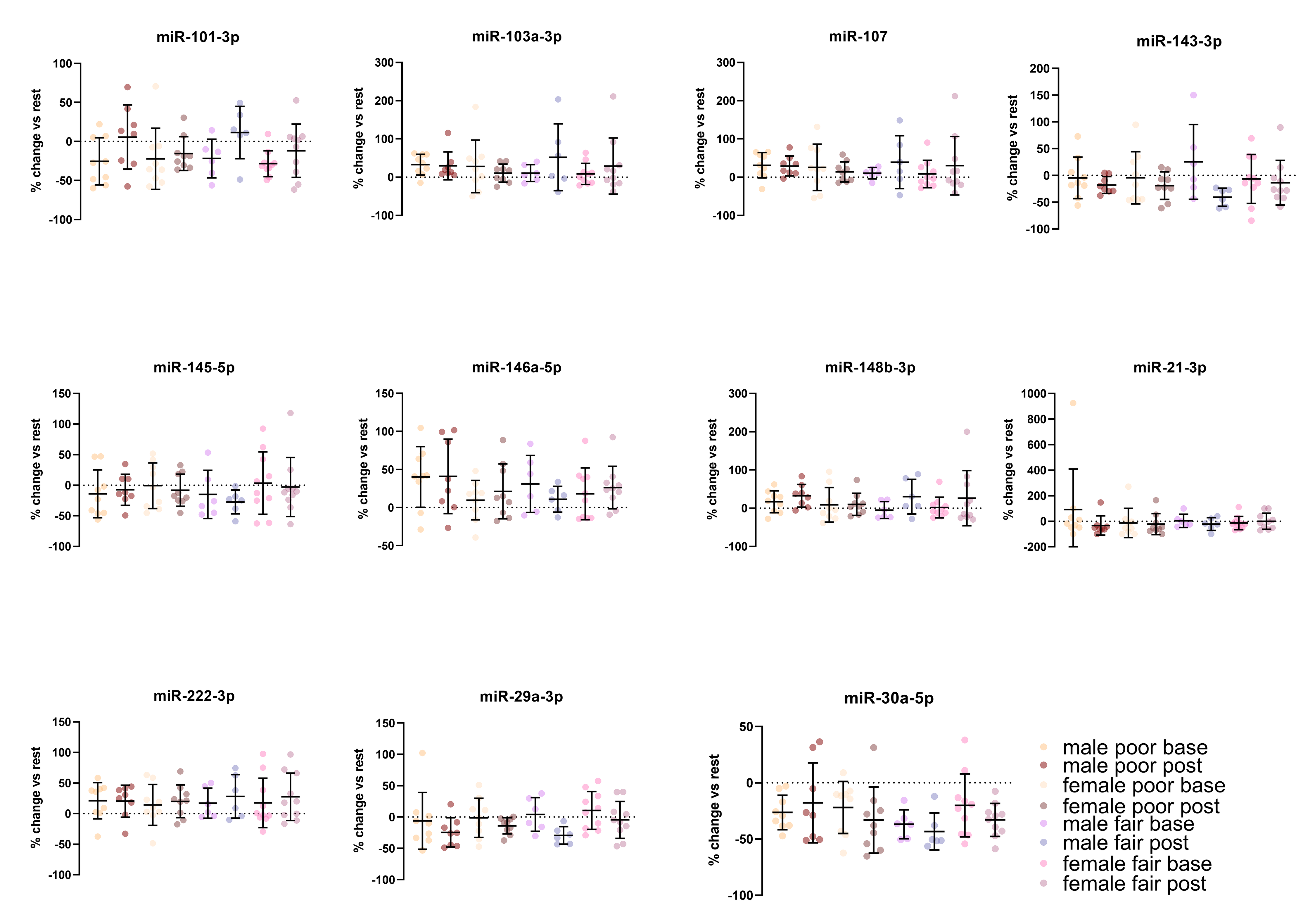

Supplement: S5 Fig — neg. values = down-regulation after exercise, pos. values = up-regulation after exercise. (TIF) [file pone.0314281.s005.tif]

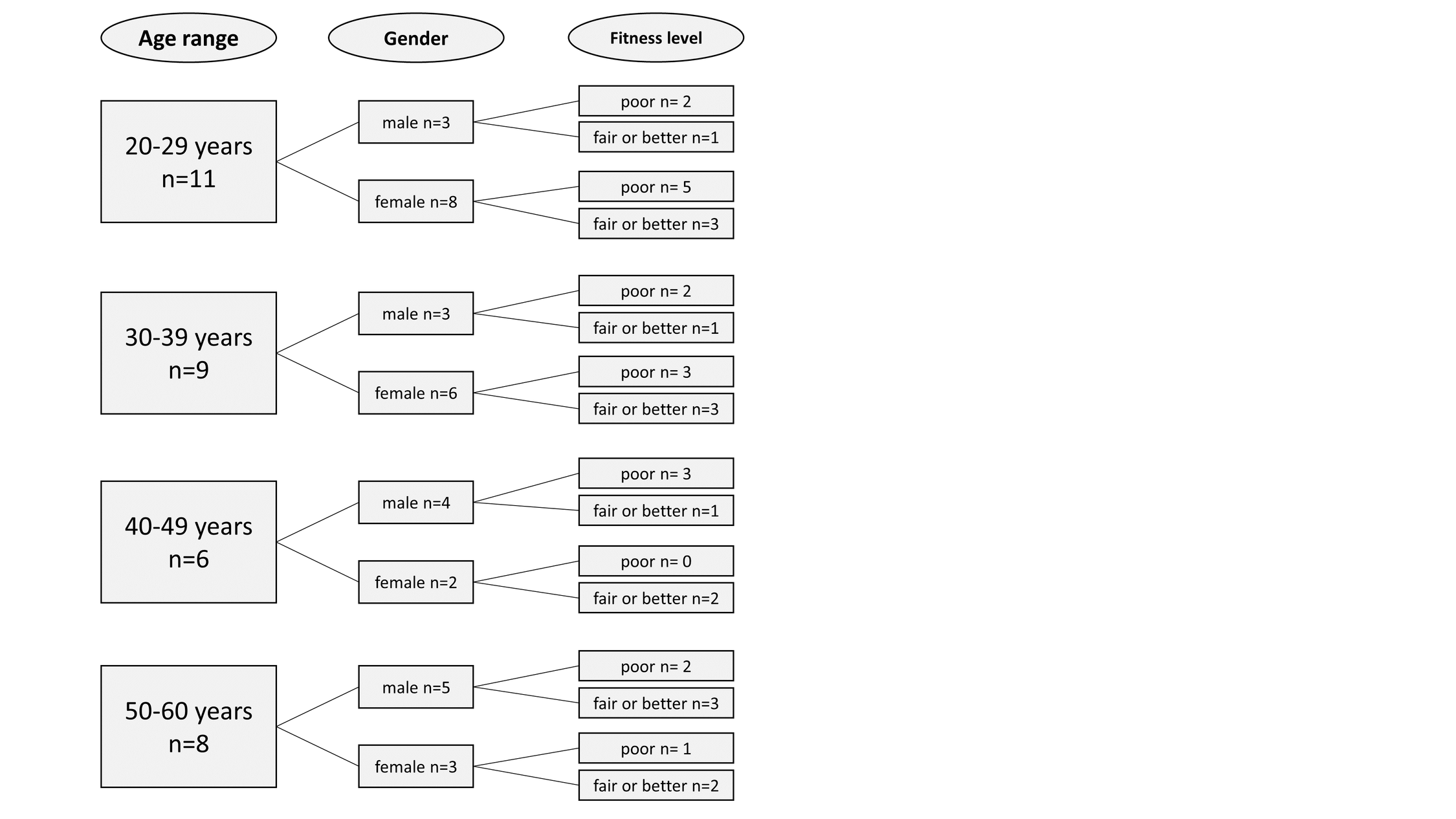

Supplement: S6 Fig — (TIF) [file pone.0314281.s006.tif]
