# Supplementary material for: Effects of structured exercise training on miRNA expression in previously sedentary individuals
Source: PLoS One. 2024 Dec 18;19(12):e0314281. doi: 10.1371/journal.pone.0314281 (PMC11654927; doi:10.1371/journal.pone.0314281)
Supplement: S1 Table — miRNA expression at baseline were correlated only with baseline measurements and miRNA expression at the end of the study (end) were correlated only with end of study values. *p<0.05; **p<0.01; ***p<0.001. Abbreviations: resting = pre-exercise measurements, post CPET = post exercise measurements; Δ = differences between pre and post exercise measurements; base = baseline examination; end = end of study examination; CPET = cardiopulmonary exercise test; r = Pearson’s Correlation coefficient, rs = Spearman-Rho Correlation coefficient, V˙O2peak = peak oxygen uptake; PWC = physical work capacity in watt, LT = lactate threshold, IAS = Individual anaerobic threshold, 2mmol = lactate threshold of 2mmol per liter; 3mmol = lactate threshold of 3mmol per liter; HR = heart rate; %Ref.Norm = percent based on reference performance norm. (DOCX) [file pone.0314281.s007.docx]

**S1 Table: Correlation analysis between miRNA expression levels and exercise parameters at baseline examination as well as post training examination (N=34)** miRNA expression at baseline were correlated only with baseline measurements and miRNA expression at the end of the study (end) were correlated only with end of study values

|  | **resting**  **base** | **post CPET**  **base** | **Δ**  **base** | **resting**  **end** | **post CPET**  **end** | **Δ**  **end** |
| --- | --- | --- | --- | --- | --- | --- |
| **miR-101-3p** | | | | | | |
| Age | r_s_= 0.319 | r= 0.348* | r= 0.101 | r= 0.146 | r_s_= 0.270 | r= 0.122 |
| **⩒**O_2peak_ | r_s_= -0.041 | r= -0.138 | r= -0.033 | r= 0.010 | r_s_= 0.171 | r= 0.068 |
| PWC_peak_ | r_s_= -0.146 | r= -0.144 | r= 0.007 | r= 0.110 | r_s_= 0.024 | r= -0.121 |
| PWC_LT_ | r_s_= 0.005 | r_s_= -0.181 | r_s_= -0.137 | r_s_= -0.151 | r_s_= -0.044 | r_s_= 0.118 |
| PWC_IAS_ | r_s_= 0.013 | r= -0.174 | r= -0.120 | r_s_= -0.153 | r_s_= -0.016 | r_s_= 0.150 |
| PWC_2mmol_ | r_s_= -0.056 | r= -0.028 | r= -0.076 | r= 0.018 | r_s_= -0.006 | r= -0.085 |
| PWC_3mmol_ | r_s_= -0.049 | r= -0.072 | r= -0.076 | r= -0.002 | r_s_= 0.017 | r= -0.052 |
| HR_peak_ | r_s_= -0.238 | r= -0.297 | r= -0.040 | r= 0.122 | r_s_= 0.102 | r= -0.143 |
| HR_LT_ | r_s_= -0.121 | r= -0.316 | r= -0.173 | r= 0.000 | r_s_= -0.210 | r= -0.157 |
| HR_IAS_ | r_s_= -0.060 | r= -0.303 | r= -0.224 | r= -0.049 | r_s_= -0.155 | r= -0.107 |
| HR_2mmol_ | r_s_= -0.064 | r= -0.193 | r= -0.153 | r= 0.078 | r_s_= -0.127 | r= -0.184 |
| HR_3mmol_ | r_s_= -0.118 | r= -0.247 | r= -0.174 | r= 0.017 | r_s_= -0.123 | r= -0.120 |
| %Ref.Norm | r_s_= -0.150 | r= -0.153 | r= 0.169 | r= 0.252 | r_s_= 0.056 | r= -0.323 |
| lactat_max_ | r_s_= -0.163 | r= -0.191 | r= 0.103 | r= 0.379* | r_s_= 0.112 | r= -.0271 |
| **miR-103a-3p** | | | | | | |
| Age | r_s_= -0.101 | r= -0.131 | r= -0.104 | r_s_= 0.075 | r_s_= 0.007 | r_s_= -0.160 |
| **⩒**O_2peak_ | r_s_= 0.218 | r= 0.355* | r= 0.123 | r_s_= 0.204 | r_s_= 0.345* | r_s_= 0.268 |
| PWC_peak_ | r_s_= -0.023 | r= 0.254 | r= 0.251 | r_s_= 0.260 | r_s_= 0.418* | r_s_= 0.222 |
| PWC_LT_ | r_s_= 0.147 | r_s_= 0.105 | r_s_= 0.109 | r_s_= 0.291 | r_s_= 0.295 | r_s_= 0.028 |
| PWC_IAS_ | r_s_= 0.044 | r= 0.199 | r= 0.117 | r_s_= 0.275 | r_s_= 0.313 | r_s_= 0.060 |
| PWC_2mmol_ | r_s_= 0.009 | r= 0.135 | r= 0.140 | r_s_= 0.308 | r_s_= 0.319 | r_s_= 0.015 |
| PWC_3mmol_ | r_s_= -0.009 | r= 0.166 | r= 0.183 | r_s_= 0.286 | r_s_= 0.326 | r_s_= 0.055 |
| HR_peak_ | r_s_= 0.230 | r= 0.290 | r= 0.159 | r_s_= 0.206 | r_s_= 0.391* | r_s_= 0.325 |
| HR_LT_ | r_s_= 0.342* | r= 0.127 | r= -0.073 | r_s_= 0.395* | r_s_= 0.248 | r_s_= -0.012 |
| HR_IAS_ | r_s_= 0.350* | r= 0.063 | r= -0.122 | r_s_= 0.379* | r_s_= 0.286 | r_s_= 0.028 |
| HR_2mmol_ | r_s_= 0.209 | r= 0.095 | r= 0.001 | r_s_=0.337 | r_s_= 0.291 | r_s_= 0.066 |
| HR_3mmol_ | r_s_= 0.136 | r= 0.061 | r= -0.026 | r_s_= 0.346* | r_s_= 0.305 | r_s_= 0.068 |
| %Ref.Norm | r_s_= -0.001 | r= 0.149 | r= 0.133 | r_s_= 0.154 | r_s_= 0.385* | r_s_= -0.062 |
| lactat_max_ | r_s_= 0.202 | r= 0.330 | r= 0.201 | r_s_= 0.066 | r_s_= 0.460** | r_s_= 0.445** |
| **miR-107** | | | | | | |
| Age | r= 0.140 | r_s_= 0.101 | r= -0.002 | r= 0.338 | r_s_= 0.299 | r_s_= 0.002 |
| **⩒**O_2peak_ | r= 0.256 | r_s_= 0.382* | r= 0.146 | r= 0.285 | r_s_= 0.336 | r_s_= 0.189 |
| PWC_peak_ | r= 0.226 | r_s_= 0.288 | r= 0.197 | r= 0.462** | r_s_= 0.453** | r_s_= 0.167 |
| PWC_LT_ | r_s_= 0.167 | r_s_= 0.122 | r_s_= 0.062 | r_s_= 0.410 * | r_s_= 0.311 | r_s_= -0.047 |
| PWC_IAS_ | r= 0.237 | r_s_= 0.187 | r= 0.090 | r_s_= 0.392* | r_s_= 0.349* | r_s_= 0.017 |
| PWC_2mmol_ | r= 0.161 | r_s_= 0.208 | r= 0.145 | r= 0.475 ** | r_s_= 0.386* | r_s_= 0.017 |
| PWC_3mmol_ | r= 0.186 | r_s_= 0.215 | r= 0.168 | r= 0.527** | r_s_= 0.405* | r_s_= 0.061 |
| HR_peak_ | r= 0.162 | r_s_= 0.156 | r= 0.072 | r= 0.046 | r_s_= 0.141 | r_s_= 0.282 |
| HR_LT_ | r= -0.045 | r_s_= -0.153 | r= -0.139 | r= 0.187 | r_s_= -0.027 | r_s_= -0.120 |
| HR_IAS_ | r= -0.030 | r_s_= -0.133 | r= -0.167 | r= 0.155 | r_s_= 0.016 | r_s_= -0.057 |
| HR_2mmol_ | r= -0.095 | r_s_= 0.031 | r= -0.023 | r= 0.137 | r_s_= 0.081 | r_s_= 0.030 |
| HR_3mmol_ | r= -0.064 | r_s_= -0.070 | r= -0.066 | r= 0.144 | r_s_= 0.076 | r_s_= 0.034 |
| %Ref.Norm | r= -0.017 | r_s_= 0.170 | r= 0.077 | r= 0.570*** | r_s_= 0.325 | r_s_= -0.134 |
| lactat_max_ | r= 0.129 | r_s_= 0.302 | r= 0.175 | r= 0.029 | r_s_= 0.307 | r_s_= 0.303 |
| **miR-126-3p** | | | | | | |
| Age | r_s_= 0.019 | r= 0.282 | r= 0.182 | r= 0.102 | r= 0.152 | r_s_= 0.251 |
| **⩒**O_2peak_ | r_s_= 0.034 | r= 0.214 | r= 0.084 | r= 0.157 | r= 0.183 | r_s_= 0.102 |
| PWC_peak_ | r_s_= 0.006 | r= 0.244 | r= 0.145 | r= 0.166 | r= 0.226 | r_s_= 0.103 |
| PWC_LT_ | r_s_= 0.201 | r_s_= 0.095 | r_s_= -0.085 | r_s_= 0.094 | r_s_= -0.042 | r_s_= -0.096 |
| PWC_IAS_ | r_s_= 0.150 | r= 0.168 | r= -0.061 | r_s_= 0.087 | r_s_= 0.016 | r_s_= -0.041 |
| PWC_2mmol_ | r_s_= 0.045 | r= 0.200 | r= 0.086 | r= 0.142 | r= 0.110 | r_s_= 0.042 |
| PWC_3mmol_ | r_s_= 0.026 | r=0.197 | r= 0.075 | r= 0.138 | r= 0.105 | r_s_= 0.052 |
| HR_peak_ | r_s_= -0.032 | r= -0.098 | r= 0.009 | r= -0.019 | r= -0.030 | r_s_= 0.021 |
| HR_LT_ | r_s_= 0.165 | r= -0.210 | r= -0.281 | r= 0.136 | r= -0.189 | r_s_= -0.297 |
| HR_IAS_ | r_s_= 0.152 | r= -0.296 | r= -0.315 | r= 0.066 | r= -0.266 | r_s_= -0.309 |
| HR_2mmol_ | r_s_= 0.007 | r= -0.134 | r= -0.093 | r= 0.110 | r= -0.136 | r_s_= -0.154 |
| HR_3mmol_ | r_s_= 0.002 | r= -0.241 | r= -0.164 | r= 0.054 | r=-0.227 | r_s_= -0.225 |
| %Ref.Norm | r_s_= -0.111 | r= 0.026 | r= 0.040 | r= 0.206 | r= 0.088 | r_s_= 0.094 |
| lactat_max_ | r_s_= 0.051 | r= 0.296 | r= 0.284 | r= 0.171 | r= 0.385* | r_s_= 0.293 |
| **miR-141-3p** | | | | | | |
| Age | r_s_= -0.483** | r_s_= -0.345* | r_s_= -0.089 | r= -0.134 | r_s_= -0.104 | r_s_= -0.044 |
| **⩒**O_2peak_ | r_s_= -0.029 | r_s_= -0.054 | r_s_= -0.064 | r= 0.089 | r_s_= -0.047 | r_s_= -0.175 |
| PWC_peak_ | r_s_= -0.024 | r_s_= -0.087 | r_s_= -0.055 | r= 0.128 | r_s_= -0.100 | r_s_= -0.218 |
| PWC_LT_ | r_s_= -0.132 | r_s_= 0.041 | r_s_= 0.129 | r_s_= 0.096 | r_s_= 0.122 | r_s_= 0.002 |
| PWC_IAS_ | r_s_= -0.140 | r_s_= -0.008 | r_s_= 0.104 | r_s_= 0.076 | r_s_= 0.053 | r_s_= -0.053 |
| PWC_2mmol_ | r_s_= -0.130 | r_s_= -0.064 | r_s_= 0.060 | r= 0.139 | r_s_= 0.003 | r_s_= -0.135 |
| PWC_3mmol_ | r_s_= -0.117 | r_s_= -0.029 | r_s_= 0.084 | r= 0.134 | r_s_= -0.014 | r_s_= -0.138 |
| HR_peak_ | r_s_= 0.114 | r_s_= 0.068 | r_s_= -0.017 | r= 0.232 | r_s_= -0.107 | r_s_= -0.250 |
| HR_LT_ | r_s_= 0.146 | r_s_= 0.278 | r_s_= 0.122 | r= 0.175 | r_s_= 0.206 | r_s_= 0.064 |
| HR_IAS_ | r_s_= 0.118 | r_s_= 0.278 | r_s_= 0.151 | r= 0.206 | r_s_= 0.202 | r_s_= 0.029 |
| HR_2mmol_ | r_s_= 0.149 | r_s_= 0.037 | r_s_= -0.063 | r= 0.194 | r_s_= 0.071 | r_s_= -0.100 |
| HR_3mmol_ | r_s_= 0.086 | r_s_= 0.193 | r_s_= 0.103 | r= 0.226 | r_s_= 0.112 | r_s_= -0.062 |
| %Ref.Norm | r_s_= -0.100 | r_s_= 0.049 | r_s_= 0.091 | r= 0.092 | r_s_= -0.019 | r_s_= -0.171 |
| lactat_max_ | r_s_= 0.050 | r_s_= -0.252 | r_s_= -0.314 | r= 0.075 | r_s_= -0.280 | r_s_= -0.298 |
| **miR-143-3p** | | | | | | |
| Age | r_s_= -0.157 | r= -0.327 | r= -0.091 | r_s_= -0.299 | r_s_= -0.146 | r= 0.127 |
| **⩒**O_2peak_ | r_s_= -0.208 | r= 0.136 | r= 0.201 | r_s_= -0.011 | r_s_= -0.244 | r= -0.314 |
| PWC_peak_ | r_s_= -0.219 | r= -0.042 | r= 0.229 | r_s_= -0.149 | r_s_= -0.398* | r= -0.174 |
| PWC_LT_ | r_s_= -0.112 | r_s_= 0.145 | r_s_= 0.287 | r_s_= -0.150 | r_s_= -0.341 | r_s_= -0.100 |
| PWC_IAS_ | r_s_= -0.225 | r= 0.077 | r= 0.273 | r_s_= -0.137 | r_s_= -0.303 | r_s_= -0.056 |
| PWC_2mmol_ | r_s_= -0.186 | r= -0.028 | r= 0.252 | r_s_= -0.171 | r_s_= -0.266 | r= -0.054 |
| PWC_3mmol_ | r_s_= -0.228 | r= -0.031 | r= 0.280 | r_s_= -0.172 | r_s_= -0.252 | r= -0.068 |
| HR_peak_ | r_s_= 0.036 | r= 0.172 | r= 0.093 | r_s_= 0.230 | r_s_= -0.048 | r= -0.307 |
| HR_LT_ | r_s_= 0.072 | r= 0.253 | r= 0.026 | r_s_= -0.025 | r_s_= 0.058 | r= -0.042 |
| HR_IAS_ | r_s_= 0.016 | r= 0.269 | r= 0.106 | r_s_= -0.007 | r_s_= 0.093 | r= -0.062 |
| HR_2mmol_ | r_s_= -0.104 | r= 0.099 | r= 0.131 | r_s_= -0.053 | r_s_= 0.101 | r= 0.035 |
| HR_3mmol_ | r_s_= -0.090 | r= 0.131 | r= 0.181 | r_s_= -0.037 | r_s_= 0.147 | r= 0.005 |
| %Ref.Norm | r_s_= -0.022 | r= -0.078 | r= 0.048 | r_s_= -0.252 | r_s_= -0.263 | r= -0.027 |
| lactat_max_ | r_s_= 0.012 | r= -0.018 | r= -0.081 | r_s_= 0.072 | r_s_= -0.326 | r= -0.358* |
| **miR-145-5p** | | | | | | |
| Age | r_s_= -0.330 | r_s_= -0.422* | r=-0.066 | r_s_= -0.279 | r_s_= -0.397* | r= 0.123 |
| **⩒**O_2peak_ | r_s_= 0.092 | r_s_= -0.036 | r= 0.021 | r_s_= -0.115 | r_s_= -0.183 | r= -0.337 |
| PWC_peak_ | r_s_= 0.064 | r_s_= -0.192 | r= -0.057 | r_s_= -0.306 | r_s_= -0.407* | r= -0.177 |
| PWC_LT_ | r_s_= 0.131 | r_s_= 0.031 | r_s_= 0.044 | r_s_= -0.218 | r_s_= -0.333 | r_s_= -0.058 |
| PWC_IAS_ | r_s_= 0.024 | r_s_= -0.139 | r= 0.016 | r_s_= -0.241 | r_s_= -0.330 | r_s_= -0.030 |
| PWC_2mmol_ | r_s_= 0.092 | r_s_= -0.123 | r= 0.058 | r_s_= -0.269 | r_s_= -0.329 | r= -0.042 |
| PWC_3mmol_ | r_s_= 0.049 | r_s_= -0.143 | r= 0.041 | r_s_= -0.292 | r_s_= -0.350* | r= -0.083 |
| HR_peak_ | r_s_= 0.261 | r_s_= 0.185 | r= -0.059 | r_s_= 0.243 | r_s_= 0.095 | r= -0.350* |
| HR_LT_ | r_s_= 0.324 | r_s_= 0.339 | r= -0.026 | r_s_= 0.104 | r_s_= 0.269 | r= -0.081 |
| HR_IAS_ | r_s_= 0.241 | r_s_= 0.339* | r= 0.067 | r_s_= 0.124 | r_s_= 0.268 | r= -0.117 |
| HR_2mmol_ | r_s_= 0.140 | r_s_=0.134 | r= 0.091 | r_s_= 0.042 | r_s_= 0.216 | r= 0.012 |
| HR_3mmol_ | r_s_= 0.164 | r_s_= 0.179 | r= 0.122 | r_s_= 0.079 | r_s_= 0.260 | r= -0.062 |
| %Ref.Norm | r_s_= 0.149 | r_s_= 0.047 | r= 0.035 | r_s_= -0.203 | r_s_= -0.292 | r= -0.008 |
| lactat_max_ | r_s_= 0.176 | r_s_= -0.021 | r= -0.282 | r_s_= -0.053 | r_s_= -0.195 | r= -0.285 |
| **miR-146a-5p** | | | | | | |
| Age | r_s_= 0.220 | r_s_= 0.424* | r= 0.193 | r_s_= 0.138 | r_s_= 0.312 | r_s_= 0.360* |
| **⩒**O_2peak_ | r_s_= -0.162 | r_s_= -0.250 | r= -0.077 | r_s_= -0.073 | r_s_= -0.071 | r_s_= -0.070 |
| PWC_peak_ | r_s_= 0.007 | r_s_= 0.012 | r= 0.108 | r_s_= 0.064 | r_s_= -0.017 | r_s_= -0.090 |
| PWC_LT_ | r_s_= -0.198 | r_s_= 0.056 | r_s_= 0.345* | r_s_= 0.061 | r_s_= -0.076 | r_s_= -0.177 |
| PWC_IAS_ | r_s_= -0.130 | r_s_= 0.107 | r= 0.261 | r_s_= 0.077 | r_s_= -0.030 | r_s_= -0.135 |
| PWC_2mmol_ | r_s_= -0.025 | r_s_= 0.097 | r= 0.224 | r_s_= 0.049 | r_s_= 0.009 | r_s_= 0.001 |
| PWC_3mmol_ | r_s_= -0.014 | r_s_= 0.099 | r= 0.238 | r_s_= 0.086 | r_s_= 0.021 | r_s_= -0.040 |
| HR_peak_ | r_s_= -0.185 | r_s_= -0.319 | r= -0.086 | r_s_= -0.288 | r_s_= -0.478** | r_s_= -0.296 |
| HR_LT_ | r_s_= -0.243 | r_s_= -0.282 | r= -0.029 | r_s_= -0.199 | r_s_= -0.381* | r_s_= -0.274 |
| HR_IAS_ | r_s_= -0.221 | r_s_= -0.198 | r= 0.008 | r_s_= -0.246 | r_s_= -0.397* | r_s_= -0.232 |
| HR_2mmol_ | r_s_= -0.065 | r_s_= -0.042 | r= 0.029 | r_s_= -0.236 | r_s_= -0.335 | r_s_= -0.124 |
| HR_3mmol_ | r_s_= -0.032 | r_s_= -0.006 | r= 0.049 | r_s_= -0.256 | r_s_= -0.365* | r_s_= -0.135 |
| %Ref.Norm | r_s_= 0.144 | r_s_= 0.027 | r= -0.111 | r_s_= -0.108 | r_s_= -0.069 | r_s_= 0.057 |
| lactat_max_ | r_s_= -0.150 | r_s_= -0.161 | r= -0.123 | r_s_= -0.087 | r_s_= -0.073 | r_s_= -0.120 |
| **miR-148b-3p** | | | | | | |
| Age | r= 0.304 | r_s_= 0.279 | r= 0.095 | r= 0.515** | r_s_= 0.371* | r_s_= 0.063 |
| **⩒**O_2peak_ | r= -0.131 | r_s_= -0.021 | r= -0.056 | r= -0.156 | r_s_= 0.091 | r_s_= 0.135 |
| PWC_peak_ | r= 0.035 | r_s_= 0.004 | r= 0.005 | r= 0.178 | r_s_= 0.201 | r_s_= 0.117 |
| PWC_LT_ | r_s_= -0.046 | r_s_= -0.275 | r_s_= -0.118 | r_s_= 0.079 | r_s_= -0.040 | r_s_= -0.136 |
| PWC_IAS_ | r= 0.052 | r_s_= -0.104 | r=-0.105 | r_s_= 0.096 | r_s_= 0.032 | r_s_= -0.073 |
| PWC_2mmol_ | r= 0.136 | r_s_= -0.055 | r= -0.069 | r= 0.173 | r_s_= 0.054 | r_s_= -0.095 |
| PWC_3mmol_ | r= 0.136 | r_s_= -0.028 | r= -0.051 | r= 0.218 | r_s_= 0.107 | r_s_= -0.032 |
| HR_peak_ | r= -0.123 | r_s_= -0.235 | r= -0.206 | r= -0.244 | r_s_= -0.063 | r_s_= 0.178 |
| HR_LT_ | r= -0.172 | r_s_= -0.439** | r= -0.299 | r= -0.143 | r_s_= -0.327 | r_s_= -0.325 |
| HR_IAS_ | r= -0.085 | r_s_= -0.405* | r= -0.362* | r= -0.213 | r_s_= -0.348* | r_s_= -0.259 |
| HR_2mmol_ | r= -0.009 | r_s_= -0.154 | r= -0.310 | r= -0.164 | r_s_= -0.259 | r_s_= -0.225 |
| HR_3mmol_ | r= 0.009 | r_s_= -0.240 | r= -0.359* | r= -0.200 | r_s_= -0.287 | r_s_= -0.214 |
| %Ref.Norm | r= -0.218 | r_s_= -0.167 | r= -0.102 | r= 0.150 | r_s_= -0.032 | r_s_= -0.173 |
| lactat_max_ | r= -0.263 | r_s_= -0.115 | r= 0.078 | r= -0.122 | r_s_= 0.121 | r_s_= 0.189 |
| **miR-150-5p** | | | | | | |
| Age | r_s_= -0.059 | r_s_= 0.081 | r_s_= 0.129 | r_s_= -0.154 | r_s_= -0.272 | r_s_= -0.109 |
| **⩒**O_2peak_ | r_s_= -0.159 | r_s_= 0.055 | r_s_= 0.205 | r_s_= 0.049 | r_s_= 0.169 | r_s_= 0.183 |
| PWC_peak_ | r_s_= -0.100 | r_s_= 0.144 | r_s_= 0.277 | r_s_= -0.139 | r_s_= 0.015 | r_s_= 0.191 |
| PWC_LT_ | r_s_= -0.073 | r_s_= 0.343* | r_s_= 0.487** | r_s_= -0.083 | r_s_= 0.021 | r_s_= 0.191 |
| PWC_IAS_ | r_s_= -0.105 | r_s_= 0.291 | r_s_= 0.436* | r_s_= -0.107 | r_s_= -0.043 | r_s_= 0.159 |
| PWC_2mmol_ | r_s_= -0.078 | r_s_= 0.163 | r_s_= 0.242 | r_s_= -0.087 | r_s_= -0.013 | r_s_= 0.162 |
| PWC_3mmol_ | r_s_= -0.099 | r_s_= 0.192 | r_s_= 0.296 | r_s_= -0.117 | r_s_= -0.039 | r_s_= 0.154 |
| HR_peak_ | r_s_= -0.243 | r_s_= -0.035 | r_s_= 0.122 | r_s_= -0.053 | r_s_= 0.103 | r_s_= 0.117 |
| HR_LT_ | r_s_= -0.038 | r_s_= 0.075 | r_s_= 0.058 | r_s_= 0.059 | r_s_= 0.144 | r_s_= 0.176 |
| HR_IAS_ | r_s_= -0.066 | r_s_= 0.118 | r_s_= 0.112 | r_s_= -0.004 | r_s_= 0.140 | r_s_= 0.190 |
| HR_2mmol_ | r_s_= 0.012 | r_s_= 0.194 | r_s_= 0.094 | r_s_= 0.046 | r_s_= 0.116 | r_s_= 0.127 |
| HR_3mmol_ | r_s_= -0.025 | r_s_= 0.224 | r_s_= 0.130 | r_s_= 0.011 | r_s_= 0.129 | r_s_= 0.138 |
| %Ref.Norm | r_s_= 0.115 | r_s_= 0.271 | r_s_= 0.052 | r_s_= -0.125 | r_s_= 0.067 | r_s_= 0.261 |
| lactat_max_ | r_s_= 0.030 | r_s_= 0.126 | r_s_= 0.110 | r_s_= 0.066 | r_s_= 0.172 | r_s_= 0.059 |
| **miR-200b-3p** | | | | | | |
| Age | r_s_= -0.432** | r_s_= -0.366* | r_s_= -0.073 | r= -0.160 | r_s_= -0.244 | r_s_= -0.192 |
| **⩒**O_2peak_ | r_s_= -0.211 | r_s_= -0.047 | r_s_= 0.083 | r= 0.193 | r_s_= -0.023 | r_s_= -0.112 |
| PWC_peak_ | r_s_= -0.037 | r_s_= -0.016 | r_s_= -0.046 | r= 0.110 | r_s_= -0.051 | r_s_= -0.159 |
| PWC_LT_ | r_s_= -0.013 | r_s_= 0.023 | r_s_= -0.040 | r_s_= 0.168 | r_s_= 0.147 | r_s_= 0.007 |
| PWC_IAS_ | r_s_= -0.043 | r_s_= -0.039 | r_s_= -0.060 | r_s_= 0.152 | r_s_= 0.069 | r_s_= -0.047 |
| PWC_2mmol_ | r_s_= -0.126 | r_s_= -0.055 | r_s_= -0.015 | r= 0.116 | r_s_= 0.001 | r_s_= -0.101 |
| PWC_3mmol_ | r_s_= -0.118 | r_s_= -0.028 | r_s_= 0.018 | r= 0.137 | r_s_= -0.008 | r_s_= -0.119 |
| HR_peak_ | r_s_= 0.000 | r_s_= 0.115 | r_s_= 0.146 | r= 0.097 | r_s_= -0.017 | r_s_= -0.130 |
| HR_LT_ | r_s_= 0.248 | r_s_= 0.174 | r_s_= -0.024 | r= 0.267 | r_s_= 0.250 | r_s_= 0.114 |
| HR_IAS_ | r_s_= 0.171 | r_s_= 0.145 | r_s_= 0.032 | r= 0.244 | r_s_= 0.248 | r_s_= 0.094 |
| HR_2mmol_ | r_s_= 0.019 | r_s_= -0.030 | r_s_=-0.062 | r= 0.137 | r_s_= 0.095 | r_s_= -0.011 |
| HR_3mmol_ | r_s_= 0.079 | r_s_= 0.095 | r_s_= 0.031 | r= 0.169 | r_s_= 0.142 | r_s_= 0.026 |
| %Ref.Norm | r_s_= -0.002 | r_s_= 0.043 | r_s_= 0.056 | r= -0.035 | r_s_= 0.018 | r_s_= -0.109 |
| lactat_max_ | r_s_= -0.041 | r_s_= -0.123 | r_s_= -0.090 | r= -0.044 | r_s_= -0.200 | r_s_= -0.181 |
| **miR-21-3p** | | | | | | |
| Age | r_s_= 0.192 | r= 0.317 | r_s_= 0.155 | r= 0.104 | r_s_= 0.091 | r= -0.069 |
| **⩒**O_2peak_ | r_s_= -0.108 | r= 0.004 | r_s_= 0.052 | r= -0.155 | r_s_= 0.058 | r= 0.216 |
| PWC_peak_ | r_s_= -0.131 | r= 0.217 | r_s_= 0.284 | r= 0.053 | r_s_= 0.137 | r= 0.056 |
| PWC_LT_ | r_s_= -0.102 | r_s_= 0.304 | r_s_= 0.275 | r_s_= 0.162 | r_s_= 0.278 | r_s_= 0.033 |
| PWC_IAS_ | r_s_= -0.099 | r= 0.289 | r_s_= 0.301 | r_s_= 0.154 | r_s_= 0.267 | r_s_= 0.023 |
| PWC_2mmol_ | r_s_= -0.093 | r= 0.348* | r_s_= 0.266 | r= 0.228 | r_s_= 0.301 | r= -0.001 |
| PWC_3mmol_ | r_s_= -0.072 | r= 0.349* | r_s_= 0.274 | r= 0.200 | r_s_= 0.296 | r= -0.005 |
| HR_peak_ | r_s_= -0.157 | r= -0.335 | r_s_= -0.152 | r= 0.242 | r_s_= 0.196 | r= -0.109 |
| HR_LT_ | r_s_= -0.120 | r= -0.252 | r_s_= -0.120 | r= 0.280 | r_s_= 0.227 | r= -0.161 |
| HR_IAS_ | r_s_= -0.114 | r= -0.221 | r_s_= -0.107 | r= 0.312 | r_s_= 0.328 | r= -0.131 |
| HR_2mmol_ | r_s_= -0.020 | r= -0.070 | r_s_= -0.067 | r= 0.361* | r_s_= 0.265 | r= -0.159 |
| HR_3mmol_ | r_s_= -0.071 | r= -0.105 | r_s_= -0.086 | r= 0.369* | r_s_= 0.308 | r= -0.153 |
| %Ref.Norm | r_s_= 0.136 | r= 0.212 | r_s_= 0.161 | r= 0.116 | r_s_= 0.467** | r= 0.212 |
| lactat_max_ | r_s_= -0.068 | r= -0.233 | r_s_= -0.024 | r= -0.176 | r_s_= -0.117 | r= 0.070 |
| **miR-222-3p** | | | | | | |
| Age | r_s_= 0.146 | r= 0.028 | r= -0.084 | r= 0.299 | r= 0.320 | r= 0.044 |
| **⩒**O_2peak_ | r_s_= -0.040 | r= -0.076 | r= -0.005 | r= -0.106 | r= -0.277 | r= -0.148 |
| PWC_peak_ | r_s_= 0.200 | r= -0.081 | r= -0.168 | r= 0.111 | r= -0.070 | r= -0.139 |
| PWC_LT_ | r_s_= 0.024 | r_s_= 0.019 | r_s_= -0.097 | r_s_= 0.175 | r_s_= 0.065 | r_s_= -0.075 |
| PWC_IAS_ | r_s_= 0.112 | r= -0.022 | r= -0.075 | r_s_= 0.150 | r_s_= 0.064 | r_s_= -0.057 |
| PWC_2mmol_ | r_s_= 0.124 | r= -0.136 | r= -0.208 | r= 0.157 | r= 0.038 | r= -0.100 |
| PWC_3mmol_ | r_s_= 0.145 | r= -0.094 | r= -0.182 | r= 0.186 | r= 0.051 | r= -0.108 |
| HR_peak_ | r_s_= -0.107 | r= -0.089 | r= 0.017 | r= -0.112 | r= -0.380* | r= -0.213 |
| HR_LT_ | r_s_= -0.085 | r= -0.139 | r= -0.010 | r= -0.072 | r= -0.131 | r= -0.047 |
| HR_IAS_ | r_s_= -0.094 | r= -0.114 | r= 0.005 | r= -0.106 | r= -0.151 | r= -0.030 |
| HR_2mmol_ | r_s_= 0.021 | r= -0.336 | r= -0.246 | r= -0.113 | r= -0.161 | r= -0.038 |
| HR_3mmol_ | r_s_= -0.036 | r= -0.265 | r= -0.179 | r= -0.128 | r= -0.171 | r= -0.028 |
| %Ref.Norm | r_s_= -0.043 | r= -0.274 | r= -0.209 | r= 0.217 | r= -0.084 | r= -0.253 |
| lactat_max_ | r_s_= -0.142 | r= -0.239 | r= -0.141 | r= -0.128 | r= -0.385* | r= -0.209 |
| **miR-223-3p** | | | | | | |
| Age | r_s_= -0.401* | r_s_= -0.020 | r= 0.257 | r_s_= 0.077 | r_s_= -0.225 | r_s_= -0.244 |
| **⩒**O_2peak_ | r_s_= -0.201 | r_s_= 0.139 | r= 0.335 | r_s_= -0.009 | r_s_= -0.235 | r_s_= -0.031 |
| PWC_peak_ | r_s_= -0.325 | r_s_= 0.034 | r= 0.269 | r_s_= 0.002 | r_s_= -0.234 | r_s_= -0.076 |
| PWC_LT_ | r_s_= -0.321 | r_s_= -0.087 | r_s_= 0.285 | r_s_= -0.072 | r_s_= -0.420* | r_s_= -0.150 |
| PWC_IAS_ | r_s_= -0.371* | r_s_= -0.130 | r= 0.215 | r_s_= -0.094 | r_s_= -0.412* | r_s_= -0.145 |
| PWC_2mmol_ | r_s_= -0.382* | r_s_= -0.180 | r= 0.166 | r_s_= -0.134 | r_s_= -0.374* | r_s_= -0.097 |
| PWC_3mmol_ | r_s_= -0.384* | r_s_= -0.166 | r= 0.179 | r_s_= -0.094 | r_s_= -0.388* | r_s_= -0.137 |
| HR_peak_ | r_s_= 0.096 | r_s_= 0.128 | r= -0.001 | r_s_= -0.128 | r_s_= 0.090 | r_s_= 0.293 |
| HR_LT_ | r_s_= 0.217 | r_s_= 0.037 | r= -0.115 | r_s_= -0.193 | r_s_= -0.082 | r_s_= 0.161 |
| HR_IAS_ | r_s_= 0.198 | r_s_= -0.024 | r= -0.197 | r_s_= -0.213 | r_s_= -0.121 | r_s_= 0.163 |
| HR_2mmol_ | r_s_= 0.033 | r_s_= -0.020 | r= -0.143 | r_s_= -0.162 | r_s_= -0.049 | r_s_= 0.155 |
| HR_3mmol_ | r_s_= 0.069 | r_s_= -0.117 | r= -0.213 | r_s_= -0.163 | r_s_= -0.062 | r_s_= 0.148 |
| %Ref.Norm | r_s_= -0.448** | r_s_= -0.214 | r= 0.161 | r_s_= -0.210 | r_s_= -0.299 | r_s_= 0.041 |
| lactat_max_ | r_s_= -0.074 | r_s_= 0.399* | r= 0.378* | r_s_= 0.097 | r_s_= -0.038 | r_s_= -0.027 |
| **miR-23a-3p** | | | | | | |
| Age | r_s_= 0.046 | r= -0.026 | r_s_= -0.028 | r= 0.245 | r= 0.086 | r= -0.115 |
| **⩒**O_2peak_ | r_s_= -0.135 | r= -0.143 | r_s_= 0.124 | r= -0.132 | r= -0.283 | r= -0.234 |
| PWC_peak_ | r_s_= 0.070 | r= -0.153 | r_s_= -0.044 | r= 0.078 | r= -0.301 | r= -0.447** |
| PWC_LT_ | r_s_= -0.126 | r_s_= 0.060 | r_s_= 0.230 | r_s_= 0.083 | r_s_= -0.180 | r_s_= -0.292 |
| PWC_IAS_ | r_s_= 0.004 | r= -0.057 | r_s_= 0.077 | r_s_= 0.092 | r_s_= -0.196 | r_s_= -0.328 |
| PWC_2mmol_ | r_s_= 0.028 | r= -0.098 | r_s_= 0.071 | r= 0.046 | r= -0.236 | r= -0.336 |
| PWC_3mmol_ | r_s_= 0.050 | r= -0.100 | r_s_= 0.057 | r= 0.091 | r= -0.230 | r= -0.370* |
| HR_peak_ | r_s_= -0.188 | r= -0.188 | r_s_= 0.008 | r= -0.329 | r= -0.341 | r= -0.122 |
| HR_LT_ | r_s_= -0.248 | r= -0.069 | r_s_= 0.106 | r= -0.227 | r= -0.091 | r= 0.092 |
| HR_IAS_ | r_s_= -0.232 | r= -0.016 | r_s_= 0.149 | r= -0.292 | r= -0.133 | r= 0.100 |
| HR_2mmol_ | r_s_= -0.139 | r= -0.158 | r_s_= 0.024 | r= -0.323 | r= -0.193 | r= 0.052 |
| HR_3mmol_ | r_s_= -0.093 | r= -0.093 | r_s_= 0.038 | r= -0.357* | r= -0.189 | r= 0.088 |
| %Ref.Norm | r_s_= -0.188 | r= -0.271 | r_s_= 0.114 | r= -0.138 | r= -0.228 | r= -0.160 |
| lactat_max_ | r_s_= -0.311 | r= -0.336 | r_s_= 0.026 | r= -0.143 | r= -0.383* | r= -0.347* |
| **miR-29a-3p** | | | | | | |
| Age | r= 0.047 | r= 0.165 | r= 0.122 | r= 0.131 | r= 0.216 | r= 0.073 |
| **⩒**O_2peak_ | r= -0.135 | r= -0.253 | r= -0.141 | r= -0.298 | r= -0.547** | r= -0.193 |
| PWC_peak_ | r= -0.096 | r= -0.174 | r=-0.095 | r= -0.145 | r= -0.436* | r= -0.248 |
| PWC_LT_ | r_s_= -0.359* | r_s_= -0.022 | r_s_= 0.205 | r_s_= -0.326 | r_s_= -0.328 | r_s_= -0.049 |
| PWC_IAS_ | r= -0.266 | r= -0.117 | r= 0.084 | r_s_= -0.308 | r_s_= -0.337 | r_s_= -0.065 |
| PWC_2mmol_ | r= -0.105 | r= -0.088 | r= -0.006 | r= -0.218 | r= -0.212 | r= 0.038 |
| PWC_3mmol_ | r= -0.127 | r= -0.107 | r= -0.009 | r= -0.206 | r= -0.258 | r= -0.018 |
| HR_peak_ | r= -0.217 | r= -0.222 | r= -0.051 | r= -0.115 | r= -0.327 | r= -0.165 |
| HR_LT_ | r= -0.326 | r= -0.143 | r= 0.103 | r= -0.294 | r= -0.224 | r= 0.118 |
| HR_IAS_ | r= -0.257 | r= -0.069 | r= 0.122 | r= -0.314 | r= -0.201 | r= 0.169 |
| HR_2mmol_ | r= -0.116 | r= -0.099 | r= -0.009 | r= -0.267 | r= -0.106 | r= 0.203 |
| HR_3mmol_ | r= -0.109 | r=-0.040 | r= 0.042 | r= -0.281 | r= -0.115 | r= 0.216 |
| %Ref.Norm | r= -0.045 | r= 0.066 | r= 0.095 | r= -0.338 | r= -0.204 | r= 0.186 |
| lactat_max_ | r= -0.049 | r= -0.174 | r= -0.129 | r= -0.100 | r= -0.543** | r= -0.397* |
| **miR-30a-5p** | | | | | | |
| Age | r= 0.315 | r= 0.156 | r= -0.199 | r= 0.190 | r_s_= 0.271 | r= 0.071 |
| **⩒**O_2peak_ | r= -0.243 | r= -0.291 | r= -0.064 | r= -0.137 | r_s_= -0.151 | r= -0.128 |
| PWC_peak_ | r= 0.037 | r= -0.148 | r= -0.234 | r= 0.052 | r_s_= -0.166 | r= -0.241 |
| PWC_LT_ | r_s_= -0.086 | r_s_= -0.073 | r_s_= 0.037 | r_s_= -0.046 | r_s_= -0.275 | r_s_= -0.187 |
| PWC_IAS_ | r= -0.031 | r= -0.099 | r= -0.086 | r_s_= -0.002 | r_s_= -0.240 | r_s_= -0.207 |
| PWC_2mmol_ | r= 0.095 | r= 0.011 | r= -0.107 | r= -0.076 | r_s_= -0.200 | r= -0.115 |
| PWC_3mmol_ | r= 0.091 | r= -0.019 | r= -0.138 | r= -0.024 | r_s_= -0.184 | r= -0.175 |
| HR_peak_ | r= -0.288 | r= -0.231 | r= 0.069 | r= -0.085 | r_s_= -0.337 | r= -0.250 |
| HR_LT_ | r= -0.387* | r= -0.219 | r= 0.209 | r= -0.249 | r_s_= -0.438* | r= -0.167 |
| HR_IAS_ | r= -0.330 | r= -0.143 | r= 0.234 | r= -0.288 | r_s_= -0.493** | r= -0.154 |
| HR_2mmol_ | r= -0.188 | r= -0.051 | r= 0.171 | r= -0.317 | r_s_= -0.430 * | r= -0.022 |
| HR_3mmol_ | r= -0.190 | r= -0.036 | r= 0.193 | r= -0.331 | r_s_= -0.468** | r= -0.050 |
| %Ref.Norm | r= -0.288 | r= -0.245 | r= 0.051 | r= -0.040 | r_s_= -0.184 | r= -0.217 |
| lactat_max_ | r= -0.244 | r= -0.210 | r= 0.040 | r= -0.028 | r_s_= -0.142 | r= -0.137 |
| **miR-338-3p** | | | | | | |
| Age | r_s_= -0.201 | r_s_= 0.022 | r= 0.246 | r_s_= -0.077 | r_s_= -0.054 | r= 0.092 |
| **⩒**O_2peak_ | r_s_= -0.191 | r_s_= -0.006 | r= 0.129 | r_s_= -0.104 | r_s_= -0.209 | r= -0.065 |
| PWC_peak_ | r_s_= -0.174 | r_s_= 0.016 | r= 0.150 | r_s_= -0.034 | r_s_= -0.099 | r= -0.022 |
| PWC_LT_ | r_s_= -0.016 | r_s_= 0.015 | r_s_= 0.076 | r_s_= -0.181 | r_s_= -0.170 | r_s_= 0.043 |
| PWC_IAS_ | r_s_= -0.112 | r_s_= -0.081 | r= 0.056 | r_s_= -0.195 | r_s_= -0.170 | r_s_= 0.066 |
| PWC_2mmol_ | r_s_= -0.178 | r_s_= -0.028 | r= 0.118 | r_s_= -0.202 | r_s_= -0.172 | r= 0.061 |
| PWC_3mmol_ | r_s_= -0.201 | r_s_= -0.069 | r= 0.085 | r_s_= -0.171 | r_s_= -0.140 | r= 0.088 |
| HR_peak_ | r_s_= 0.279 | r_s_= 0.051 | r= -0.240 | r_s_= 0.134 | r_s_= 0.144 | r= -0.061 |
| HR_LT_ | r_s_= 0.338 | r_s_= 0.063 | r= -0.137 | r_s_= -0.073 | r_s_= -0.033 | r= 0.098 |
| HR_IAS_ | r_s_= 0.312 | r_s_= -0.034 | r= -0.254 | r_s_= -0.067 | r_s_= 0.017 | r= 0.087 |
| HR_2mmol_ | r_s_= 0.173 | r_s_= -0.018 | r= -0.083 | r_s_= -0.027 | r_s_= 0.002 | r= 0.012 |
| HR_3mmol_ | r_s_= 0.198 | r_s_= -0.099 | r= -0.209 | r_s_= -0.009 | r_s_= 0.062 | r= 0.043 |
| %Ref.Norm | r_s_= 0.026 | r_s_= 0.171 | r= 0.199 | r_s_= -0.224 | r_s_= -0.161 | r= 0.133 |
| lactat_max_ | r_s_= 0.221 | r_s_= 0.329 | r= 0.156 | r_s_= 0.199 | r_s_= -0.195 | r= -0.374* |
| **miR-652-3p** | | | | | | |
| Age | r= 0.047 | r= -0.007 | r= -0.034 | r_s_= 0.202 | r_s_= 0.006 | r= -0.249 |
| **⩒**O_2peak_ | r= 0.107 | r= 0.117 | r= 0.050 | r_s_= 0.046 | r_s_= 0.391* | r= 0.308 |
| PWC_peak_ | r= -0.032 | r= 0.143 | r= 0.156 | r_s_= 0.232 | r_s_= 0.357* | r= 0.088 |
| PWC_LT_ | r_s_= 0.143 | r_s_= 0.046 | r_s_= -0.003 | r_s_= 0.241 | r_s_= 0.179 | r_s_= 0.008 |
| PWC_IAS_ | r= 0.106 | r= 0.102 | r= 0.036 | r_s_= 0.227 | r_s_= 0.208 | r_s_= 0.043 |
| PWC_2mmol_ | r= -0.034 | r= 0.141 | r= 0.155 | r_s_= 0.260 | r_s_= 0.206 | r= -0.048 |
| PWC_3mmol_ | r= -0.026 | r= 0.142 | r= 0.151 | r_s_= 0.241 | r_s_= 0.222 | r= -0.035 |
| HR_peak_ | r= 0.291 | r= 0.046 | r= -0.125 | r_s_= 0.036 | r_s_= 0.328 | r= 0.291 |
| HR_LT_ | r= 0.437* | r= -0.077 | r= -0.327 | r_s_= 0.096 | r_s_= 0.180 | r= 0.204 |
| HR_IAS_ | r= 0.419* | r= -0.112 | r= -0.351* | r_s_= 0.060 | r_s_= 0.192 | r= 0.200 |
| HR_2mmol_ | r= 0.277 | r= -0.006 | r= -0.167 | r_s_= 0.241 | r_s_=0.227 | r= 0.189 |
| HR_3mmol_ | r= 0.280 | r= -0.064 | r= -0.224 | r_s_= 0.062 | r_s_= 0.215 | r= 0.199 |
| %Ref.Norm | r= 0.018 | r= 0.082 | r= 0.068 | r_s_= 0.302 | r_s_= 0.271 | r= -0.006 |
| lactat_max_ | r= 0.140 | r= 0.162 | r= 0.074 | r_s_= -0.047 | r_s_= 0.423* | r= 0.379* |

*p<0.05; **p<0.01; ***p<0.001. Abbreviations: resting = pre-exercise measurements, post CPET = post exercise measurements; Δ= differences between pre and post exercise measurements; base =baseline examination; end = end of study examination; CPET= cardiopulmonary exercise test; r= Pearson’s Correlation coefficient, r_s_= Spearman-Rho Correlation coefficient, **⩒**O_2peak_ = peak oxygen uptake; PWC= physical work capacity in watt, LT= lactate threshold, IAS= Individual anaerobic threshold, 2mmol= lactate threshold of 2mmol per liter; 3mmol= lactate threshold of 3mmol per liter; HR= heart rate; %Ref.Norm= percent based on reference performance norm
