# Supplementary material for: Effects of structured exercise training on miRNA expression in previously sedentary individuals
Source: PLoS One. 2024 Dec 18;19(12):e0314281. doi: 10.1371/journal.pone.0314281 (PMC11654927; doi:10.1371/journal.pone.0314281)
Supplement: S2 Table — KEGG pathway analysis using DIANA-miRPath v4.0, with a false discovery rate of <0.05. Depicted are the numbers of genes within the pathway, as well as numbers of target genes within the pathways, by miR-107, miR-148b-3p; miR-143-3p; miR-145-5p; miR-21-3p and miR-29a-3p. (DOCX) [file pone.0314281.s008.docx]

**S2 Table. KEGG pathway analysis for the five miRNAs, which significant changes in the exercise response only after 4 months of increased exercise.**

| **KEGG pathway name** | **# pathway genes** | **# target genes** |
| --- | --- | --- |
| Proteoglycans in cancer | 220 | 104 |
| Protein processing in endoplasmic reticulum | 194 | 93 |
| Autophagy - animal | 146 | 75 |
| Shigellosis | 268 | 117 |
| Salmonella infection | 277 | 117 |
| Ubiquitin mediated proteolysis | 142 | 71 |
| Cell cycle | 129 | 65 |
| p53 signaling pathway | 75 | 43 |
| FoxO signaling pathway | 139 | 66 |
| Pathways in cancer | 555 | 194 |
| Focal adhesion | 213 | 88 |
| Endocytosis | 311 | 118 |
| Amyotrophic lateral sclerosis | 408 | 147 |
| Renal cell carcinoma | 70 | 38 |
| Small cell lung cancer | 100 | 49 |
| Neurotrophin signaling pathway | 124 | 57 |
| Colorectal cancer | 88 | 44 |
| Fluid shear stress and atherosclerosis | 149 | 65 |
| Pancreatic cancer | 78 | 40 |
| Pathogenic Escherichia coli infection | 222 | 88 |
| Prostate cancer | 101 | 48 |
| Mitophagy - animal | 76 | 39 |
| Chronic myeloid leukemia | 79 | 40 |
| Alzheimer disease | 426 | 149 |
| Regulation of actin cytoskeleton | 224 | 88 |
| MAPK signaling pathway | 329 | 120 |
| Longevity regulating pathway | 105 | 49 |
| Viral carcinogenesis | 265 | 100 |
| Oocyte meiosis | 134 | 58 |
| Bacterial invasion of epithelial cells | 80 | 39 |
| AGE-RAGE signaling pathway in diabetic complications | 115 | 51 |
| Hippo signaling pathway | 164 | 67 |
| Pathways of neurodegeneration - multiple diseases | 539 | 178 |
| Cellular senescence | 219 | 84 |
| Adherens junction | 79 | 38 |
| AMPK signaling pathway | 130 | 55 |
| HIF-1 signaling pathway | 112 | 49 |
| Progesterone-mediated oocyte maturation | 104 | 46 |
| Spinocerebellar ataxia | 145 | 59 |
| Glioma | 79 | 37 |
| PI3K-Akt signaling pathway | 372 | 127 |
| Rap1 signaling pathway | 214 | 80 |
| Thyroid hormone signaling pathway | 137 | 56 |
| Apoptosis | 151 | 60 |
| Hepatitis B | 177 | 68 |
| mTOR signaling pathway | 177 | 68 |
| EGFR tyrosine kinase inhibitor resistance | 82 | 37 |
| Endocrine resistance | 118 | 49 |
| Human papillomavirus infection | 406 | 135 |
| Signaling pathways regulating pluripotency of stem cells | 156 | 61 |
| Hedgehog signaling pathway | 59 | 29 |
| Carbon metabolism | 122 | 50 |
| Hepatocellular carcinoma | 177 | 67 |
| Phosphatidylinositol signaling system | 101 | 43 |
| Growth hormone synthesis, secretion and action | 129 | 52 |
| Axon guidance | 186 | 69 |
| Yersinia infection | 147 | 57 |
| Cysteine and methionine metabolism | 53 | 26 |
| Measles | 161 | 61 |
| Kaposi sarcoma-associated herpesvirus infection | 245 | 86 |
| Insulin signaling pathway | 153 | 58 |
| Apelin signaling pathway | 140 | 54 |
| Huntington disease | 339 | 112 |
| Longevity regulating pathway - multiple species | 79 | 34 |
| Tight junction | 182 | 66 |
| Vasopressin-regulated water reabsorption | 47 | 23 |
| RNA transport | 199 | 71 |
| Sphingolipid signaling pathway | 133 | 51 |
| ErbB signaling pathway | 86 | 36 |
| Insulin resistance | 124 | 48 |
| Central carbon metabolism in cancer | 74 | 32 |
| TNF signaling pathway | 131 | 50 |
| Relaxin signaling pathway | 138 | 52 |
| Non-small cell lung cancer | 81 | 34 |
| Vibrio cholerae infection | 60 | 27 |
| Lysine degradation | 69 | 30 |
| Chagas disease | 116 | 45 |
| Apoptosis - multiple species | 32 | 17 |
| Hepatitis C | 173 | 62 |
| Melanoma | 76 | 32 |
| Endometrial cancer | 61 | 27 |
| Human immunodeficiency virus 1 infection | 277 | 92 |
| Alcoholism | 195 | 68 |
| Parkinson disease | 282 | 93 |
| Biosynthesis of amino acids | 78 | 32 |
| Platinum drug resistance | 75 | 31 |
| Breast cancer | 163 | 58 |
| N-Glycan biosynthesis | 51 | 23 |
| Selenocompound metabolism | 18 | 11 |
| Oxytocin signaling pathway | 161 | 57 |
| Prolactin signaling pathway | 73 | 30 |
| Bladder cancer | 43 | 20 |
| VEGF signaling pathway | 61 | 26 |
| Gap junction | 99 | 38 |
| Platelet activation | 136 | 49 |
| Neutrophil extracellular trap formation | 205 | 69 |
| Inositol phosphate metabolism | 78 | 31 |
| Transcriptional misregulation in cancer | 206 | 69 |
| PD-L1 expression and PD-1 checkpoint pathway in cancer | 101 | 38 |
| Epithelial cell signaling in Helicobacter pylori infection | 79 | 31 |
| Circadian rhythm | 31 | 15 |
| Prion disease | 331 | 103 |
| Aldosterone-regulated sodium reabsorption | 37 | 17 |
| Long-term potentiation | 71 | 28 |
| Gastric cancer | 162 | 55 |
| Valine, leucine and isoleucine degradation | 50 | 21 |
| Acute myeloid leukemia | 69 | 27 |
| C-type lectin receptor signaling pathway | 116 | 41 |
| MicroRNAs in cancer | 334 | 102 |
| Hippo signaling pathway - multiple species | 30 | 14 |
| Autophagy - other | 33 | 15 |
| Regulation of lipolysis in adipocytes | 61 | 24 |
| 2-Oxocarboxylic acid metabolism | 19 | 10 |
| Metabolic pathways | 1634 | 440 |
| Fc gamma R-mediated phagocytosis | 101 | 36 |
| Steroid biosynthesis | 22 | 11 |
| Terpenoid backbone biosynthesis | 22 | 11 |
| cGMP-PKG signaling pathway | 175 | 57 |
| Citrate cycle (TCA cycle) | 31 | 14 |
| Ras signaling pathway | 241 | 75 |
| TGF-beta signaling pathway | 103 | 36 |
| Wnt signaling pathway | 173 | 56 |
| Lysosome | 145 | 48 |
| Type II diabetes mellitus | 57 | 22 |
| Aminoacyl-tRNA biosynthesis | 57 | 22 |
| Alanine, aspartate and glutamate metabolism | 38 | 16 |
| Necroptosis | 174 | 56 |
| Estrogen signaling pathway | 167 | 54 |
| mRNA surveillance pathway | 108 | 37 |
| T cell receptor signaling pathway | 115 | 39 |
| Human cytomegalovirus infection | 306 | 92 |
| Dopaminergic synapse | 143 | 47 |
| Phospholipase D signaling pathway | 158 | 51 |
| Cushing syndrome | 176 | 56 |
| Propanoate metabolism | 36 | 15 |
| Leukocyte transendothelial migration | 120 | 40 |

KEGG pathway analysis using DIANA-miRPath v4.0, with a false discovery rate of <0.05. Depicted are the numbers of genes within the pathway, as well as numbers of target genes within the pathways, by miR-107, miR-148b-3p; miR-143-3p; miR-145-5p; miR-21-3p and miR-29a-3p.
