# Supplementary material for: Effects of structured exercise training on miRNA expression in previously sedentary individuals
Source: PLoS One. 2024 Dec 18;19(12):e0314281. doi: 10.1371/journal.pone.0314281 (PMC11654927; doi:10.1371/journal.pone.0314281)
Supplement: S1 File — (PDF) [file pone.0314281.s009.pdf]

STUDIENPROTOKOLL

TITEL DER STUDIE

Einfluss einer 4-monatigen Trainingsintervention auf Belastungs-induzierte Veränderungen epigenetischer Marker der Herzgesundheit in gesunden, körperlich inaktiven Personen

AKRONYM

miRNA-4-Heart

VERSIONSNUMMER DES PROTOKOLLS UND DATUM

Version 1.9 - 03.02.2021

STUDIENREFERENZNUMMER

**Bescheidnummer der Ethikkommission:**	1207/2020
**Nummer des Sponsors:**	P-20/01/008-NIB
**Studienregistrierungsnummer:**	NCT04791306

KONTAKTE

**Studienleiterin**	Dr.rer.nat Barbara Mayr, BSc, MSc<br><br>Universitätsinstitut für präventive und rehabilitative Sportmedizin<br>Lindhofstraße 20<br>5020 Salzburg<br>Österreich
**Sponsor**	**Universitätsinstitut für präventive und rehabilitative Sportmedizin, Uniklinikum Salzburg**<br><br>Vertreter des Sponsors:<br>Univ.- Prof. Dr. Dr. Josef Niebauer, MBA (Primar)<br>Universitätsinstitut für präventive und rehabilitative Sportmedizin<br>Lindhofstraße 20<br>5020 Salzburg<br><br>und<br><br>Ludwig Boltzmann Institut für digitale Gesundheit und Prävention<br>5020 Salzburg<br>Österreich<br><br><br>**Paracelsus Medizinische Privatuniversität**<br><br>Kontakt zur Forschungsförderung Prosperamus!:<br>Mag. Dorothea Kölblinger, MAS,<br>Strubergasse 21<br>5020 Salzburg<br>Österreich

**Mitwirkende am Studienprotokoll**	Priv.-Doz. Dr. Dr. med. Mahdi Sareban<br><br>Universitätsinstitut für präventive und rehabilitative Sportmedizin<br>Lindhofstraße 20<br>5020 Salzburg<br>Österreich<br><br>und<br><br>Ludwig Boltzmann Institut für digitale Gesundheit und Prävention<br>Lindhofstraße 22<br>5020 Salzburg<br>Österreich<br><br>Michael Neudorfer, M.Sc., M.Ed.<br><br>Universitätsinstitut für präventive und rehabilitative Sportmedizin<br>Lindhofstraße 20<br>5020 Salzburg<br>Österreich<br><br>Mag. Dr. Daniela Wurhofer, Bakk. techn.<br><br>Ludwig Boltzmann Institut für digitale Gesundheit und Prävention<br>Lindhofstraße 22<br>5020 Salzburg<br>Österreich<br><br>Dr. Georg Zimmermann<br><br>Team Biostatistics and Big Medical Data, IDA Lab Salzburg<br>Forschungsmanagement & Technologietransfer, Paracelsus Medizinische Privatuniversität Salzburg<br>Strubergasse 16<br>A-5020 Salzburg

Inhalt

KONTAKTE 2
1. **Hintergrund und Überblick** 4
1.1. ***Primäre Studienhypothese*** 6
1.2. ***Sekundäre Studienhypothesen*** 6
2. **Zeitraum** 6
3. **TeilnehmerInnen** 6
4. **Rekrutierung** 6
4.1. ***Einschlusskriterien*** 6
4.2. ***Ausschlusskriterien*** 6
5. **Ablauf** 7
6. **Untersuchungen** 7
6.1. ***Pulswellengeschwindigkeit (PWA)*** 8
6.2. ***Bioimpedanzanalyse (BIA)*** 9
6.3. ***Spiroergometrie*** 9
6.4. ***Epigenetische Untersuchung*** 10
6.5. ***Ausdauer- und Krafttraining*** 11
6.6. ***Aktivitätsplanungs- und Dokumentations-Tool „aktivplan App"*** 11
6.6.1. Anwendung der „*aktivplan*" App 11
6.6.2. Evaluierung der „*aktivplan*" App 12
7. **Fallzahlberechnung** 13
8. **Ethische Aspekte** 14
9. **Versicherung** 14
10. **Studienabbruch** 14
11. **Datenaufbewahrung** 15
12. **Auswertemethoden** 15
13. **Sicherheit** 15
14. **Datenschutz** 16
15. **Berichterstattung** 18
16. **Referenzen** 18

1. Hintergrund und Überblick

Herzkreislauferkrankungen stellen die häufigste Todesursache weltweit dar [1]. Neben der genetischen, nicht beeinflussbaren Prädisposition solcher Erkrankungen, gibt es weitere

*epi*genetische Risikomarker, welche modifizierbar sind [2]. Epigenetik bezieht sich dabei auf Veränderungen in der Expression von Genen, ohne dass die DNA Sequenz verändert wird. Mechanistisch werden diese Veränderungen unter anderem durch Microribonukleinsäuren (miRNAs) hervorgerufen. Die Menge von bestimmten miRNAs wurde dabei mit dem Risiko für einige Herzkreislauferkrankungen wie der Koronaren Herzerkrankung in Zusammenhang gebracht [3-6]. Diese epigenetischen Veränderungen können durch den Lebensstil und insbesondere durch körperliche Aktivität beeinflusst werden [2, 7]. In einer Querschnittstudie konnte unsere Arbeitsgruppe zeigen, dass sich die Antwort potentiell kardioprotektiver miRNAs auf eine einzelne Bewegungsintervention in Form einer Ergometrie zwischen PatientInnen mit einer Koronarer Herzerkrankung signifikant von einem gesunden Kollektiv unterscheidet [8]. Zudem konnte in einem gesunden Kollektiv gezeigt werden, dass auch sich wiederholende Trainingsreize einen Einfluss auf spezifische miRNAs haben. Dies lässt wiederum auf epigenetische Adaptationsmechanismen der Trainingsreize schließen [7]. Jedoch wurden in diesen longitudinalen Studien hauptsächlich junge, gesunde und körperlich aktive Männer untersucht. Insgesamt unterstreichen diese Daten die Rolle von miRNAs als möglicher Biomarker personalisierter Empfehlungen zur Lebensstilmodifikation für die Primärprävention von Herzkreislauferkrankungen. Die Notwendigkeit der Optimierung des Effektes von Empfehlungen zur körperlichen Aktivität zeigt sich dadurch, dass nur 20% der Bevölkerung in Industrieländer die WHO-Empfehlungen von 150 min/Woche moderater oder 75 min/Woche intensiver körperlicher Aktivität erreichen [9-11]. Eine Möglichkeit der Personalisierung bietet die Nutzung des epigenetischen Expressionsverhaltens auf akute Bewegungsinterventionen, aber auch die longitudinale Untersuchung dieser epigenetischen Veränderungen nach einer Leitlinien-gerechten Optimierung des Bewegungsverhaltens.

Zu diesem Zwecke soll in dieser Studie erforscht werden, wie sich das Expressionsmuster spezifischer miRNAs, die in der Literatur mit der Reduktion kardiovaskulärer Endpunkte in Zusammenhang gebracht wurden, auf eine einzelne akute Bewegungsintervention in einem gesunden aber körperlich inaktiven Kollektiv verändert. Zudem soll in dem Kollektiv diese Reaktion nach 4 Monaten mit regelmäßigem körperlichem Training (>150min/Woche) untersucht werden. Um zu gewährleisten, dass die Studienteilnehmer das gewünschte Aktivitätspensum erfüllen und damit auch der Intensitätsanteil beurteilt werden kann, absolvieren die ProbandInnen einmal pro Woche ein zentrumbasiertes und überwachtes Ausdauer- und Krafttraining und erhalten für das eigenständige heimbasierte Training Hilfestellung in Form einer digitalen Aktivitätsplanungs- und Dokumentations-App. Die entsprechenden Studienhypothesen lauten:

1.1. Primäre Studienhypothese

- Eine 4-monatige Trainingsintervention führt in einem gesunden, körperlich inaktiven Kollektiv zu einer Belastungs-induziert kardioprotektiven miRNA-Expressionsprofiländerung.

1.2. Sekundäre Studienhypothesen

- Die Belastungs-induzierte Veränderung kardioprotektiver miRNAs nach einer 4-monatigen Trainingsintervention korreliert mit Veränderungen kardiovaskulärer Risikofaktoren (Gewicht, Blutlipide, Gefäßsteifigkeit, körperliche Leistungsfähigkeit).
- Eine digitale App-gestützte Trainingsintervention wird von einem gesunden, körperlich inaktiven Kollektiv gut angenommen.

2. Zeitraum

Die Projektförderung hat eine Laufzeit von 4 Jahren, in denen diese klinische Studie sowie alle weiteren projektbezogenen Aktivitäten durchgeführt werden.

3. TeilnehmerInnen

Die Gesamtzahl der TeilnehmerInnen wird basierend auf der Fallzahlberechnung auf 39 ProbandInnen festgelegt

4. Rekrutierung

Die StudienprobandInnen werden unter den TeilnehmerInnen der Betrieblichen Gesundheitsförderung der Salzburger Landeskliniken rekrutiert.

4.1. Einschlusskriterien

- Mindestalter 18 Jahre
- Männer und Frauen
- Weniger als 150 min körperliche Aktivität pro Woche
- Schriftliche Einverständniserklärung für die Teilnahme an der Studie

4.2. Ausschlusskriterien

- Akute oder chronische Herz-Kreislauferkrankungen außer arterielle Hypertonie (systolischer Blutdruck ≥140 mmHg und diastolischer Blutdruck ≥90 mmHg in unbehandelten und medikamentös behandelten TeilnehmerInnen)
- Akute oder chronische Lungenerkrankungen

- Alkohol- (>30g/Tag) oder Drogenabusus
- Adipositas ab Grad 2 (Body Mass Index >35 kg/m²)
- Orthopädische Erkrankungen mit Einschränkung der körperlichen Leistungsfähigkeit
- Schwangere und stillende Frauen

5. Ablauf

Nach erfolgreicher sportmedizinischer Eingangsuntersuchung trainieren die StudienteilnehmerInnen 1x wöchentlich supervidiert am Universitätsinstitut für präventive und rehabilitative Sportmedizin im Rahmen von Ausdauer- und Krafttrainingskursen der Betrieblichen Gesundheitsförderung der Salzburger Landeskliniken. Während diesem 4-monatigen Trainingsblock erhalten die StudienteilnehmerInnen zusätzlich Unterstützung mittels einer digitalen Aktivitätsplanungs- und Aktivitätsdokumentations-App, damit sie Ihre wöchentliche körperliche Aktivität auf >150 min pro Woche erhöhen. Die Ergebnisse werden 1x pro Monat mit dem Trainingstherapeuten besprochen. Nach Ende des Trainingsblocks erfolgt eine sportmedizinische Ausgangsuntersuchung.

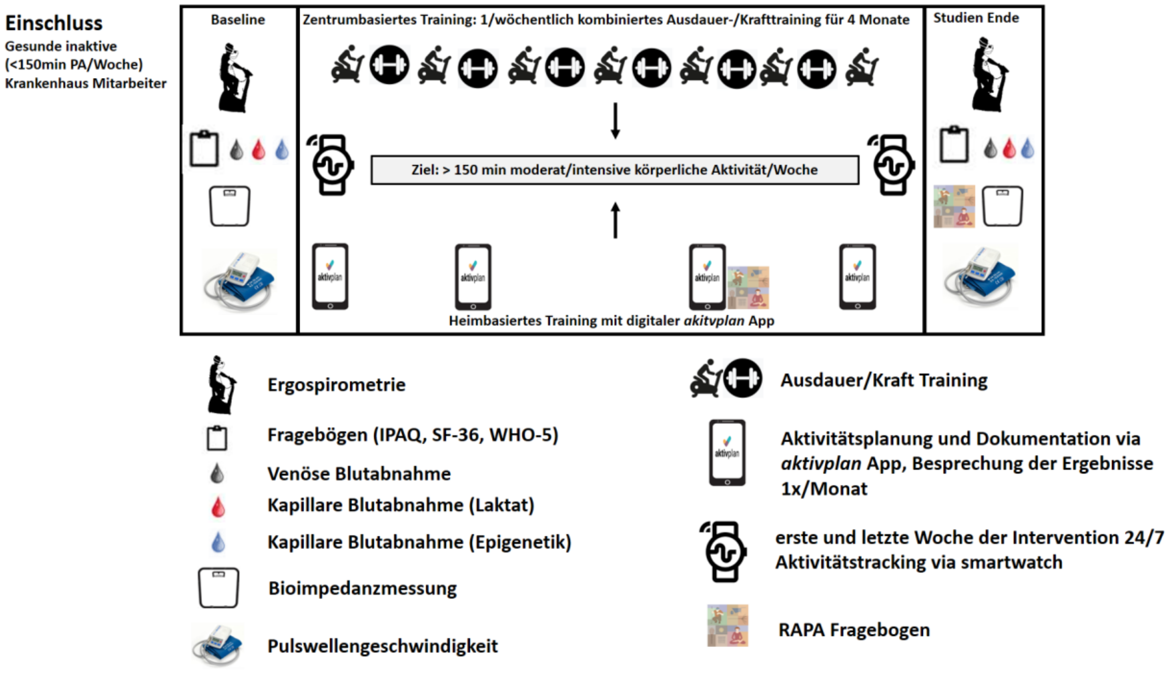

Abbildung 1: Studienablauf

6. Untersuchungen

Die sportmedizinischen Eingangsuntersuchungen bzw. Ausgangsuntersuchungen werden am Universitätsinstitut für präventive und rehabilitative Sportmedizin Salzburg durchgeführt.

Untersuchungen beinhalten:

- Anamneseerhebung
- Ärztliche Untersuchung
- Ermittlung PROCAM-Score [12] , Framingham-Score[13] , ESC-Score [14]
- Anthropometrische Messungen (Größe, Gewicht, Body-Mass-Index, Bauch-/Hüftumfang)
- Fragebögen (Körperliche Aktivität (IPAQ), Gesundheitszustand/Lebensqualität (SF 36), Wohlbefinden (WHO5))
- Lungenfunktionstest (EasyOne®Air, NDD Medizintechnik AG, Zürich, Schweiz)
- Ruhe-EKG (ECGPro, AMEDTEC Medizintechnik Aue GmbH, Aue, Deutschland)
- Messung der Gefäßsteifigkeit (Mobil-o-Graph®, IEM, Stolberg, Deutschland)
- Bioimpedanzmessung (BIA 101 Anniversary Sport Edition, Akern GmbH, Mainz, Deutschland)
- Spiroergometrie inkl. kapillärer Blutabnahme für Laktatbestimmung
- Venöse Blutabnahme:
    - Kleines Blutbild
    - Elektrolyte (Natrium, Kalium, Kalzium)
    - Kreatinin
    - Leberwerte (GPT, Gamma-GT)
    - Cholesterin
    - Triglyceride
    - High-density-lipoprotein (HDL)
    - Low-density-lipoprotein (LDL)
    - HbA1c
    - Glukose (nüchtern)
    - Stickoxidmessung (NO)
- Kapilläre Blutabnahme für epigenetische Untersuchungen (Micro-Ribonukleinsäuren miRNA)

6.1. Pulswellengeschwindigkeit (PWA)

Die Analyse der Gefäßelastizität bzw. Gefäßsteifigkeit mittels einer Pulswellengeschwindigkeitsmessung (Mobil-o-Graph®, IEM, Stolberg, Deutschland) dient der

Erhebung der aktuellen Endothelfunktion und ist ein Prädiktor für kardiovaskuläre Gesundheit. Körperliches Training hat nachweislich einen positiven Einfluss auf die Pulswellengeschwindigkeit [15-17]. In der hier vorliegenden Studie wird analysiert, wie hoch dieser Effekt bei zuvor inaktiven Personen ist, wenn diese über den Studienzeitraum von 4 Monaten die wöchentliche körperliche Aktivität auf über 150 min pro Woche erhöhen und ob diese Veränderung in Zusammenhang mit den untersuchten miRNAs stehen. Die StudienteilnehmerInnen sollen 10 min ruhig in der Messposition (aufrecht sitzende Position, Manschette am nicht-dominaten Arm, welcher auf Herzhöhe entspannt abgelegt ist) verweilen bevor die Messungen gestartet werden. Mindestens 3h vor der Messung soll auf Rauchen sowie die Zufuhr von Nahrungsmitteln und Koffein-haltigen Getränke verzichtet werden. Es erfolgen drei aufeinanderfolgende Messungen mit mind. 1 Minute Abstand zwischen den Messwiederholungen. Die Messung wird bei der Eingangsuntersuchung sowie nach Abschluss der Trainingsphase im Zuge der Ausgangsuntersuchung durchgeführt.

6.2. Bioimpedanzanalyse (BIA)

Es wird eine Ganzkörpermessung mit dem BIA 101 Anniversary Sport Edition (Akern GmbH, Mainz, Deutschland) zur Bestimmung von bioelektrischen Parametern durchgeführt, die Aufschluss über die Körperzusammensetzung der StudienteilnehmerInnen geben. 2h vor der Messung sollte keine Nahrungsaufnahme sowie Flüssigkeitsaufnahme mehr erfolgen. Die StudienteilnehmerInnen liegen in horizontaler Position auf einer Untersuchungsliege. Die Elektroden werden nach Reinigung der Haut mit Alkohol am Gelenksspalt sowie 5 cm distal zur ersten Elektrode auf der linken Hand sowie am linken Sprunggelenk platziert. Nach Anschluss der Analysekabel erfolgt eine einmalige Messung. Die Messung wird bei der Eingangsuntersuchung sowie nach Abschluss der Trainingsphase im Zuge der Ausgangsuntersuchung durchgeführt.

6.3. Spiroergometrie

Die maximale Ausbelastung in Form einer Fahrradergometrie ist Teil der Routine-Eingangsuntersuchung für die Teilnahme an Trainingskursen der Betrieblichen Gesundheitsförderung. Im Rahmen der Studie wird eine Fahrrad-Spiroergometrie durchgeführt. Dabei werden während der gesamten Ergometrie computerunterstützt mittels breath-by-breath Methode die Atemvolumina und-gase gemessen und aufgezeichnet (Cortex MetaLyzer 3B, Leipzig, Germany). Zusätzlich werden zu definierten Zeitpunkten der Blutdruck unter Belastung sowie die Laktatkonzentration (kapillare Blutabnahme am hyperämisierten

Ohrläppchen zu Beginn, am Ende jeder Belastungsstufe, bei maximaler Belastung sowie 3 Minuten nach Belastung) bestimmt. Die Startlast (20-50 Watt) bzw. Stufensteigerung (10-50 Watt) wird so gewählt, dass die TeilnehmerInnen eine Ausbelastung nach 10-15min erreichen. Während der gesamten Untersuchung sind die ProbandInnen mittels 12-Kanal EKG (Amedtec ECGPro, Aue, Germany) überwacht. Das gleiche Protokoll wird nach Abschluss der Trainingsperiode von 4 Monaten erneut getestet.

6.4. Epigenetische Untersuchung

Die notwendigen Blutproben für die epigenetische Untersuchung der miRNAs werden kapillar am hyperämisierten Ohrläppchen abgenommen. Bei den Spiroergometrien bei Ein- und Ausgangsuntersuchung werden Proben in Ruhe sowie unmittelbar nach Abschluss der Ergometrie abgenommen.

Dies dient dazu zugrundeliegende physiologische Anpassungen im Blut zu untersuchen, die während kurzen intensiven Belastungen (Ergometrie) sowie längerer Trainingsbelastung (4 Monate Ausdauer-/Krafttraining) auftreten. Dabei sollen die Veränderungen in der Genexpression von miRNAs durch kurze bzw. längere Belastung analysiert werden. Diese Beobachtungen können dann später dafür verwendet werden, individuelle Langzeiteffekte von körperlichen Training zu evaluieren.

Die epigenetischen Untersuchungen (Expressionsanalysen von miRNAs) dienen rein wissenschaftlichen Zwecken und eine Aufklärung über individuelle Ergebnisse wird nicht erfolgen.

Die RNA wird aus den Plasmaproben mittels einem säulenbasierten Aufreinigungs-Kit extrahiert (NucleoSpin miRNA Plasma Kit; Macherey-Nagel, Düren, Deutschland). Die Expression der miRNAs wird in allen Proben mittels Locked Nucleic Acid (LNA™)-basierendem miRNA qRT-PCR (Qiagen, Hilden, Deutschland) analysiert.

Basierend auf unserem Prädiktor-Modell werden die Expressionshöhen der miRNA miR-150-5p, miR-101-3p, miR-141-3p und miR-200b-3p ermittelt [8]. Zusätzlich dazu die miR-29a-3p und miR-30a-5p aufgrund deren Zusammenhangs mit Plötzlichem Herztod [18], sowie miR-126-3p als Marker für Endothelschäden [19-22], miR-21-5p aufgrund der Rolle in der Anpassung an Hypoxie und Inflammation sowie in der Muskel Kontraktilität [7, 23-25] und miR-146a-5p aufgrund der Rolle in der Anpassung an Hypoxie und Inflammation [7, 19, 23, 26, 27]. Außerdem wird eine Auswahl an miRNAs für die Normalisierung sowie die Qualitätskontrolle analysiert, sodass in Summe je 15 miRNAs pro Probe getestet werden.

6.5. *Ausdauer- und Krafttraining*

Das Ausdauer- und Krafttraining erfolgt im Zuge der Trainingskurse der Betrieblichen Gesundheitsförderung der Salzburger Landeskliniken. Jede Trainingseinheit besteht aus einem Fahrradergometertraining gefolgt von einem an Kraftgeräten durchgeführten Krafttraining. Alle Ergometer-Trainingseinheiten werden auf Ergoselect 200 (Ergoline GmbH, Bitz, Germany) Ergometern durchgeführt und mittels Ergoline Reha Systems (Ergoline GmbH, Bitz, Germany) EKG überwacht. Jede Trainingseinheit beginnt und endet mit 5 min Aufwärmen und endet mit 5 min Abwärmen bei 60–65% peak Herzfrequenz (HR_{peak}), welche bei der Eingangsuntersuchung ermittelt wurde. Die eigentliche Trainingsphase umfasst 25 min und besteht aus 4x4 min Intervallen bei 85-95% HR_{peak} mit 3x3 min aktiver Erholung bei 60-70% HR_{peak}. Die Trainingslast wird graduell an die individuelle Zielherzfrequenz angepasst.

Das Krafttraining besteht aus 3 Serien zu 8-12 Wiederholungen bei 80% des 10-Wiederholungs-Maximums (10-RM) an 10 Krafttrainingsgeräten (Dr. Wolff Sports and Prevention GmbH, Arnsberg, Deutschland) für die oberen und unteren Extremitäten sowie die Rumpfmuskulatur.

6.6. *Aktivitätsplanungs- und Dokumentations-Tool „aktivplan App“*

Mit Hilfe der im Rahmen des Ludwig Boltzmann Instituts für Digitale Gesundheit und Prävention entwickelten *aktivplan* App werden Aktivitäten geplant und dokumentiert.

6.6.1. Anwendung der „*aktivplan*“ App

In der Eingangsphase der Studie werden personenbezogene Daten sowie mittels Ergospirometrie erhobene Daten der StudienteilnehmerInnen in der *aktivplan* Applikation[1] eingegeben und gespeichert. Es handelt sich dabei um folgende Parameter: Vor- und Nachname, eMail, Geburtstag, Körpergröße, Körpergewicht, Aktivitätsklasse, maximale Herzfrequenz, maximaler Blutdruck, maximale Leistung, maximale Sauerstoffaufnahme, Begleiterkrankungen, Medikamente, Foto.

Basierend auf diesen Daten wird ein individuell angepasster Trainingsplan für die StudienteilnehmerInnen erstellt. Dieser Trainingsplan umfasst das wöchentliche zentrumsbasierte Training (Ausdauer-/Krafttraining) und das heimbasierte Training.

[1] Die *aktivplan* Applikation ist für Trainingstherapeuten als Web-Applikation im Browser abrufbar; für Studienteilnehmer ist der *aktivplan* sowohl als Web-Applikation im Browser als auch als mobile Applikation am Handy abrufbar.

Während des 4-monatigen Trainingsblocks können die StudienteilnehmerInnen den Trainingsplan mit Hilfe der *aktivplan* App jederzeit auf dem persönlichen Handy abrufen. So erhalten die TeilnehmerInnen einen Überblick über anstehende Trainingseinheiten sowie bereits absolvierte Aktivitäten. Außerdem wird der Status der körperlichen Aktivität („aktive Minuten") in der aktuellen Kalenderwoche angezeigt. Ziel ist es, diese auf mehr als 150 Minuten pro Woche zu erhöhen. Die Erreichung dieses Ziels wird in der *aktivplan* App entsprechend visualisiert. Sobald eine geplante Aktivität durchgeführt wurde, können die TeilnehmerInnen diese als ausgeführt markieren. Aktivitäten, die zusätzlich zu den mit dem Trainingstherapeuten geplanten Aktivitäten durchgeführt werden, können von den TeilnehmerInnen manuell in die App eingetragen werden. Sämtliche geplanten und ausgeführten Aktivitäten werden über das Backend der *aktivplan* Applikation serverseitig gespeichert. Während der Studie können die Trainingstherapeuten die geplanten und durchgeführten Aktivitäten der jeweiligen StudienteilnehmerInnen jederzeit abrufen. Außerdem werden die in der App dokumentierten und geplanten Aktivitäten 1x pro Monat mit dem Trainingstherapeuten besprochen.

Während des 4-monatigen Trainingsblocks werden Aktivitätsdaten erfasst. Diese Daten beinhalten Art, Häufigkeit, Dauer, Belastungsintensität und subjektiv empfundene Anstrengung der jeweiligen Aktivität. Zur Objektivierung der Intensität bzw. Volumen der körperlichen Aktivitäten während der 4-monatigen Studienphase bekommen die StudienteilnehmerInnen Wearables zur Verfügung gestellt (z.B. Polar OH1 Sensor, Garmin Vivoactive 4/ Vivosmart 4). Jeweils in der ersten sowie in der letzten Trainingswoche wird mittels dieser Wearables die körperliche Aktivität im Alltag mit einer 24/7 Messung aufgezeichnet.

6.6.2. Evaluierung der „*aktivplan*" App

Zur Untersuchung der Annahme, dass eine App-gestützte Planung und Dokumentation einer Trainingsintervention gut angenommen wird, wird der Fokus auf Akzeptanz, Usability und User Experience (Nutzungserleben) der StudienteilnehmerInnen gelegt.

So sollen Motive und Anreize für die Verwendung digitaler Technologien identifiziert, und positive und negative Aspekte im Hinblick auf Aktivitätssteigerung erforscht werden. Besonderes Augenmerk wird dabei auf die Faktoren Kontrolle, Involvement und Engagement gelegt. Um ein umfassendes Bild über die oben genannten Faktoren im Verlauf der Nutzung zu bekommen, findet die Datenerhebung zu mehreren Zeitpunkten statt. Die erste Datenerhebung erfolgt mit der Einweisung in die App nach Abschluss der Eingangsuntersuchung und dient dazu, initiale Erwartungen und Motive vor der tatsächlichen Nutzung zu sammeln. Während der 4-monatigen Trainingsphase werden die oben genannten

Aspekte und Faktoren im Rahmen der monatlichen Besprechungen mit dem Trainingstherapeuten in Form eines semi-strukturierten Interviews erhoben. Die Analyse der Daten soll zeigen, ob die die App-gestützte Planung und Dokumentation einer Trainingsintervention gut angenommen wird bzw. aufzeigen, wo es Verbesserungsmöglichkeiten und Schwierigkeiten gibt, um letztendlich ein nachhaltig einsetzbares Tool im Bereich der Aktivitätsplanung und -dokumentation gestalten zu können.

7. Fallzahlberechnung

Für die Fallzahlberechnung wurden die pre-Ergometrie Mittelwerte, die pre- und post Standardabweichungen und die pre-post Korrelationen der relativen Expression von 9 vorausgewählten miRNAs einer früheren Studie in gesunden untrainierten Teilnehmern herangezogen (siehe Tabelle 1). Für die primäre Analyse wird ein separater gepaarter zweiseitiger t-test für jede der 9 miRNAs durchgeführt werden, und eine statistische Signifikanz wird anhand der Bonferroni-angepassten α-Level = 0,05 / 9 ermittelt. Wir erachten das 4-monatige Training als effektiv, wenn in zumindest 6 der 9 miRNAs einen statistisch signifikante 1,2-fache post-training Veränderung festgestellt werden kann (das heißt, dass angenommen wird, dass der post-trainings Mittelwert gleich log (1,2 * 2^pre) / log (2) ist, unter Verwendung der Standard Formel für die Evaluierung von Expressionsveränderungen bei miRNAs). Wir haben eine minimale Fallzahl berechnet, die notwendig ist um ein 90-prozentige Power in zumindest 6 von den 9 Bonferroni-angepassten miRNA Vergleichen zu erhalten. Die notwendige Fallzahl dafür liegt bei n = 26. Unter der Annahme einer mögliche Drop-out Rate von 1/3 wurde die Fallzahl der hier vorliegenden Studie auf n = 39 TeilnehmerInnen festgelegt.

Tabelle 1: Herangezogene Daten für die Fallzahlberechnung

miRNA	Mittelwert der Relativen Expression	SD	Korrelationskoeffizient
miR-101-3p-Pre	3,89	0,56	0,499
miR-101-3p-Post	3,81	0,50	
miR-126-3p-Pre	4,80	0,44	0,445
miR-126-3p-Post	5,05	0,46	
miR-141-3p-Pre	2,01	0,01	0,349
miR-141-3p-Post	2,01	0,01	
miR-146a-5p-Pre	2,13	0,06	0,611
miR-146a-5p-Post	2,15	0,06	
miR-150-5p-Pre	2,65	0,22	0,411
miR-150-5p-Post	2,99	0,32	

miR-200b-3p-Pre	2,01	0,01	0,305
miR-200b-3p-Post	2,01	0,01	
miR-29a-3p-Pre	2,13	0,04	0,352
miR-29a-3p-Post	2,14	0,05	
miR-30a-5p-Pre	2,04	0,01	0,318
miR-30a-5p-Post	2,04	0,01	
miR-21-5p-Pre	4,72	0,50	0,367
miR-21-5p-Post	4,68	0,50	

8. Ethische Aspekte

Das Studienprotokoll, die Einverständniserklärung sowie alle weiteren Studien-zugehörigen Dokumente werden dem Ethikkomitee des Landes Salzburg vorgelegt. Die Studie wird nach den Richtlinien der Declaration of Helsinki sowie der Good Clinical Practice (GCP) durchgeführt. Des Weiteren wird die Studie bei ClinicalTrials.gov registriert.

Durch die gewissenhafte Anwendung relevanter Sicherheitsrichtlinien sind keine Risiken für die TeilnehmerInnen zu erwarten. Die Aufklärung der TeilnehmerInnen erfolgt laut Aufklärungsbogen durch einen Arzt.

Die erfassten Daten werden indirekt personenbezogen (pseudonymisiert) verarbeitet. Es werden keinerlei direkt personenbezogene Daten verarbeitet.

9. Versicherung

Die Studien TeilnehmerInnen sind während der Teilnahme an der Studie bei der HDI Versicherung AG unter folgender Polizzennummer 5192355 versichert.

10. Studienabbruch

Die Studie kann bei unvorhergesehenen, schwerwiegenden Ereignissen jederzeit und ohne einen Nachteil für die teilnehmenden Personen abgebrochen werden. Ein reguläres Abbruch-Kriterium ist nicht vorgesehen.

Die einzelnen Teilnehmer haben ungeachtet ihrer Beweggründe jederzeit das Recht, ihre Teilnahme an der Studie vorzeitig zu beenden, und sie werden auf diese Möglichkeit im Zuge ihrer Aufklärung hingewiesen. Es wird mit einer Drop-out-Quote von maximal 1/3 gerechnet.

11. Datenaufbewahrung

Die Daten bzw. Blutproben werden zugriffsbeschränkt gespeichert bzw. gelagert. Diese Speicherung/Lagerung der pseudonymisierten Daten dauert bis zur vollständigen Auswertung, bzw. maximal 10 Jahre.

Der Schutz vor dem Zugriff Unbefugter ist durch Zutrittsbeschränkung zu den Lagerorten und durch Verschluss der Lagerschränke sichergestellt. Sollten die Teilnehmer mit der Speicherung bzw. Aufbewahrung nicht einverstanden sein, werden die Daten gelöscht bzw. das Probenmaterial vernichtet. Die Verantwortung für die Vernichtung des Probenmaterials trägt Univ.-Prof. Dr.Dr. Josef Niebauer, MBA.

Die Verarbeitung der Daten erfolgt in pseudonymisierter Form.

12. Auswertemethoden

Daten Analyse erfolgt mittels IBM SPSS Statistics Software (Version 24.0, SPSS, Inc., Chicago, IL) am Universitätsinstitut für präventive und rehabilitative Sportmedizin. Deskriptive Statistik wird durchgeführt unter Verwendung von Mittelwert und Standardabweichung für normalverteilte Daten beziehungsweise Median und Range für nicht normalverteilte Daten. Zweiseitige, gepaarte T-tests mit Korrektur für Multiples Testen werden verwendet um die akut Effekte auf Bewegung zu analysieren. Bei nicht normalverteilten Daten wird entsprechend ein Wilcoxon signed-rank test angewendet. ANOVA mit Messwiederholung wird verwendet um signifikante Unterschiede zwischen den Bewegungsinterventionen, Ruhe und Ausbelastungswerten zu analysieren. Je nach Verteilung der Daten erfolgen die Korrelationsberechnungen nach Pearson bzw. Spearman-Rho. Als Signifikanzniveau wird $p<0.05$ angenommen.

13. Sicherheit

Ergometrie: Im Rahmen der Ergometrie werden die TeilnehmerInnen bis an ihre Belastungsgrenze belastet. Um eventuelle gesundheitliche Risiken schon vor der Ergometrie zu erkennen werden die TeilnehmerInnen vorher ausführlich in einem Anamnesegespräch über mögliche Kontraindikationen für eine Belastung befragt. Zudem werden die ProbandInnen gründlich körperlich untersucht, wird ein Elektrokardiogramm in Ruhe geschrieben und der Blutdruck gemessen. Sollten weitere Untersuchungen notwendig werden (z.B. ein Herzultraschall, Labor, etc.), so werden diese durchgeführt. Somit besteht eine

maximale Sicherheit für die TeilnehmerInnen vor Beginn der Ergometrie. Selten (<0.01%) kann es zu Zwischenfällen wie gefährliche Herzrhythmusstörungen, Synkopen oder noch seltener zum Tod kommen [28]. Geschultes, professionelles Personal ist während der gesamten Untersuchungen anwesend und kann bei Zwischenfällen sofort reagieren.
Während körperlichem Training können ungünstige Ereignisse wie außergewöhnliche Blutdruckschwankungen, Atemnot, Schwindel oder Herzrhythmusstörungen auftreten. Zusätzlich sind auch in Extremfällen Vorfälle von Angina Pectoris möglich. Daher stehen die PatientInnen während des zentrumbasierten Trainings unter permanenter ärztlicher Aufsicht und trainieren unter EKG-Überwachung. Zu den unerwünschten Risiken von Training zählen zudem Verletzungen am Bewegungsapparat und der Muskelkater. Das Risiko für beide Ereignisse kann angesichts der qualifizierten Betreuung und individuellen Trainingsanpassung auf ein sehr geringes Niveau reduziert werden. Um eine Überforderung der ProbandInnen zu verhindern, wird die Trainingsintensität ständig überwacht und ggf. reduziert.

14. Datenschutz

Alle Daten und Informationen, die während der Studie gesammelt werden, sowie alle TeilnehmerInnen-bezogenen Daten erfüllen die Standards zum Datenschutz in Übereinstimmung mit der Datenschutz-Grundverordnung (DSGVO) von 2016 in Bezug auf den Umgang mit Personendaten. Die Analyse sowie die Zusammenfassung der Studie wird mit anonymisierten Daten sowie Daten ohne Referenz zu spezifischen StudienteilnehmerInnen durchgeführt. Zugriff auf Akten von StudienteilnehmerInnen wird limitiert auf Forschungsmitarbeiter des Universitätsinstituts für präventive und rehabilitative Sportmedizin Salzburg.
Die Nutzung der *akivplan* App und die damit verbundene Datenverarbeitung erfolgt auf dem Server der Alphaport OG. Personenbezogene (beispielsweise Name, Alter) bzw. sensible Daten (Gesundheitsdaten wie z.B. maximale Herzfrequenz, maximaler Blutdruck) werden auf dem von Chino.io bereitgestellten DSGVO-zertifizierten Server gespeichert. Zum Zuordnen der Benutzer wird seitens Alphaport auf die pseudonymisierte Benutzer-ID von Chino.io verwiesen. Über entsprechende Schnittstellen greift das Backend mittels der pseudonymisierten Benutzer-ID auf die bei Chino.io gespeicherten sensiblen Daten zu und gibt diese am Frontend aus.

In der Eingangsphase der Studie werden die StudienteilnehmerInnen über die App und die damit verbundenen datenschutzrechtlichen Aspekte aufgeklärt. Insbesondere werden folgende Punkte erläutert und per digitalem Timestamp dokumentiert:

o **Ziel**: Die *aktivplan* App soll die GesundheitsexpertInnen und die StudienteilnehmerInnen dabei unterstützen, Trainingsaktivitäten individuell zu planen und optimal an den Gesundheitszustand der PatientInnen anzupassen. Die App bietet den StudienteilnehmerInnen die Möglichkeit, die mit den GesundheitsexpertInnen geplanten Aktivitäten am Handy anzusehen und zu verfolgen, sowie zusätzliche Aktivitäten einzutragen.

o **Ablauf**: Wenn die StudienteilnehmerInnen sich explizit zur Nutzung der *aktivplan* App bereit erklären, werden die im Leistungstest erhobenen Daten von den GesundheitsexpertInnen eingegeben. So wird eine Basis für einen individuell angepassten Trainingsplan geschaffen (zum Beispiel Training im optimalen Herzfrequenzbereich, mit der optimalen Belastung.) Nach der Eingabe erhalten die StudienteilnehmerInnen ein E-mail mit einem Link. Wird der Link nicht angeklickt so werden nach 10 Tagen die eingegebenen Daten gelöscht. Wird der Link innerhalb von 10 Tagen angeklickt, so erfolgt eine Aufklärung der StudienteilnehmerInnen hinsichtlich der geltenden Datenschutzerklärung (insbesondere der Verarbeitung sensibler, gesundheitsbezogener Daten) und der geltenden AGB's. Nach Zustimmung der StudienteilnehmerInnen zu oben genannten Punkten kann die aktivplan App auf dem Handy installiert werden.

o **Datenschutz und Datenverwertung**: Alle Information werden vertraulich behandelt.

Die personenbezogenen Daten werden pseudonymisiert und auf sicheren Servern gespeichert (DSGVO konform). Die aufgenommenen Daten dienen dazu, dass ein individuell angepasstes und optimal an den Gesundheitszustand abgestimmtes Training erstellt wird. (Zum Beispiel Training im optimalen Herzfrequenzbereich)

o **Freiwillige Teilnahme und Rücktrittsfreiheit**: Ihre Wahl die *aktivplan* App zu verwenden ist voll und ganz freiwillig. Wenn die StudienteilnehmerInnen innerhalb von 10 Tagen nicht den Link im E-mail anklicken, dass sie zur Registrierung der App bekommen haben, werden die aufgenommenen Daten automatisch gelöscht. Nach Registrierung kann die Verwendung der *aktivplan* App jederzeit beendet werden, was die Löschung sämtlicher Daten des Studienteilnehmers / der Studienteilnehmerin beinhaltet.

o **Fragen**: Bei Fragen kann jederzeit Kontakt aufgenommen werden.

15. Berichterstattung

Endbericht

Ein Endbericht über diese Studie wird geschrieben, unabhängig von den Ergebnissen. Dieser Bericht wird den Zuständigen der Prosperamus! Förderstelle sowie dem zuständigen Ethikkomitee vorgelegt.

Publikation

Ziel ist es die Ergebnisse der Studie in einem wissenschaftlichen Fachjournal mit peer-review Verfahren zu publizieren. Das Ethikkomitee wird über die finalen Ergebnisse der Studie informiert.

16. Referenzen

1. *Global, regional, and national age-sex specific all-cause and cause-specific mortality for 240 causes of death, 1990-2013: a systematic analysis for the Global Burden of Disease Study 2013.* Lancet, 2015. **385**(9963): p. 117-71.
2. Symonds, M.E., S. Sebert, and H. Budge, *The obesity epidemic: from the environment to epigenetics - not simply a response to dietary manipulation in a thermoneutral environment.* Front Genet, 2011. **2**: p. 24.
3. Schulte, C. and T. Zeller, *microRNA-based diagnostics and therapy in cardiovascular disease-Summing up the facts.* Cardiovasc Diagn Ther, 2015. **5**(1): p. 17-36.
4. Sun, X., N. Belkin, and M.W. Feinberg, *Endothelial microRNAs and atherosclerosis.* Curr Atheroscler Rep, 2013. **15**(12): p. 372.
5. Santulli, G., *microRNAs and Endothelial (Dys) Function.* Journal of cellular physiology, 2015.
6. Nabel, E.G. and E. Braunwald, *A tale of coronary artery disease and myocardial infarction.* N Engl J Med, 2012. **366**(1): p. 54-63.
7. Baggish, A.L., et al., *Dynamic regulation of circulating microRNA during acute exhaustive exercise and sustained aerobic exercise training.* J Physiol, 2011. **589**(Pt 16): p. 3983-94.
8. Mayr, B., et al., *Exercise responsive micro ribonucleic acids identify patients with coronary artery disease.* Eur J Prev Cardiol, 2019. **26**(4): p. 348-355.
9. Goodman, J., S. Thomas, and J.F. Burr, *Physical activity series: cardiovascular risks of physical activity in apparently healthy individuals: risk evaluation for exercise clearance and prescription.* Can Fam Physician, 2013. **59**(1): p. 46-9, e6-e10.
10. Whitfield, G.P., et al., *Trends in Meeting Physical Activity Guidelines Among Urban and Rural Dwelling Adults - United States, 2008-2017.* MMWR Morb Mortal Wkly Rep, 2019. **68**(23): p. 513-518.
11. Bull, F.C., et al., *World Health Organization 2020 guidelines on physical activity and sedentary behaviour.* Br J Sports Med, 2020. **54**(24): p. 1451-1462.
12. Assmann, G., P. Cullen, and H. Schulte, *Simple scoring scheme for calculating the risk of acute coronary events based on the 10-year follow-up of the prospective cardiovascular Munster (PROCAM) study.* Circulation, 2002. **105**(3): p. 310-5.

13. Expert Panel on Detection, E. and A. Treatment of High Blood Cholesterol in, *Executive Summary of The Third Report of The National Cholesterol Education Program (NCEP) Expert Panel on Detection, Evaluation, And Treatment of High Blood Cholesterol In Adults (Adult Treatment Panel III).* JAMA, 2001. **285**(19): p. 2486-97.
14. Perk, J., et al., *European Guidelines on cardiovascular disease prevention in clinical practice (version 2012). The Fifth Joint Task Force of the European Society of Cardiology and Other Societies on Cardiovascular Disease Prevention in Clinical Practice (constituted by representatives of nine societies and by invited experts).* Eur Heart J, 2012. **33**(13): p. 1635-701.
15. Weber, T., et al., *Wave reflections, assessed with a novel method for pulse wave separation, are associated with end-organ damage and clinical outcomes.* Hypertension, 2012. **60**(2): p. 534-41.
16. Boutouyrie, P., et al., *Aortic stiffness is an independent predictor of primary coronary events in hypertensive patients: a longitudinal study.* Hypertension, 2002. **39**(1): p. 10-5.
17. Cruickshank, K., et al., *Aortic pulse-wave velocity and its relationship to mortality in diabetes and glucose intolerance: an integrated index of vascular function?* Circulation, 2002. **106**(16): p. 2085-90.
18. Silverman, M.G., et al., *Circulating miRNAs and Risk of Sudden Death in Patients With Coronary Heart Disease.* JACC Clin Electrophysiol, 2020. **6**(1): p. 70-79.
19. Baggish, A.L., et al., *Rapid up-Regulation and Clearance of Distinct Circulating Micrornas after Prolonged Aerobic Exercise.* Cardiology, 2014. **128**: p. 442-442.
20. Liu, Y., et al., *The role of circulating microRNA-126 (miR-126): a novel biomarker for screening prediabetes and newly diagnosed type 2 diabetes mellitus.* Int J Mol Sci, 2014. **15**(6): p. 10567-77.
21. Zhou, J., et al., *Associations between physical activity-related miRNAs and metabolic syndrome.* Horm Metab Res, 2014. **46**(3): p. 201-5.
22. Uhlemann, M., et al., *Circulating microRNA-126 increases after different forms of endurance exercise in healthy adults.* Eur J Prev Cardiol, 2014. **21**(4): p. 484-91.
23. Wardle, S.L., et al., *Plasma microRNA levels differ between endurance and strength athletes.* PLoS One, 2015. **10**(4): p. e0122107.
24. Cheng, Y., et al., *Ischaemic preconditioning-regulated miR-21 protects heart against ischaemia/reperfusion injury via anti-apoptosis through its target PDCD4.* Cardiovasc Res, 2010. **87**(3): p. 431-9.
25. Thum, T., et al., *MicroRNA-21 contributes to myocardial disease by stimulating MAP kinase signalling in fibroblasts.* Nature, 2008. **456**(7224): p. 980-4.
26. Sawada, S., et al., *Profiling of circulating microRNAs after a bout of acute resistance exercise in humans.* PLoS One, 2013. **8**(7): p. e70823.
27. Nielsen, S., et al., *The miRNA plasma signature in response to acute aerobic exercise and endurance training.* PLoS One, 2014. **9**(2): p. e87308.
28. Wonisch, M., et al., *Praxisleitlinien Ergometrie.* Atemwegs- und Lungenkrankheiten, 2014. **40**.
