# Supplementary material for: Effects of structured exercise training on miRNA expression in previously sedentary individuals
Source: PLoS One. 2024 Dec 18;19(12):e0314281. doi: 10.1371/journal.pone.0314281 (PMC11654927; doi:10.1371/journal.pone.0314281)
Supplement: S2 File — (PDF) [file pone.0314281.s010.pdf]

STUDYPROTOCOL

TITEL of the study

Impact of a 4 months training intervention on exercise-induced changes of heart health-related epigenetic markers in healthy, sedentary individuals

AKRONYM

miRNA-4-Heart

PROTOCOL VERSION WITH DATE

Version 1.9 - 03.02.2021

STUDYREFERENCENUMBERS

**Ethics committee number:**	1207/2020
**Number of project funding:**	P-20/01/008-NIB
**Clinical Trials number:**	NCT04791306

CONTACT INFORMATION

**Study lead**	Dr.rer.nat Barbara Mayr, BSc, MSc<br><br>University Institute of Sports Medicine, Prevention and Rehabilitation, Paracelsus Medical University Salzburg<br>Lindhofstraße 20<br>5020 Salzburg<br>Österreich
**Sponsor**	**University Institute of Sports Medicine, Prevention and Rehabilitation, Paracelsus Medical University Salzburg**<br><br>Representative from sponsor:<br>Univ.- Prof. Dr. Dr. Josef Niebauer, MBA<br>University Institute of Sports Medicine, Prevention and Rehabilitation, Paracelsus Medical University Salzburg<br>Lindhofstraße 20<br>5020 Salzburg<br><br>and<br><br>Ludwig Boltzmann Institute for Digital Health and Prevention<br>5020 Salzburg<br>Österreich<br><br>**Paracelsus Medical University**<br><br>Contact for research funding program Prosperamus!:<br>Mag. Dorothea Kölblinger, MAS,<br>Strubergasse 21<br>5020 Salzburg<br>Österreich

**Contributor to this study protocol**	Priv.-Doz. Dr. Dr. med. Mahdi Sareban<br><br>University Institute of Sports Medicine, Prevention and Rehabilitation, Paracelsus Medical University Salzburg<br>Lindhofstraße 20<br>5020 Salzburg<br>Österreich<br><br>and<br><br>Ludwig Boltzmann Institute for Digital Health and Prevention<br>Lindhofstraße 22<br>5020 Salzburg<br>Österreich<br><br>Michael Neudorfer, M.Sc., M.Ed.<br><br>University Institute of Sports Medicine, Prevention and Rehabilitation, Paracelsus Medical University Salzburg<br>Lindhofstraße 20<br>5020 Salzburg<br>Österreich<br><br>Mag. Dr. Daniela Wurhofer, Bakk. techn.<br><br>Ludwig Boltzmann Institute for Digital Health and Prevention<br>Lindhofstraße 22<br>5020 Salzburg<br>Österreich<br><br>Dr. Georg Zimmermann<br><br>Team Biostatistics and Big Medical Data, IDA Lab Salzburg<br>Forschungsmanagement & Technologietransfer, Paracelsus Medical University Salzburg<br>Strubergasse 16<br>A-5020 Salzburg

Table of content

CONTACT INFORMATION 2
1. **Background and overview** 4
   1.1. ***Primary Hypotheses*** 5
   1.2. ***Secondary Hypotheses*** 5
2. **Study Period** 5
3. **Participants** 5
4. **Recruitment** 6
   4.1. ***Inclusion criteria*** 6
   4.2. ***Exclusion criteria*** 6
5. **Study plan** 6
6. **Methods** 7
   6.1. ***Pulse wave analysis (PWA)*** 8
   6.2. ***Bio impedance analysis (BIA)*** 8
   6.3. ***Cardio-pulmonary exercise test (CPET)*** 9
   6.4. ***Epigenetic examinations*** 9
   6.5. ***Endurance- and strength training*** 9
   6.6. ***Activity planning- and documentation tool „aktivplan App“*** 10
   6.6.1. Usage of the „*aktivplan*“ App 10
   6.6.2. Evaluation of the „*aktivplan*“ App 10
7. **Sample size calculation** 11
8. **Ethical aspects** 11
9. **Insurance** 12
10. **Study termination** 12
11. **Data storage** 12
12. **Data analysis** 12
13. **Safety** 12
14. **Data security** 13
15. **Reporting** 14
16. **Reference** 14

1. Background and overview

Cardiovascular diseases represent the most common cause of death worldwide [1]. In addition to the genetic, non-modifiable predisposition to such diseases, there are other epigenetic risk markers that are modifiable [2]. Epigenetics refers to changes in gene expression without altering the DNA

sequence. Mechanistically, these changes are caused, among other things, by micro ribonucleic acids (miRNAs). The level of certain miRNAs has been associated with the risk of some cardiovascular diseases such as coronary heart disease [3-6]. These epigenetic changes can be influenced by lifestyle factors, especially physical activity [2, 7]. In a cross-sectional study, our research group was able to demonstrate that the response of potentially cardio protective miRNAs to a single exercise intervention, in the form of ergometry, significantly differs between patients with coronary heart disease and a healthy population [8]. Furthermore, it has been shown in a healthy population that repetitive training stimuli also have an influence on specific miRNAs. This, in turn, suggests epigenetic adaptation mechanisms to the exercise training stimuli [7]. However, these longitudinal studies primarily examined young, healthy, and physically active men. Overall, these data emphasize the role of miRNAs as potential biomarkers for personalized lifestyle modification recommendations for the primary prevention of cardiovascular diseases. The need to optimize the effectiveness of physical activity recommendations is illustrated by the fact that only 20% of the population in industrialized countries achieve the WHO recommendations of 150 minutes per week of moderate or 75 minutes per week of vigorous physical activity [9-11]. One possibility for personalization is the use of epigenetic expression patterns in response to acute exercise interventions, as well as the longitudinal examination of these epigenetic changes following guideline-compliant optimization of exercise behaviour.

For this purpose, this study aims to investigate how the expression pattern of specific miRNAs, which have been associated with the reduction of cardiovascular endpoints in the literature, changes in response to a single acute exercise intervention in a healthy but physically inactive population. Additionally, in this population, this response will be examined after 4 months of regular physical training (>150 minutes/week). To ensure that study participants achieve the desired level of activity and to assess the intensity component, participants will engage in centre-based and supervised endurance and strength training once a week, and will receive support for their independent home-based training through a digital activity planning and documentation app. The corresponding study hypotheses are:

1.1. Primary Hypotheses

A 4-month training intervention in a healthy, physically inactive population leads to an exercise-induced alteration of the expression profile of cardio protective miRNAs.

1.2. Secondary Hypotheses

The exercise-induced alteration of cardio protective miRNAs after a 4-month training intervention correlates with changes in cardiovascular risk factors (weight, blood lipids, vascular stiffness, physical performance).

A digital app-supported training intervention is well received by a healthy, physically inactive population.

2. Study Period

The project funding has a timeframe of 4 years, during which this clinical study as well as all project related activities will be performed.

3. Participants

Based on the sample size calculation the total number of participants were set to 39.

4. Recruitment

The study participants will be recruited amongst the participants of the corporate well-being program of the University hospital of Salzburg.

4.1. Inclusion criteria

- <150min physical activity per week
- >18 years of age
- male and female
- signed inform consents

4.2. Exclusion criteria

- acute or chronic cardiovascular diseases, exempt arterial hypertension (systolic blood pressure >140 mmHg and diastolic blood pressure >90 mmHg in untreated and medical treated subjects)
- acute or chronic lung disease
- alcohol (>30g/day) or drug abuse
- Adipositas grad 2 (BMI >35kg/m^2)
- orthopaedic limitations to exercise capacity
- pregnant or breast feeding women

5. Study plan

After successful initial examination, the study participants train once a week under supervision at the University Institute of Sports Medicine, Prevention and Rehabilitation as part of endurance and strength training courses offered by the Corporate Health Promotion of the University Hospital Salzburg. During this 4-month training period, participants receive additional support through a digital activity planning and activity documentation app to increase their weekly physical activity to >150 minutes per week. The results are discussed with the training therapist once a month. After completion of the training period, a final examination is conducted.

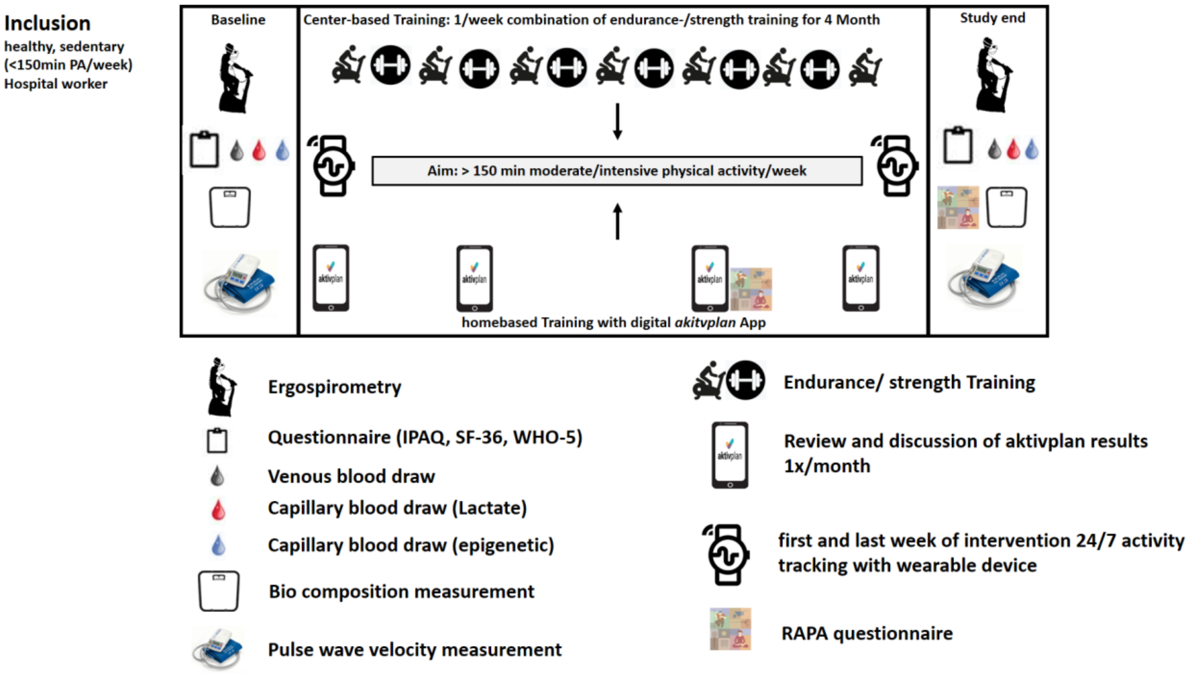

Figure 1: Study plan

6. Methods

The baseline and final examination will be conducted at the University Institute of Sports Medicine, Prevention and Rehabilitation, Paracelsus Medical University Salzburg. The examinations contains:

- Anamneses
- Physical examination by a medical doctor
- Evaluation of PROCAM-Score [12], Framingham-Score [13], ESC-Score [14]
- Anthropometric measurements (height, weight, Body-Mass-Index, Hip-/waist circumference)
- Questionnaires (physical activity (IPAQ), General Health/Quality of health (SF 36), Wellbeing (WHO5))
- Lung function testing (EasyOne®Air, NDD Medizintechnik AG, Zürich, Swiss)
- Resting electrocardiogram (ECG) (ECGPro, AMEDTEC Medizintechnik Aue GmbH, Aue, Germany)
- Measurement of pulse wave analysis (Mobil-o-Graph®, IEM, Stolberg, Germany)
- Bio impedance analysis (BIA 101 Anniversary Sport Edition, Akern GmbH, Mainz, Deutschland)
- Cardio-pulmonary exercise test incl. capillary blood sampling for lactate measurements
- Venous blood sampling:
  - blood count

    - electrolytes (sodium, potassium, calcium)
    - Creatinine
    - Liver parameter (GPT, Gamma-GT)
    - blood cholesterol
    - triglyceride
    - High-density-lipoprotein (HDL)
    - Low-density-lipoprotein (LDL)
    - HbA1c
    - glucose (fasted)
    - Nitrite Oxide (NO)
- Capillary blood sampling for epigenetic measurements (micro ribonucleic acid (miRNA))

6.1. Pulse wave analysis (PWA)

The analysis of vascular elasticity or stiffness using pulse wave velocity measurement (Mobil-o-Graph®, IEM, Stolberg, Germany) serves to assess current endothelial function and is a predictor of cardiovascular health. Physical training has been shown to have a positive effect on pulse wave velocity [15-17]. In the present study, we will analyse the extent of this effect in previously inactive individuals who increase their weekly physical activity to over 150 minutes per week over the 4-month study period, and whether this change is associated with the miRNAs under investigation. Participants will remain quietly in the measurement position (sitting upright, cuff on the non-dominant arm, which is relaxed and placed at heart level) for 10 minutes before the measurements begin. Participants should abstain from smoking, food intake, and consumption of caffeine-containing beverages for at least 3 hours before the measurement. Three consecutive measurements will be taken with at least 1 minute interval between repetitions. The measurement will be performed at the baseline examination and at the end of the training phase as part of the final examination.

6.2. Bio impedance analysis (BIA)

A full-body measurement is performed using the BIA 101 Anniversary Sport Edition (Akern GmbH, Mainz, Germany) to determine bioelectrical parameters that provide insight into the body composition of the study participants. No food or fluid intake should occur 2 hours prior to the measurement. Participants lie in a horizontal position on an examination couch. Electrodes are placed at the joint crease and 5 cm distal to the first electrode on the left hand, as well as on the left ankle after cleaning the skin with alcohol. After connecting the analysis cables, a single measurement is taken. The measurement is conducted at the baseline examination and at the end of the training phase as part of the final examination.

6.3. Cardio-pulmonary exercise test (CPET)

As part of the baseline examination for the study, a cardio pulmonary exercise test (CPET) is performed. Throughout the CPET, gas exchange and ventilation are recorded, using computer-assisted breath-by-breath method (Cortex MetaLyzer 3B, Leipzig, Germany). Additionally, blood pressure and lactate concentration (capillary blood sampling at the hyperemic earlobe at the beginning, end of each workload stage, at maximum workload, and 3 minutes after workload) are measured at defined time points. The starting load (20-50 watts) or step increase (10-50 watts) is chosen so that participants reach maximum exertion after 10-15 minutes. Participants are monitored throughout the examination using a 12-lead ECG (Amedtec ECGPro, Aue, Germany). The same protocol is repeated after completion of the 4-month training period.

6.4. Epigenetic examinations

The necessary blood samples for the epigenetic examination of miRNAs are taken via capillary blood draw from the hyperaemic earlobe. Samples are taken at rest and immediately after completing the CPET at baseline and at the final examination. This is done to examine underlying physiological adaptations in the blood that occur during short intense exercise (ergometry) and longer training loads (4 months of endurance/strength training). The changes in miRNA gene expression due to short and longer duration exercise are analysed. These observations can later be used to evaluate individual long-term effects of physical training. The epigenetic examinations (miRNA expression analyses) serve purely scientific purposes and individual results will not be disclosed. RNA is extracted from plasma samples using a column-based purification kit (NucleoSpin miRNA Plasma Kit; Macherey-Nagel, Düren, Germany). The expression of miRNAs in all samples is analysed using Locked Nucleic Acid (LNA™)-based miRNA qRT-PCR (Qiagen, Hilden, Germany). Based on our predictor model, the expression levels of miRNA miR-150-5p, miR-101-3p, miR-141-3p, and miR-200b-3p are determined [8]. Additionally, miR-29a-3p and miR-30a-5p are included due to their association with sudden cardiac death [18], as well as miR-126-3p as a marker for endothelial damage [19-22], miR-21-5p due to its role in adaptation to hypoxia and inflammation as well as muscle contractility [7, 23-25], and miR-146a-5p due to its role in adaptation to hypoxia and inflammation [7, 19, 23, 26, 27]. Furthermore, a selection of miRNAs for normalization and quality control is analysed, resulting in a total of at least 15 miRNAs per sample.

6.5. Endurance- and strength training

Endurance and strength training are conducted as part of the training courses for corporate health promotion of the University hospital Salzburg. Each training session consists of a bicycle ergometer training followed by strength training performed on strength training machines. All supervised endurance-training sessions are conducted on Ergoselect 200 ergometers (Ergoline GmbH, Bitz, Germany) and ECG monitored using Ergoline Reha Systems (Ergoline GmbH, Bitz, Germany). Each training session begins with a 5-minute warm-up and ends with a 5-minute cool-down at 60–65% peak heart rate (HRpeak) determined at the baseline examination. The actual training phase lasts 25 minutes and consists of 4x4-minute intervals at 85-95% HRpeak with 3x3-minute active recovery intervals at 60-70% HRpeak. The training load is gradually adjusted to the individual target heart rate.

Strength training consists of 3 sets of 8-12 repetitions at 80% of the 10-repetition maximum (10-RM) on 10 strength training machines (Dr. Wolff Sports and Prevention GmbH, Arnsberg, Germany) for the upper and lower extremities as well as the trunk muscles.

6.6. Activity planning- and documentation tool „aktivplan App“

With the help of the *aktivplan* app developed by the Ludwig Boltzmann Institute for Digital Health and Prevention, the physical activities will be planned and documented.

6.6.1. Usage of the „*aktivplan*“ App

During the initial phase of the study, personal data as well as data collected through cardiopulmonary exercise testing (CPET) of the study participants are entered and stored in the *aktivplan* application. The parameters include: first and last name, email, date of birth, height, weight, activity level, maximum heart rate, maximum blood pressure, maximum power output, maximum oxygen uptake, comorbidities, medications, and photo.

Based on this data, an individually tailored training plan is created for the study participants, in a shared decision making process together with a health professional. This training plan includes both the weekly centre-based training (endurance/strength training) and home-based training.

Throughout the 4-month training period, study participants can access the training plan at any time on their personal mobile phones using the *aktivplan* app. This allows participants to see upcoming training sessions as well as activities already completed. Additionally, the status of physical activity ("active minutes") in the current calendar week is displayed, with the goal of increasing this to more than 150 minutes per week. Achievement of this goal is visualized within the *aktivplan* app. Once a planned activity has been completed, participants can mark it as completed. Participants can, manually enter activities performed in addition to those planned, into the app. All planned and completed activities are stored server-side via the backend of the *aktivplan* application. During the study, health professionals can access the planned and completed activities of the respective study participants at any time. Additionally, the activities documented and planned in the app are discussed with the health professionals once a month.

During the 4-month training period, activity data is collected. These data include the type, frequency, duration, intensity, and subjectively perceived exertion of each activity. To objectify the intensity and volume of physical activities during the 4-month study phase, study participants are provided with wearables (e.g., Polar OH1 Sensor, Garmin Vivoactive 4/ Vivosmart 4). In the first and last training weeks, physical activity in daily life is recorded using these wearables with a 24/7 measurement.

6.6.2. Evaluation of the „*aktivplan*“ App

To investigate the assumption that an app-supported planning and documentation of a training intervention is well-received, the focus is placed on the acceptance, usability, and user experience of the study participants. Motives and incentives for the use of digital technologies are to be identified, and positive and negative aspects regarding increased activity are explored. Special attention is paid to the factors of control, involvement, and engagement. In order to obtain a comprehensive picture of the aforementioned factors over the course of use, data collection takes place at multiple time points. The first data collection occurs with the introduction to the app after completion of the baseline examination and serves to gather initial expectations and motives before actual use. During the 4-month training phase, the aforementioned aspects and factors are collected during monthly discussions with the training therapist in the form of semi-structured interviews. The analysis of the data aims to demonstrate whether the app-supported planning and documentation of a training intervention is well-received or to identify areas for improvement and difficulties, ultimately aiming to create a sustainably usable tool in the field of activity planning and documentation.

7. Sample size calculation

For sample size calculation, the pre-ergometry means, pre- and post standard deviations and pre-post correlations of relative expression values corresponding to 9 pre-selected miRNAs were taken from a previous study with healthy, untrained volunteers (see Table 1). For the primary analysis, paired two-sided t-tests will be conducted for each of the 9 miRNAs separately, and statistical significance will be assessed at the Bonferroni-adjusted level alpha = 0.05 / 9. We would consider the 4-months training effective if in at least 6 of the 9 miRNAs, a statistically significant 1.2-fold post-ergometry change could be observed (i.e., the post-ergometry mean was assumed to be equal to log(1.2 x 2^pre) / log(2), using the standard formula for assessing change in miRNAs). We finally calculated the minimum sample size that would be required to obtain 90 percent power in at least 6 of the 9 Bonferroni-adjusted miRNA comparisons. The required sample size was found to be n = 26. Consequently, assuming a drop-out rate of 1/3, the required sample size for the present study would be n = 39 subjects.

Table 1: Used data for sample size calculation

miRNA	Mean relative expression	SD	correlation coefficient
miR-101-3p-Pre	3,89	0,56	0,499
miR-101-3p-Post	3,81	0,50	
miR-126-3p-Pre	4,80	0,44	0,445
miR-126-3p-Post	5,05	0,46	
miR-141-3p-Pre	2,01	0,01	0,349
miR-141-3p-Post	2,01	0,01	
miR-146a-5p-Pre	2,13	0,06	0,611
miR-146a-5p-Post	2,15	0,06	
miR-150-5p-Pre	2,65	0,22	0,411
miR-150-5p-Post	2,99	0,32	
miR-200b-3p-Pre	2,01	0,01	0,305
miR-200b-3p-Post	2,01	0,01	
miR-29a-3p-Pre	2,13	0,04	0,352
miR-29a-3p-Post	2,14	0,05	
miR-30a-5p-Pre	2,04	0,01	0,318
miR-30a-5p-Post	2,04	0,01	
miR-21-5p-Pre	4,72	0,50	0,367
miR-21-5p-Post	4,68	0,50	

8. Ethical aspects

The study protocol, informed consent form, and all other study-related documents are submitted to the Ethics Committee of the State of Salzburg. The study will be conducted in accordance with the principles outlined in the Declaration of Helsinki and Good Clinical Practice (GCP) guidelines. Additionally, the study is registered with ClinicalTrials.gov.

Due to the diligent application of relevant safety guidelines, no risks are expected for the participants. Participant information will be provided by a physician according to the informed consent form. The collected data will be processed indirectly with pseudonymization, meaning no directly identifiable personal data will be processed.

9. Insurance

During their participation in the study, the study participants are insured with HDI Versicherung AG under the following policy number 5192355.

10. Study termination

The study can be terminated at any time and without disadvantage to the participating individuals in the event of unforeseen, serious events. There is no predefined termination criterion. Regardless of their motives, individual participants have the right to withdraw from the study at any time, and they will be informed of this option during the informed consent process. A dropout rate of up to one-third is anticipated.

11. Data storage

The data and blood samples will be stored with restricted access. This storage of pseudonymized data will last until complete analysis, or a maximum of 10 years. Protection against unauthorized access is ensured through restricted access to the storage locations and by locking the storage cabinets. If participants disagree with the storage or retention, the data will be deleted, or the sample material will be destroyed. The responsibility for the destruction of the sample material lies with Univ.-Prof. Dr.Dr. Josef Niebauer, MBA. Data processing will occur in pseudonymized form.

12. Data analysis

Data analysis will be performed using IBM SPSS Statistics Software (Version 24.0, SPSS, Inc., Chicago, IL) at the University Institute of Sports Medicine, Prevention and Rehabilitation, Paracelsus Medical University Salzburg. Descriptive statistics will be conducted using mean and standard deviation for normally distributed data and median and range for non-normally distributed data. Two-sided paired t-tests with correction for multiple testing will be used to analyse acute effects of CPET. For non-normally distributed data, a Wilcoxon signed-rank test will be applied accordingly. ANOVA with repeated measures will be used to analyse significant differences between both CPET interventions, resting, and maximum effort values. Depending on the distribution of the data, correlation calculations will be performed using Pearson or Spearman's rank correlation coefficient. A significance level of $p < 0.05$ will be assumed.

13. Safety

Exercise Testing: During the exercise test, participants will cycle to their maximum capacity. To recognize potential health risks before the exercise test, participants will be extensively interviewed about possible contraindications for exercise. In addition, participants will undergo a thorough physical examination, have a resting electrocardiogram recorded, and blood pressure measured. If further examinations are necessary (e.g., echocardiogram, laboratory tests, etc.), they will be conducted. Therefore, there is maximum safety for participants before starting the exercise test. Rarely (<0.01%), incidents such as dangerous cardiac arrhythmias, syncope, or even rarer, death, may occur. Trained, professional personnel will be present throughout the examinations and can respond immediately to incidents.

During physical training, unfavourable events such as significant blood pressure fluctuations, shortness of breath, dizziness, or cardiac arrhythmias may occur. Additionally, in extreme cases, incidents of angina pectoris may occur. Therefore, patients undergoing centre-based training are under continuous medical supervision and train under ECG monitoring. Undesirable risks of training also include musculoskeletal injuries and muscle soreness. The risk for both events can be reduced to a very low level due to qualified supervision and individual training adjustments. To prevent overexertion of the participants, training intensity will be constantly monitored and reduced if necessary.

14. Data security

All data and information collected during the study, as well as all participant-related data, comply with the standards of data protection in accordance with the General Data Protection Regulation (GDPR) of 2016 regarding the handling of personal data. The analysis and summary of the study will be conducted using anonymized data and data without reference to specific study participants.

The use of the aktivplan app and the associated data processing occur on the server of Alphaport OG. Personal (such as name, age) or sensitive data (health data such as maximum heart rate, maximum blood pressure) are stored on the GDPR-certified server provided by Chino.io. To associate users, Alphaport refers to the pseudonymized user ID provided by Chino.io. Through corresponding interfaces, the backend accesses the sensitive data stored at Chino.io using the pseudonymized user ID and outputs them on the frontend.

In the initial phase of the study, study participants are informed about the app and the associated data protection aspects via the app. In particular, the following points are explained and documented with a digital timestamp:

- Objective: The *aktivplan* app aims to support health experts and study participants in individually planning training activities and optimizing them according to the health status of the patients. The app allows study participants to view and track activities planned with health experts on their mobile phones, as well as enter additional activities.
- Procedure: If study participants explicitly agree to use the aktivplan app, the data collected in the performance test will be entered by health experts. This creates a basis for an individually tailored training plan (for example, training in the optimal heart rate zone, with the optimal load). After inputting the data, study participants receive an email with a link. If the link is not clicked, the entered data will be deleted after 10 days. If the link is clicked within 10 days, study participants are informed about the applicable privacy policy (especially the processing of sensitive, health-related data) and the applicable terms and conditions. After the study participants agree to the above points, the aktivplan app can be installed on their mobile phones.
- Data protection and data utilization: Personal data is pseudonymized and stored on secure servers (GDPR compliant). The recorded data is used to create an individually tailored and optimally matched training program based on the health status (for example, training in the optimal heart rate zone).
- Voluntary participation and freedom to withdraw: The choice to use the *aktivplan* app is entirely voluntary. If study participants do not click the link in the email received for app registration within 10 days, the recorded data will be automatically deleted. After registration, the use of the *aktivplan* app can be terminated at any time, including the deletion of all data of the study participant.

- Questions: If there are any questions, contact can be made at any time.

15. Reporting

Final report

A final report on this study will be written, regardless of the results. This report will be submitted to the responsible officials of the Prosperamus! funding agency as well as the relevant ethics committee.

Publication

The aim is to publish the results of the study in a scientific journal with a peer-review process. The ethics committee will be informed about the final results of the study.

16. Reference

1. *Global, regional, and national age-sex specific all-cause and cause-specific mortality for 240 causes of death, 1990-2013: a systematic analysis for the Global Burden of Disease Study 2013.* Lancet, 2015. **385**(9963): p. 117-71.
2. Symonds, M.E., S. Sebert, and H. Budge, *The obesity epidemic: from the environment to epigenetics - not simply a response to dietary manipulation in a thermoneutral environment.* Front Genet, 2011. **2**: p. 24.
3. Schulte, C. and T. Zeller, *microRNA-based diagnostics and therapy in cardiovascular disease-Summing up the facts.* Cardiovasc Diagn Ther, 2015. **5**(1): p. 17-36.
4. Sun, X., N. Belkin, and M.W. Feinberg, *Endothelial microRNAs and atherosclerosis.* Curr Atheroscler Rep, 2013. **15**(12): p. 372.
5. Santulli, G., *microRNAs and Endothelial (Dys) Function.* Journal of cellular physiology, 2015.
6. Nabel, E.G. and E. Braunwald, *A tale of coronary artery disease and myocardial infarction.* N Engl J Med, 2012. **366**(1): p. 54-63.
7. Baggish, A.L., et al., *Dynamic regulation of circulating microRNA during acute exhaustive exercise and sustained aerobic exercise training.* J Physiol, 2011. **589**(Pt 16): p. 3983-94.
8. Mayr, B., et al., *Exercise responsive micro ribonucleic acids identify patients with coronary artery disease.* Eur J Prev Cardiol, 2019. **26**(4): p. 348-355.
9. Goodman, J., S. Thomas, and J.F. Burr, *Physical activity series: cardiovascular risks of physical activity in apparently healthy individuals: risk evaluation for exercise clearance and prescription.* Can Fam Physician, 2013. **59**(1): p. 46-9, e6-e10.
10. Whitfield, G.P., et al., *Trends in Meeting Physical Activity Guidelines Among Urban and Rural Dwelling Adults - United States, 2008-2017.* MMWR Morb Mortal Wkly Rep, 2019. **68**(23): p. 513-518.
11. Bull, F.C., et al., *World Health Organization 2020 guidelines on physical activity and sedentary behaviour.* Br J Sports Med, 2020. **54**(24): p. 1451-1462.
12. Assmann, G., P. Cullen, and H. Schulte, *Simple scoring scheme for calculating the risk of acute coronary events based on the 10-year follow-up of the prospective cardiovascular Munster (PROCAM) study.* Circulation, 2002. **105**(3): p. 310-5.
13. Expert Panel on Detection, E. and A. Treatment of High Blood Cholesterol in, *Executive Summary of The Third Report of The National Cholesterol Education Program (NCEP) Expert Panel on Detection, Evaluation, And Treatment of High Blood Cholesterol In Adults (Adult Treatment Panel III).* JAMA, 2001. **285**(19): p. 2486-97.
14. Perk, J., et al., *European Guidelines on cardiovascular disease prevention in clinical practice (version 2012). The Fifth Joint Task Force of the European Society of Cardiology and Other Societies on Cardiovascular Disease Prevention in Clinical Practice (constituted by representatives of nine societies and by invited experts).* Eur Heart J, 2012. **33**(13): p. 1635-701.

15. Weber, T., et al., *Wave reflections, assessed with a novel method for pulse wave separation, are associated with end-organ damage and clinical outcomes.* Hypertension, 2012. **60**(2): p. 534-41.
16. Boutouyrie, P., et al., *Aortic stiffness is an independent predictor of primary coronary events in hypertensive patients: a longitudinal study.* Hypertension, 2002. **39**(1): p. 10-5.
17. Cruickshank, K., et al., *Aortic pulse-wave velocity and its relationship to mortality in diabetes and glucose intolerance: an integrated index of vascular function?* Circulation, 2002. **106**(16): p. 2085-90.
18. Silverman, M.G., et al., *Circulating miRNAs and Risk of Sudden Death in Patients With Coronary Heart Disease.* JACC Clin Electrophysiol, 2020. **6**(1): p. 70-79.
19. Baggish, A.L., et al., *Rapid up-Regulation and Clearance of Distinct Circulating Micrornas after Prolonged Aerobic Exercise.* Cardiology, 2014. **128**: p. 442-442.
20. Liu, Y., et al., *The role of circulating microRNA-126 (miR-126): a novel biomarker for screening prediabetes and newly diagnosed type 2 diabetes mellitus.* Int J Mol Sci, 2014. **15**(6): p. 10567-77.
21. Zhou, J., et al., *Associations between physical activity-related miRNAs and metabolic syndrome.* Horm Metab Res, 2014. **46**(3): p. 201-5.
22. Uhlemann, M., et al., *Circulating microRNA-126 increases after different forms of endurance exercise in healthy adults.* Eur J Prev Cardiol, 2014. **21**(4): p. 484-91.
23. Wardle, S.L., et al., *Plasma microRNA levels differ between endurance and strength athletes.* PLoS One, 2015. **10**(4): p. e0122107.
24. Cheng, Y., et al., *Ischaemic preconditioning-regulated miR-21 protects heart against ischaemia/reperfusion injury via anti-apoptosis through its target PDCD4.* Cardiovasc Res, 2010. **87**(3): p. 431-9.
25. Thum, T., et al., *MicroRNA-21 contributes to myocardial disease by stimulating MAP kinase signalling in fibroblasts.* Nature, 2008. **456**(7224): p. 980-4.
26. Sawada, S., et al., *Profiling of circulating microRNAs after a bout of acute resistance exercise in humans.* PLoS One, 2013. **8**(7): p. e70823.
27. Nielsen, S., et al., *The miRNA plasma signature in response to acute aerobic exercise and endurance training.* PLoS One, 2014. **9**(2): p. e87308.
28. Wonisch, M., et al., *Praxisleitlinien Ergometrie.* Atemwegs- und Lungenkrankheiten, 2014. **40**.
